# Repurposing the β_3_-Adrenergic Receptor Agonist Mirabegron in Patients With Structural Cardiac Disease: The Beta3-LVH Phase 2b Randomized Clinical Trial

**DOI:** 10.1001/jamacardio.2023.3003

**Published:** 2023-09-20

**Authors:** Jean-Luc Balligand, Dulce Brito, Oana Brosteanu, Barbara Casadei, Christophe Depoix, Frank Edelmann, Vanessa Ferreira, Gerasimos Filippatos, Bernhard Gerber, Damien Gruson, Dirk Hasenclever, Kristian Hellenkamp, Ignatios Ikonomidis, Bartosz Krakowiak, Renaud Lhommel, Masliza Mahmod, Stefan Neubauer, Alexandre Persu, Stefan Piechnik, Burkert Pieske, Elisabeth Pieske-Kraigher, Fausto Pinto, Piotr Ponikowski, Michele Senni, Jean-Noël Trochu, Nancy Van Overstraeten, Rolf Wachter, Anne-Catherine Pouleur

**Affiliations:** 1 Institut de Recherche Expérimentale et Clinique, Cliniques Universitaires Saint-Luc, Université Catholique de Louvain, Brussels, Belgium; 2 Department of Cardiology, Centro Hospitalar Universitário Lisboa Norte, Lisboa, Portugal; 3 Centro Académico de Medicina de Lisboa, Universidade de Lisboa, Lisboa, Portugal; 4 Faculdade de Medicina, Centro Cardiovascular, Universidade de Lisboa, Lisboa, Portugal; 5 Clinical Trial Centre Leipzig, Universität Leipzig, Leipzig, Germany; 6 Division of Cardiovascular Medicine, Radcliffe Department of Medicine, British Heart Foundation Centre of Research Excellence, University of Oxford, Oxford, United Kingdom; 7 National Institute of Health Research Oxford Biomedical Research Centre, John Radcliffe Hospital, Oxford, United Kingdom; 8 Department of Cardiology, German Centre for Cardiovascular Research, Charité University Campus Virchow, Berlin, Germany; 9 Radcliffe Department of Medicine, Oxford Centre for Clinical Magnetic Resonance Research, University of Oxford, Oxford, United Kingdom; 10 Department of Cardiology, School of Medicine, National and Kapodistrian University of Athens, Attikon University Hospital, Athens, Greece; 11 Institute for Medical Informatics, Statistics, and Epidemiology, Universität Leipzig, Leipzig, Germany; 12 Department of Cardiology and Pneumology, German Centre for Cardiovascular Research, Universitätsmedizin Göttingen, Göttingen, Germany; 13 Department of Cardiology, Centre for Heart Diseases, Clinical Military Hospital, Wrocław Medical University, Wrocław, Poland; 14 Department of Cardiology, Azienda Socio Sanitaria Territoriale Papa Giovanni XXIII, University of Milano-Bicocca, Bergamo, Italy; 15 Institut du Thorax, Centre National de la Recherche Scientifique, Nantes Université, Nantes, France; 16 L’Institut National de la Santé et de la Recherche Médicale, Centre Hospitalier Universitaire de Nantes, Nantes Université, Nantes, France; 17 Department of Cardiology, University Hospital Leipzig, Leipzig, Germany

## Abstract

**Importance:**

Left ventricular (LV) hypertrophy contributes to the onset and progression of heart failure (HF), particularly for patients with pre-HF (stage B) for whom no treatment has yet proven effective to prevent transition to overt HF (stage C). The β_3_-adrenergic receptors (β3ARs) may represent a new target, as their activation attenuates LV remodeling.

**Objective:**

To determine whether activation of β3ARs by repurposing a β3AR agonist, mirabegron, is safe and effective in preventing progression of LV hypertrophy and diastolic dysfunction among patients with pre- or mild HF.

**Design, Setting, and Participants:**

The Beta3-LVH prospective, triple-blind, placebo-controlled phase 2b randomized clinical trial enrolled patients between September 12, 2016, and February 26, 2021, with a follow-up of 12 months. The trial was conducted at 10 academic hospitals in 8 countries across Europe (Germany, Poland, France, Belgium, Italy, Portugal, Greece, and the UK). Patients aged 18 years or older with or without HF symptoms (maximum New York Heart Association class II) were screened for the presence of LV hypertrophy (increased LV mass index [LVMI] of ≥95 g/m^2^ for women or ≥115 g/m^2^ for men) or maximum wall thickness of 13 mm or greater using echocardiography. Data analysis was performed in August 2022.

**Intervention:**

Participants were randomly assigned (1:1) to mirabegron (50 mg/d) or placebo, stratified by the presence of atrial fibrillation and/or type 2 diabetes, for 12 months.

**Main Outcomes and Measures:**

The primary end points were LVMI determined using cardiac magnetic resonance imaging and LV diastolic function (early diastolic tissue Doppler velocity [E/e′] ratio assessed using Doppler echocardiography) at 12 months. Patients with at least 1 valid measurement of either primary end point were included in the primary analysis. Safety was assessed for all patients who received at least 1 dose of study medication.

**Results:**

Of the 380 patients screened, 296 were enrolled in the trial. There were 147 patients randomized to mirabegron (116 men [79%]; mean [SD] age, 64.0 [10.2] years) and 149 to placebo (112 men [75%]; mean [SD] age, 62.2 [10.9] years). All patients were included in the primary intention-to-treat analysis. At 12 months, the baseline and covariate-adjusted differences between groups included a 1.3-g/m^2^ increase in LVMI (95% CI, −0.15 to 2.74; *P* = .08) and a −0.15 decrease in E/e′ (95% CI, −0.69 to 0.4; *P* = .60). A total of 213 adverse events (AEs) occurred in 82 mirabegron-treated patients (including 31 serious AEs in 19 patients) and 215 AEs occurred in 88 placebo-treated patients (including 30 serious AEs in 22 patients). No deaths occurred during the trial.

**Conclusions:**

In this study, mirabegron therapy had a neutral effect on LV mass or diastolic function over 12 months among patients who had structural heart disease with no or mild HF symptoms.

**Trial Registration:**

ClinicalTrials.gov Identifier: NCT02599480

## Key Points

**Question:**

Does activation of β_3_-adrenergic receptors prevent progression of left ventricular hypertrophy and diastolic dysfunction among patients with pre- (stage B) or mild heart failure?

**Findings:**

In this randomized clinical trial of 296 adults, changes in left ventricular mass or diastolic function over 12 months were not statistically different between patients receiving the current therapeutic dosage of β3-adrenergic receptor agonist mirabegron (50 mg/d) or placebo.

**Meaning:**

The therapeutic dosage of mirabegron had a neutral effect on left ventricular mass and diastolic function among patients with pre- or mild heart failure.

## Teaser

This randomized clinical trial evaluates repurposing mirabegron, a β_3_-adrenergic receptor agonist, to prevent progression of left ventricular hypertrophy and diastolic dysfunction among patients with pre- or mild heart failure.

## Introduction

Heart failure (HF) is a major public health burden that affects 2% to 3% of adults in high-income countries.^1,2^ For up to half of patients, HF evolves with a mildly reduced or preserved ejection fraction (HFpEF); this proportion is expected to grow as the aging population increases. Older individuals and patients with hypertension, diabetes, and obesity are at particularly high risk of HF, including HFpEF. The universal HF classification^3^ and the 2022 American College of Cardiology/American Heart Association staging models^2^ emphasize the identification of such patients at risk of HF (stage A) or pre-HF (stage B) without and with evidence of cardiac remodeling, respectively, to promote preventive action before progression to symptomatic HF (stage C). Thus, interventions for patients with asymptomatic pre-HF may be important in reducing the incidence of clinically overt HF.

Despite the growing incidence of HFpEF over the last 20 years, the few therapies proven to be effective thus far were mainly tested a posteriori (ie, in patients with symptomatic HF).^4,5,6^ Given known effects of cyclic guanosine monophosphate (cGMP) on vasodilation, anti-remodeling, and lusitropic properties on the myocardium, previous efforts aimed to increase cGMP–protein kinase G (PKG) signaling, among other possible targets. Although the results of previous trials with soluble guanylyl cyclase stimulators or phosphodiesterase 5 inhibitors have been disappointing for patients with established HFpEF,^7,8^ the efficacy of these approaches may have been hampered by low bioavailability of the nitric oxide (NO) produced and its associated cGMP pool, due to the prevailing oxidative stress in advanced disease.^9^ Whether any of these drugs prevent the development of HFpEF at earlier stages has not been confirmed.

In this context, activation of the cardiac β_3_-adrenergic receptor (β3AR) may offer alternative, more efficient pharmacodynamic activation of the cGMP/PKG pathway. Indeed, β3AR stimulation produces antioxidant effects that are expected to preserve NO/cGMP bioavailability and possibly protect other molecular targets that regulate excitation-contraction coupling and myocardial remodeling.^10^ In preclinical studies, β3AR stimulation coupled to the NO/cGMP pathway, resulting in coronary vasodilatation and increased cGMP content in human myocardium, with a subsequent effect on cardiac myocytes that was opposite of classical β1-2AR effects.^11^ Activation of β3AR/NO decreases myocardial hypertrophy and fibrosis in response to neurohormonal or hemodynamic stresses without compromising left ventricular (LV) function,^12^ and cGMP/PKG-Ia promotes cardiac relaxation through phosphorylation of titin, troponin I, and sarcoendoplasmic reticulum calcium ATPase.^13^ Because ample evidence points to the adverse effects of sustained β1-2AR activation, we reasoned that activation of the functionally opposed β3AR/NO pathway would protect against such deleterious effects of chronic adrenergic stimulation, as typically observed in patients with hypertension or obesity who have structural heart disease and are at risk of developing HF.^14^

Medications that specifically activate the human β3AR, such as mirabegron, have recently been developed and marketed for clinical use in overactive bladder disease. Mirabegron had few cardiovascular effects in previous urologic studies.^15^ However, patients with high cardiovascular risk were excluded per protocol in those trials, so questions remain regarding the innocuity of mirabegron in this specific population. Therefore, the Beta3-LVH trial tested the hypothesis that repurposing mirabegron for patients at risk of or with mild HF is safe and protects against worsening LV hypertrophy (LVH) and/or diastolic dysfunction.

## Methods

### Study Design

The Beta3-LVH is a prospective, parallel, placebo-controlled, triple-blind phase 2b randomized clinical trial that was performed in 10 academic hospitals in Germany (3 centers), Poland, France, Belgium, Italy, Portugal, Greece, and the UK. Complete details on the trial protocol were reported previously^16^ and are presented in Supplement 1 and in the eMethods in Supplement 2. The study was approved by the ethics committees in all participating countries. Written informed consent was obtained from all participants. An independent data safety monitoring board reviewed unblinded trial data prepared by an independent statistician on a regular basis and issued recommendations on trial continuation. The study followed the Consolidated Standards of Reporting Trials (CONSORT) reporting guideline.

### Participants

Men and women aged older than 18 years were invited to participate if they had an increased LV mass index (LVMI) (≥115 g/m^2^ for men and ≥95 g/m^2^ for women) or end-diastolic wall thickness of 13 mm or greater in at least 1 wall segment, in the absence of inherited hypertrophic cardiomyopathy or substantial valvular disease at screening echocardiography. Participants were screened for additional inclusion and exclusion criteria between September 12, 2016, and February 26, 2021 (eTable 1 in Supplement 2). Patients with hypertension were required to receive stable therapy for at least 4 weeks before inclusion and to have well-controlled blood pressure. Patients with documented ischemic cardiac disease (eg, history of acute myocardial infarction, current angina pectoris, or known coronary disease) or atrial fibrillation with an uncontrolled heart rate (>100 beats/min) were excluded. All patients with an ejection fraction (EF) of less than 50% or with New York Heart Association (NYHA) functional class symptoms greater than class II were also excluded.

### Randomization

Patients were randomized (1:1) to mirabegron (50 mg/d) or placebo, stratified by the presence or absence of type 2 diabetes and atrial fibrillation at randomization. This dosage was chosen on the basis of safety data from previous clinical trials, as allowed by ethics committees in all participating countries. Allocation concealment and randomization were ensured centrally through a secure web-based tool held by Universität Leipzig using a modified minimization procedure with a stochastic component, according to Pocock and Simon.^17^

### Procedures

A trial flow diagram is presented in eFigure 1 in Supplement 2. Eligible patients underwent a baseline evaluation consisting of the following: medical history, clinical examination (including blood pressure measurement), 24-hour ambulatory blood pressure monitoring, determination of NYHA functional class, body mass index (BMI [calculated as weight in kilograms divided by height in meters squared]), electrocardiography, transthoracic echocardiography, and cardiac magnetic resonance imaging (MRI) (LV volume, left atrial [LA] volume, LV mass, LV function, LV wall thickness, late gadolinium enhancement, and T1 mapping). All imaging procedures were performed according to a standardized operational protocol designed by the echocardiography and cardiac MRI core laboratories at Charité University Berlin and the University of Oxford, respectively, and parameters were measured centrally in the respective core laboratories. Patients also performed a cardiopulmonary exercise test on a cycloergometer and underwent urine and blood sampling for measurements of fasting glucose, insulin (homeostatic model assessment [HOMA] of sensitivity), hemoglobin A_1c_, serum lipids, galectin-3, growth differentiation factor 15, N-terminal prohormone of brain natriuretic peptide (NT-proBNP), and high-sensitivity troponin I. Additional procedures, including substudies on brown fat activity by fluorodeoxyglucose positron emission tomography and endothelial function by digital microtonometry, are described in the eMethods in Supplement 2.

### Outcomes: Primary and Secondary End Points

To assess both structural and functional aspects of LV remodeling, 2 equally ranked primary end points were chosen: (1) a change in LVMI (in grams per meters squared) measured with cardiac MRI at baseline, 6 months, and 12 months after randomization; and (2) a change in diastolic function, assessed as the ratio of peak early transmitral ventricular filling velocity to early diastolic tissue Doppler velocity (E/e′) measured with echocardiography at baseline, 6 months, and 12 months after randomization.

The following key secondary end points were evaluated: interstitial cardiac fibrosis (extracellular volume fraction), LA volume index, LV stroke volume index, right ventricular (RV) EF (all by cardiac MRI), maximal exercise capacity (peak oxygen consumption), insulin sensitivity (HOMA), and NT-proBNP at 12 months. Additional secondary end points are listed in the eMethods in Supplement 2.

Investigators duly documented and reported all adverse events (AEs), including serious AEs, to the Universität Leipzig Clinical Trial Centre, which passed them immediately to the trial coordinator for medical assessment and a second opinion with respect to the causal relationship with the trial medication. All study procedures followed good clinical practice rules.

### Statistical Analysis

The Beta3-LVH trial tested the hypothesis that, compared with placebo, mirabegron as an add-on to standard treatment improves at least 1 of the 2 primary end points over 12 months. The Hochberg method was used to adjust for end-point multiplicity; that is, efficacy was claimed for both primary end points if both *P* values were below .05 or for the respective primary end point if the smallest *P* value was below .025. This procedure controlled the familywise error rate at a 2-sided significance level of 5%.^18^

Sample size calculation was based on the E/e′ ratio, with the aim to detect a difference of 1.2 between the 2 treatment groups. This roughly corresponds to 5 points on the Short Form-36 physical functional scale, a patient-relevant difference.^19^ With an SD of 3, a total of 272 patients analyzed would result in 85% power at a significance level of 2.5% using a 2-sided *t* test using nQuery Advisor, version 7.0 (Dotmatics).

Analyses of both primary and secondary end points were identically structured. Mean changes from baseline were analyzed using a repeated-measures linear mixed model without the intercept containing the fixed, categorical effects of visit (baseline, 6 months, or 12 months), treatment (active or placebo), treatment-by-visit interaction, atrial fibrillation (yes or no), type 2 diabetes (yes or no), and a patient-specific visit random effect (3-dimensional normal with a general unstructured variance covariance matrix). The model implicitly used the estimated covariance matrix to deal with missed visits. Assuming that missing end points are missing at random, the model can deal with patients with incomplete end-point data provided that at least 1 valid measurement is documented. Given randomization at baseline, the treatment by baseline interaction was not included in the model. This indirectly adjusted for possible random chance fluctuation at baseline and generally increased the power.^20^ The model was fitted using the nlme package in R, version 4.2.0 (R Foundational for Statistical Computing) (complete formula in the eMethods in Supplement 2).

The per-protocol set included all patients from the full analysis set without major violations of the study protocol. The population in the safety analysis set was defined by all randomized patients belonging to the full analysis set who received at least 1 dose of study medication.

Exploratory subgroup analyses for the 2 primary end points were performed with baseline characteristics, including sex, β-blocker use in standard treatment, type 2 diabetes, atrial fibrillation at registration, age older than 65 years, BMI greater than 30 at baseline, and region (Poland, Germany, or other countries).

We used χ^2^ tests to compare AEs and serious AEs, with special attention to the occurrence of high blood pressure, hepatic impairment, kidney impairment, atrial fibrillation or flutter, and death. Data analysis was performed in August 2022.

## Results

**Figure 1.**
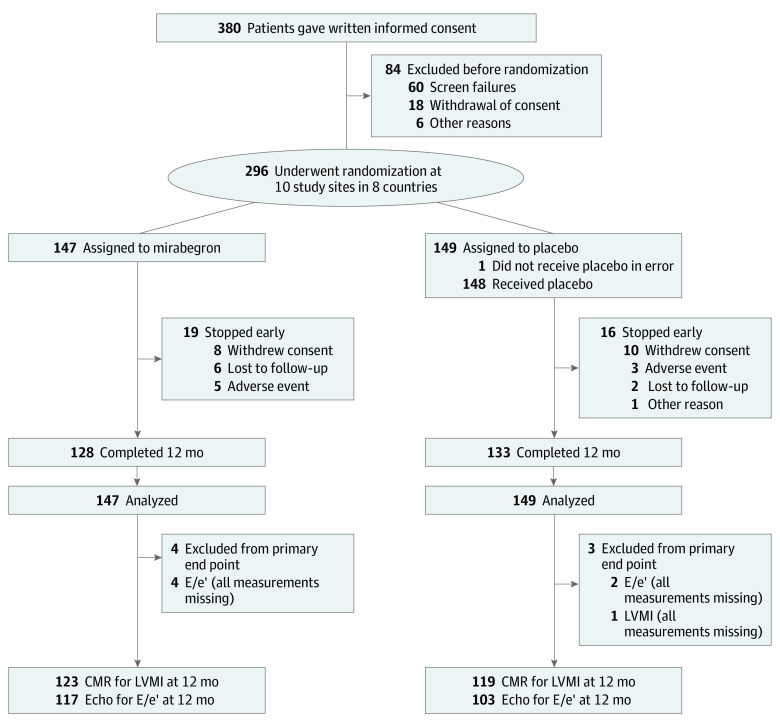
Trial Flow Diagram CMR indicates cardiac magnetic resonance imaging; E/e′, ratio of peak early transmitral ventricular filling velocity to early diastolic tissue Doppler velocity; LVMI, left ventricular mass index.

Of the 380 patients screened, 60 did not meet the inclusion criteria, 18 declined to take part, and 6 were not recruited for other reasons (Figure 1). Therefore, 296 patients were considered eligible and randomized to either mirabegron or placebo. The mirabegron-treated group included 147 patients (116 men [79%] and 31 women [21%]), with a mean (SD) age of 64.0 (10.2) years. The placebo-treated group included 149 patients (112 men [75%] and 37 women [25%]), with a mean (SD) age of 62.2 (10.9) years. A total of 35 patients (19 in the mirabegron group and 16 in the placebo group) dropped out of the trial (detailed in Figure 1). All randomized patients with at least 1 valid measurement of either LVMI or E/e′ were included in the full analysis set. Of these, 1 patient in the placebo group had no LVMI measurement because of contraindication for MRI but had a valid echocardiographic E/e′ recording. Two more patients in the placebo group and 4 patients in the mirabegron group had no valid E/e′ recording but had valid LVMI measurements. Further description of the per-protocol set is included in the eResults in Supplement 2.

Demographic and clinical characteristics were well balanced between the treatment groups at baseline (Table 1). Overall, the mean (SD) BMI was 30 (4.3); more than 90% of mirabegron- and placebo-treated patients had hypertension (137 [93%] and 137 [92%], respectively) and approximately 20% had type 2 diabetes (26 [18%] and 31 [21%], respectively). According to the study protocol, patients were receiving background cardiovascular therapies that did not differ between groups, including angiotensin-converting enzyme inhibitors and/or angiotensin receptor blockers, diuretics, and calcium antagonists. Approximately 50% of the mirabegron- and placebo-treated groups (80 [54.4%] and 75 [50.7%]) were treated with β blockers. Most participants had no HF symptoms; only 20 mirabegron-treated patients (13.6%) and 18 placebo-treated patients (12.1%) had NYHA class II symptoms. Adherence to trial medication was excellent in both treatment groups, with a relative dose of greater than 80% for 119 mirabegron-treated patients (81.0%) and 118 placebo-treated patients (79.2%) (eTable 2 in Supplement 2).

**Table 1.** Baseline Patient Characteristics

Characteristic	Patient group	
	Mirabegron (n = 147)	Placebo (n = 149)
Age, y, mean (SD)	64.0 (10.2)	62.2 (10.9)
Sex, No. of patients (%)		
Male	116 (79)	112 (75)
Female	31 (21)	37 (25)
BMI, mean (SD)	29.8 (4.3)	30.1 (4.3)
NYHA functional class, No. of patients (%)		
II	20 (13.6)	18 (12.1)
I	7 (4.8)	7 (4.7)
No symptoms	120 (81.6)	124 (83.2)
LVEF by echocardiography, %, mean (SD)	61.5 (5.6)	62.5 (6.6)
E/e′, mean (SD)	9.4 (3.2)	9.7 (3.0)
LVMI, g/m^2^, mean (SD)		
Assessed with echocardiography		
Men	124 (28)	126 (33)
Women	115 (24)	114 (23)
Assessed with cardiac MRI		
Men	62.4 (12.2)	61.4 (10.3)
Women	51.4 (9.9)	51.3 (9.4)
LAVI assessed with MRI, mL/m^2^, mean (SD)	43.0 (14.1)	41.8 (14.7)
Peak oxygen consumption, mL/min/kg, mean (SD)	19.6 (5.4)	19.1 (5.3)
eGFR, mL/min, mean (SD)	85.1 (21.2)	83.6 (21.3)
NT-proBNP, pg/mL, median (IQR)	69.0 (32.7-137.9)	65.0 (37.0-119.0)
Cholesterol, mg/dL, mean (SD)		
Total	170 (37)	177 (43)
Low-density lipoprotein	95 (31)	100 (39)
High-density lipoprotein	48 (13)	46 (13)
Triglycerides, mg/dL, mean (SD)	128 (65)	153 (129)
HOMA of insulin sensitivity, %, median (IQR)	63.5 (45.3-96.0)	61.5 (42.5-94.6)
Cardiovascular risk factors and medical history, No. of patients (%)		
Hypertension	137 (93)	137 (92)
Type 2 diabetes	26 (18)	31 (21)
Atrial fibrillation	13 (8.8)	13 (8.7)
Medication, No. of patients (%)		
ACE inhibitor	66 (44.9)	76 (51.4)
ARB	54 (36.7)	49 (33.3)
β-Blocker	80 (54.4)	75 (50.7)
Calcium channel blocker	57 (38.8)	64 (43.2)
Loop diuretic	10 (6.8)	13 (8.8)
Aldosterone antagonist	11 (7.5)	14 (9.5)
Thiazide	33 (22.4)	20 (13.5)

Abbreviations: ACE, angiotensin-converting enzyme; ARB, angiotensin receptor blocker; BMI, body mass index (calculated as weight in kilograms divided by height in meters squared); E/e′, ratio of peak early transmitral ventricular filling velocity to early diastolic tissue Doppler velocity; eGFR, estimated glomerular filtration rate (using the Modification of Diet in Renal Disease formula); HOMA, homeostatic model assessment; LAVI, left atrial volume index; LVEF, left ventricular ejection fraction; LVMI, left ventricular mass index; MRI, magnetic resonance imaging; NT-proBNP, N-terminal prohormone of brain natriuretic peptide; NYHA, New York Heart Association.

SI conversion factors: To convert cholesterol and triglycerides to mmol/L, multiply values by 0.0259 and 0.0113, respectively.

**Figure 2.**
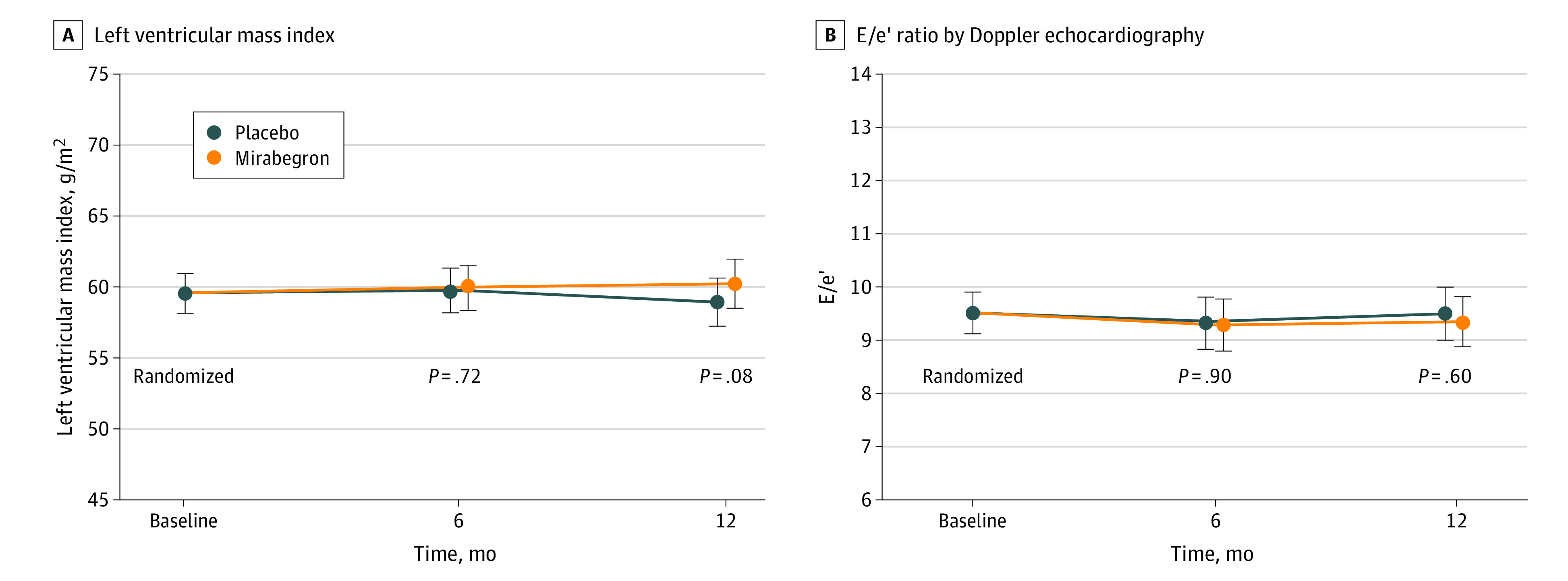
Change in Primary Outcomes Over Time in the Mirabegron and Placebo Groups A and B, Treatment-specific changes in left ventricular mass index (A) and E/e′ ratio (B) at postbaseline visits, deduced from the basic linear mixed model. Because the groups were randomized, the mean at baseline was not estimated separately by group. *P* values refer to treatment differences tested against 0.

**Figure 3.**
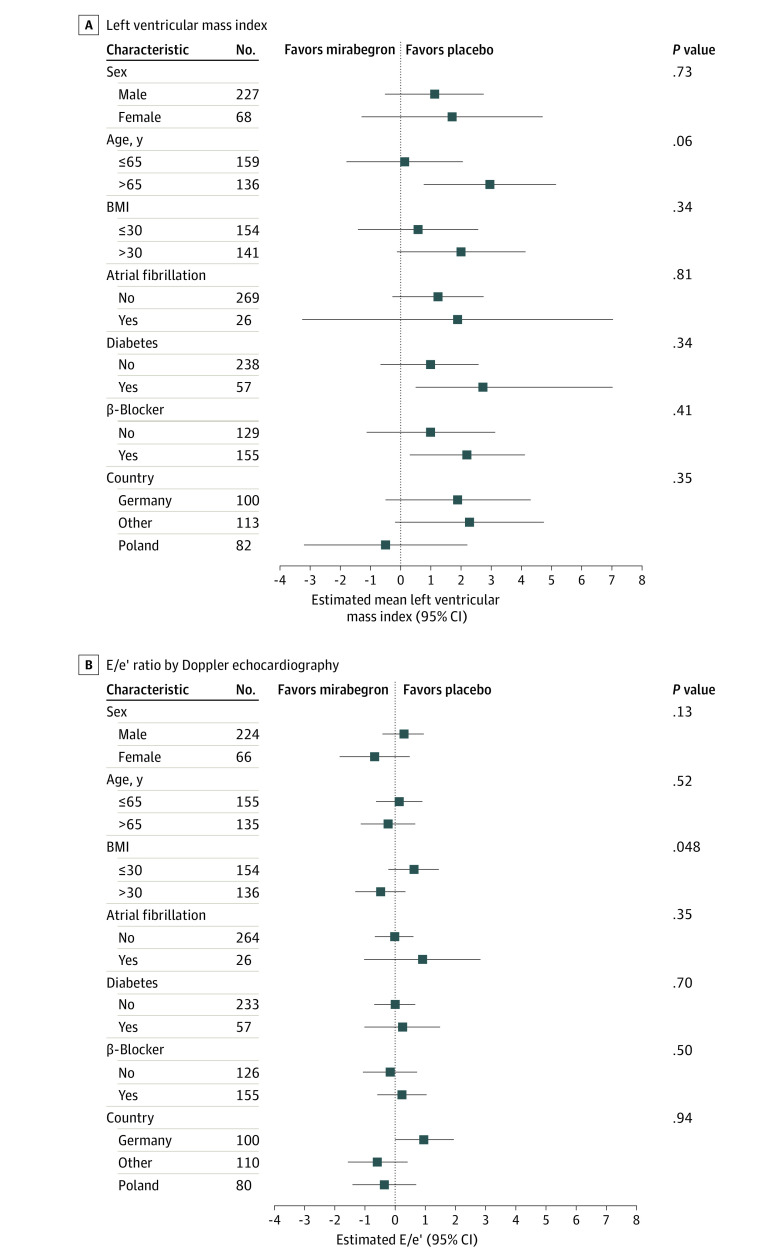
Prespecified Subgroup Analyses A and B, Results of prespecified explorative subgroup analyses for the left ventricular mass index (A) and E/e′ ratio (B) primary end points. The following subgroups were analyzed: age (≤65 or >65 years at baseline), sex, body mass index (BMI [calculated as weight in kilograms divided by height in meters squared] of ≤30 or >30 at baseline), presence of diabetes, atrial fibrillation, β-blocker use as standard treatment, and geographic region. The interaction test *P* value refers to the null hypothesis that the treatment effects are the same across subgroups. *P* values are not multiplicity adjusted.

Neither primary outcome reached the predefined statistical significance level. At 12 months, baseline and covariate-adjusted differences between groups included a 1.3-g/m^2^ increase in LVMI (95% CI, −0.15 to 2.74; *P* = .08) and a −0.15 decrease in E/e′ (95% CI, −0.69 to 0.4; *P* = .60) (Figure 2 and eTables 3 and 4 in Supplement 2). Similar results were obtained in the per-protocol analysis (eFigure 2 in Supplement 2). The effect of mirabegron remained neutral in analysis of exploratory subgroups, including age (≤65 or >65 years at baseline), sex (men or women), BMI (≤30 or >30 at baseline), presence of type 2 diabetes, atrial fibrillation, β-blocker use, and geographic region (Figure 3).

eFigure 3 in Supplement 2 shows the comparative effects of mirabegron and placebo on the main prespecified secondary outcomes. No statistically significant differences between treatment groups were observed for these secondary outcomes. The effects on other secondary end points are illustrated in eFigure 4 in Supplement 2. The effect of mirabegron was neutral on all of these additional end points except for a nominal decrease in high-sensitivity troponin I at 12 months (−0.21 [95% CI, −0.36 to −0.05]; *P* < .01).

A total of 428 AEs were recorded (213 and 215 in the mirabegron and placebo groups, respectively). These AEs affected a total of 170 patients (82 and 88 in the mirabegron and placebo groups, respectively; Table 2). Details are reported in Supplement 2.

**Table 2.** Adverse Events

AE	Patient group			
	Mirabegron (n = 148)		Placebo (n = 148)	
	No. (%) of patients with an AE	Total AEs	No. (%) of patients with an AE	Total AEs
Any	82 (55.4)	213	88 (59.5)	215
Serious	19 (12.8)	31	22 (14.9)	30
Any leading to permanent treatment discontinuation	7 (4.7)	11	9 (6.1)	17
Any leading to treatment interruption	9 (6.1)	16	9 (6.1)	10
Arrhythmia (atrial fibrillation, flutter)	7 (4.7)	17	3 (2.0)	4
High blood pressure (systolic or diastolic)	21 (14.2)	34	19 (12.8)	29
Kidney insufficiency (eGFR <30 mL/min per 1.73 m^2^)	0	0	1 (0.7)	1
Liver enzyme elevation (ALT or AST >2 times ULN)	5 (3.4)	6	2 (1.4)	2

Abbreviations: AE, adverse event; ALT, alanine transaminase; AST, aspartate aminotransferase; eGFR, estimated glomerular filtration rate (using the Modification of Diet in Renal Disease formula); ULN, upper limit of normal.

There were no deaths reported during the trial. The specific incidence of elevated blood pressure, atrial fibrillation or flutter, and kidney or hepatic function impairment in the 2 groups is reported in eTables 5 and 6 in Supplement 2. No statistically significant between-group differences in the occurrence of these AEs were observed over the trial duration. Additional results, including substudies on beige or brown fat and endothelial function, are reported in the eResults in Supplement 2.

## Discussion

Changes in the primary end points of LVMI or E/e′ over 1 year of treatment did not differ between the 2 Beta3-LVH groups treated with mirabegron or placebo. The current therapeutic mirabegron dosage (50 mg/d) did not cause harm in a selected patient population at risk of developing or worsening HF.

Thus far, very few clinical trials have tested the effect of mirabegron on cardiovascular outcomes. Two pilot studies (Beta 3 Agonist Treatment in Heart Failure I and II [BEAT-HF I and BEAT-HF II]) evaluated the effect of high dosages of mirabegron (300 mg/d) on LV function in patients with heart failure with reduced ejection fraction (HFrEF).^21,22^ In the BEAT-HF I trial, the effect of 6 months of mirabegron treatment (300 mg/d; under β1-2AR blockade to minimize off-target effects) was neutral. A non-prespecified subgroup analysis suggested a positive effect of the medication on LVEF in patients with more severe HF. The BEAT-HF II trial specifically tested the effect of short-term (1-week) treatment with the same dosage of mirabegron on hemodynamic parameters in hospitalized patients with severe HFrEF (NYHA class III-IV). The results showed a substantial improvement in LV function in this acute setting, but long-term data are not available. The results of the SPHERE trial were published recently, in which the effect of mirabegron (titrated up to 250 mg/d, with a majority of patients tolerating 150 mg/d) was tested against placebo on the primary end point of pulmonary vascular resistance in patients with (pre-and postcapillary) pulmonary hypertension originating from HFrEF or HFpEF.^23^ The study did not meet its primary end point but showed a positive effect of mirabegron on the prespecified secondary outcome of RVEF. The underlying hypotheses tested in these trials were based on rigorous preclinical studies, including large animal models of HF or pulmonary hypertension. Notably, all of these studies identified antioxidant or vasodilatory effects of β3AR activation as a putative mechanism of improvement in hemodynamic parameters. None tested the long-term influence on cardiovascular remodeling.

To our knowledge, Beta3-LVH is the first trial to test the longer-term effects of mirabegron (over 12 months) in a patient population with pre-HF structural heart disease (stage B) at risk of developing (or worsening) HF,^2,3^ particularly HF with preserved or minimally reduced EF. Based on preclinical evidence of antihypertrophic and antifibrotic effects of myocardial β3ARs, the trial tested the hypothesis that mirabegron would prevent the progression of adverse remodeling toward HFpEF. As such, it entailed thorough characterization of morphologic, functional, and biologic parameters in a selected patient population, undertaking cardiac MRI and Doppler echocardiography that followed a standardized protocol and analyzed the data centrally, with a focus on LVMI and E/e′ as primary end points. The original sample size calculation was based on published data on E/e′ as a parameter of diastolic function.^24,25^ Despite the COVID-19 pandemic, we enrolled our target of 296 patients, randomized them to treatment, and analyzed the data in the intention-to-treat analysis.

Compared with previous literature on similar patients, our study population had lower LVMI and E/e′ at baseline, which left limited room for treatment-specific improvement. Despite our selection of primarily patients with hypertension (>90%), LVMI (determined with cardiac MRI analysis in the core laboratory) was relatively low and did not progress substantially in the placebo group. This finding is consistent with more recent literature on hypertension^26,27^ and is likely explained by the intensification of antihypertensive treatments according to recent guidelines. Indeed, approximately half of the patients in each treatment group were taking an angiotensin-converting enzyme inhibitor, and approximately a third were taking an angiotensin receptor blocker; both medication classes have known antihypertrophic effects. The follow-up duration may also have been too short to reveal an effect of mirabegron in addition to standard treatment. Notably, cardiac β3ARs were shown to be most abundant in advanced cardiac disease,^10,28^ which reciprocally would leave fewer targets for mirabegron in our relatively healthy patient population, particularly with the standard therapeutic dosage of 50 mg/d.

The mirabegron dosage used here (50 mg/d) was shown previously to produce white adipocyte “beiging” and improvements in metabolic parameters, including insulin sensitivity in a patient population with obesity that was comparable to that of Beta3-LVH.^29,30^ Such metabolic effects would be expected to be protective against myocardial remodeling,^10^ albeit in a time frame possibly longer than the present trial. Contrary to previous smaller pilot studies that were not placebo controlled,^30,31^ our results do not confirm any statistically significant effect of mirabegron vs placebo on lipids, glycemic control, or insulin sensitivity (assessed with HOMA). Although the number of participants was smaller, the results of the substudy testing the effect of mirabegron on the abundance or activity of beige or brown fat (by fluorodeoxyglucose–positron emission tomography) at 12 months were also neutral. Likewise, digital microtonometry did not identify any difference from placebo in endothelial function. Nevertheless, such effects may be more apparent with recently developed, more potent β3AR agonists.^32^ Likewise, higher doses of mirabegron associated with β1AR blockers to prevent off-target effects may be useful for patients with more advanced heart failure to stabilize their hemodynamic state before more intensive interventions are implemented, as suggested from the BEAT-HF trials.^21,22^ Longer-term benefits could be expected from combined effects on LV function and remodeling through activation of the upregulated β3AR in these patients, without prohibitive hypotension (unlike direct activators of guanylyl cyclase or other vasodilators). This approach as an add-on to contemporary standard therapy remains to be tested in larger trials.

To our knowledge, Beta3-LVH is the first placebo-controlled randomized clinical trial to assess AE incidence in a patient population with a more severe cardiovascular risk profile (ie, with hypertension, obesity, and structural heart disease and type 2 diabetes or atrial fibrillation) than in previous phase 3 or 4 trials of mirabegron for overactive bladder disease.^33^ In this study, particular attention was given to cardiovascular effects, such as arrhythmias and mild or severe hypertensive episodes. Indeed, preclinical evidence pointed to potential adverse effects through kidney sodium retention,^34^ although the effect of β_3_-adrenergic stimulation on heart rhythm or conduction is still unclear.^35^ Our results showed that at the standard mirabegron dosage (50 mg/d) in addition to multidrug treatment for cardiovascular disease, the incidence of AEs (including mild or severe hypertension by 24-hour ambulatory blood pressure monitoring) did not differ between mirabegron- and placebo-treated patients over 1 year of treatment. Because mirabegron is metabolized through CYP3A4 (and to a lesser extent by CYP2D6), drugs known to interfere with this metabolism were excluded per protocol, leaving a caveat for their concomitant use.

### Limitations

Our inclusion of patients with mild HF and the use of a single standard mirabegron dosage (50 mg/d) may have precluded detection of a treatment effect. Techniques more advanced than measurements of E/e′, such as cardiac strain,^36^ could have been more powerful in assessing early changes in diastolic function. Although they were relatively infrequent and compensated for by appropriate statistical treatment, missing data and dropouts remain limitations.

## Conclusions

In the Beta3-LVH randomized clinical trial, the standard therapeutic mirabegron dosage (50 mg/d) did not result in a difference in LV mass or diastolic function over 12-month follow-up in patients who had structural heart disease but no or mild HF symptoms and were receiving standard therapy. Unlike smaller trials using higher dosages for patients with severe illness,^21,22^ mirabegron had no effect on LV function, RV function, or exercise capacity. In contrast with open pilot studies^29,30^ with the same lower dose, there were also no beneficial effects of mirabegron on lipid or glycemic control. Longer-term effects of β_3_-adrenergic stimulation on myocardial remodeling and function need to be tested in patients with established HFpEF, including with recent, more potent agonists.^32^

## Notes

Supplement 1.
Trial Protocol and Statistical Analysis Plan

Supplement 2.
eMethods

eResults

eReference
**eTable 1.** Participant Inclusion and Exclusion Criteria **eTable 2.** Compliance With Study Medication **eTable 3.** Model-Estimated Means of Left Ventricular Mass Index (LVMI) and E/e′ Over Time in the Mirabegron and Placebo Groups **eTable 4.** Model-Estimated Treatment Effect on Left Ventricular Mass Index (LVMI) and E/e′ (Difference vs Placebo) **eTable 5.** Adverse Events of Special Interest **eTable 6.** Twenty-Four–Hour Ambulatory Blood Pressure Monitoring **eFigure 1.** Trial Flow Chart and Schedule of Assessments **eFigure 2.** Change in Primary Outcomes Over Time in the Mirabegron and Placebo Groups: Per-Protocol Analysis **eFigure 3.** Comparative Effect of Mirabegron and Placebo on the Main Prespecified Secondary Outcomes **eFigure 4.** Comparative Effect of Mirabegron and Placebo on Other End Points **eFigure 5.** Substudy on Endothelial Function

Supplement 3.
Data Sharing Statement
