# Supplementary material for: Repurposing the β3-Adrenergic Receptor Agonist Mirabegron in Patients With Structural Cardiac Disease: The Beta3-LVH Phase 2b Randomized Clinical Trial
Source: JAMA Cardiol. 2023 Sep 20;8(11):1031–40. doi: 10.1001/jamacardio.2023.3003 (PMC10512168; doi:10.1001/jamacardio.2023.3003)
Supplement: Supplement 1. — Trial Protocol and Statistical Analysis Plan [file jamacardiol-e233003-s001.pdf]

Trial Protocol

A multi-centre randomized, placebo-controlled trial of mirabegron, a new beta3-adrenergic receptor agonist on the progression of left ventricular mass and diastolic function in patients with structural heart disease

BETA3_LVH

Coordinating investigator

Prof. Jean-Luc Balligand

Full professor, Head of Department

Pharmacology and Therapeutics (FATH)

Avenue Mounier, 52, B1.53.09

1200 Brussels, Belgium

Phone: +32-2-7645260/+32-2-7645262

Fax: +32-2-7645269

Biometry

Dr. Oana Brosteanu, Dr. Dirk Hasenclever

Clinical Trial Centre Leipzig

Universität Leipzig
Härtelstraße 16-18,

04107 Leipzig, Germany

Phone: +49-341-97 16 250

Fax: +49-341-97 16 189

Sponsor

UNIVERSITÉ CATHOLIQUE DE LOUVAIN

PLACE DE L UNIVERSITE 1, 1348 LOUVAIN LA NEUVE, Belgium

Date: October 21st, 2019

Version: Final 7.0

EudraCT-Nr.: 2015-003146-75

Clinical Trials.gov identifier: NCT02599480

ISRCTN-Number: ISRCTN65055502

The information provided in this document is confidential and is not to be circulated to third parties.

© Version 08 valid as of 01 January 2013

Print: [xxx]

Written by/authors:

Based on the protocol template from the Zentrum für Klinische Studien Leipzig - KKS.

Table of Contents

PAGE

GENERAL INFORMATION 6

Responsible Parties 6
Synopsis 10
Schedule of Assessments and Procedures 15

1 RATIONALE 17

1.1 Medical Background 17
1.2 Rationale 17
1.3 Risk-Benefit Considerations 19

2 OBJECTIVES 21

2.1 Primary Objective 21
2.2 Secondary Objectives 21

3 TRIAL DESIGN AND DESCRIPTION 21

3.1 Trial Design 21
3.2 Requirements at the Trial Sites regarding Personnel and Equipment 21
3.3 Trial Sites and Number of Trial Subjects 24
3.4 Expected Duration of Trial 24
3.5 Premature Termination of the Trial 24

4 TRIAL SUBJECTS 25

4.1 Inclusion Criteria 25
4.2 Exclusion Criteria 25
4.3 Justification for the Inclusion of vulnerable Populations 27
4.4 Participation in more than one Clinical Trial 28
4.5 Statement on the Inclusion of Dependent Individuals 28
4.6 Rationale for Gender Distribution 28

5 INVESTIGATIONAL PRODUCT 29

5.1 Trial Drugs 29
5.2 Packaging and Labelling of the Trial Drug 30
5.3 Drug Accountability 31
5.4 Administration of the Study Drug 32
5.5 Blinding and Unblinding 36

6 INDIVIDUAL TRIAL PROCEDURES 36

6.1 Patient Information and Informed Consent 36
6.2 Enrolment in the Trial 38
6.3 Description of the Treatment Procedures 38
6.4 Premature Termination of the Therapy for Individual Patients 41
6.5 Premature study termination for individual patients 41
6.6 Plan for Further Treatment 42

7 METHODS OF DIAGNOSTICS AND DATA SAMPLING 42

8 ADVERSE EVENTS (AE/SAE) 42

8.1 Adverse Events (AE) 42
8.2 Safety Analysis 43
8.3 Concomitant Diseases 43
8.4 Serious Adverse Events (SAE) 43

8.5 Periodic Reports 45
8.6 Suspected Unexpected Serious Adverse Reactions (SUSAR) 45
8.7 Other Safety Relevant Issues 46
8.8 Therapeutic Procedures. 46
8.9 Dealing with Pregnancy 46

9 BIOMETRY 48

9.1 Biometrical Aspects of the Trial Design 48
9.2 End Points 48
9.3 Statistical Description of the trial hypothesis 49
9.4 Sample Size Discussion 49
9.5 Statistical Methods 50
9.6 Statistical Monitoring 52
9.7 Interim Analysis 53
9.8 Final Analysis 53

10 CONCOMITANT SCIENTIFIC PROJECTS 53

10.1 Endothelial Function/Pulse amplitude tonometry and measurement of HbNO 53
10.2 Abundance/activity of brown/beige fat 54
10.3 Analysis of blood and urine samples for scientific purposes related to cardiac disease 54

11 ETHICAL, LEGAL AND ADMINISTRATIVE ASPECTS 55

11.1 GCP-Statement 55
11.2 Initial Submission 55
11.3 Protocol Amendments 55

12 DOCUMENTATION 56

12.1 Case Report Forms (CRF) 56
12.2 Data Management 57
12.3 Archiving 57

13 SUPERVISION OF THE CLINICAL TRIAL 58

13.1 Access to Source Data 58
13.2 Monitoring 58
13.3 Audits 59
13.4 Inspections 59
13.5 Independent Supervision of the Trial 59

14 DATA PROTECTION AND CONFIDENTIALITY 60

14.1 Declaration regarding Data Protection 62
14.2 Declaration regarding the Pseudonymized Transfer of Personal Data 62

15 ADMINISTRATIVE AGREEMENTS 62

15.1 Adherence to the Protocol 62
15.2 Funding and Insurance 62
15.3 Notification of the Local Authorities (Germany only) 63
15.4 Publication Policy and Registration 63

16 REFERENCES 64

17 PROTOCOL SIGNATURES 72

18 PROTOCOL AGREEMENT 73

19 APPENDIX 74

19.1 Classification of Adverse Events 74

19.2 Definitions 75
19.3 Acronyms 76
19.4 Labelling 77

List of Tables

table 1: Schedule of Assessments and Procedures 15

List of Figures

Figure 1: Flowchart of the trial 16
Figure 2: Sample of blister-label 30
Figure 3: Sample of box-label 31

GENERAL INFORMATION

Responsible Parties

Sponsor<br>*(according to Belgian drug law)*	Université catholique de Louvain (UCL)<br>Place de l'Université 1<br>1348 Louvain la Neuve<br>Belgium<br>Authorised representative of the sponsor, Promotor:<br>Prof. Jean-Luc Balligand
Coordinating investigator(s)	Prof. Dr. Jean-Luc Balligand<br>Full professor, Head of Department<br>Pharmacology and Therapeutics (FATH)<br>Avenue Mounier, 52, B1.53.09<br>1200 Brussels, Belgium<br>Phone: +32-2-7645260/+32-2-7645262<br>Fax: +32-2-7645269<br>E-Mail: jean-luc.balligand@uclouvain.be
Deputy of Coordinating investigator	Anne-Catherine Pouleur<br>Cliniques universitaires Saint-Luc<br>Avenue Hippocrate, 10<br>1200 Bruxelles, Belgique<br>Phone: +32-2-764 28 12,<br>Fax: +32-2-764 89 80<br>E-Mail: anne-catherine.pouleur@uclouvain.be
Project Management	Dr Ir Nancy van Overstraeten<br>UCL-FATH<br>Institut de Recherche Expérimentale et Clinique (IREC)<br>Building Vésale 5th floor ; 52 Avenue Mounier<br>B1.53.09; 1200 Brussels; Belgium<br>Phone: +32-2-764 52 88<br>Fax: +32-2-764 5269<br>E-Mail: nancy.vanoverstraeten@uclouvain.be<br><br>Dr. Petra Neuhaus<br>Zentrum für Klinische Studien Leipzig – KKS<br>Universität Leipzig<br>Härtelstraße 16-18, 04107 Leipzig, Germany<br>Phone: +49 341 97 16255<br>Fax: +49 341 97 16 16189<br>E-Mail: petra.neuhaus@zks.uni-leipzig.de
Pharmacovigilance	Madlen Dörschmann<br>Zentrum für Klinische Studien Leipzig – KKS<br>Universität Leipzig

	Härtelstraße 16-18, 04107 Leipzig, Germany<br>Phone: +49 341 97 16129<br>Fax: +49 341 97 16 16278<br>E-Mail: madlen.doerschmann@zks.uni-leipzig.de
Data Management	Angelika Beyer<br>Zentrum für Klinische Studien Leipzig – KKS<br>Universität Leipzig<br>Härtelstraße 16-18, 04107 Leipzig, Germany<br>Phone: +49 341 97 16261<br>Fax: +49 341 97 16259<br>E-Mail: angelika.beyer@zks.uni-leipzig.de
Biometry	Dr. Oana Brosteanu<br>Zentrum für Klinische Studien Leipzig – KKS<br>Universität Leipzig<br>Härtelstraße 16-18, 04107 Leipzig, Germany<br>Phone: +49 341 97 16251<br>Fax: +49 341 97 16189<br>E-Mail: oana.brosteanu@zks.uni-leipzig.de<br><br>Dr. Dirk Hasenclever<br>Institut für Medizinische Informatik, Statistik und Epidemiologie<br>Universität Leipzig<br>Härtelstraße 16-18, 04107 Leipzig, Germany<br>Phone: +49 341 97 16121<br>Fax: +49 341 97 16109<br>E-Mail: dirk.hasenclever@imise.uni-leipzig.de
Monitoring	Nicole Köppe-Bauernfeind<br>Zentrum für Klinische Studien Leipzig – KKS<br>Universität Leipzig<br>Härtelstraße 16-18, 04107 Leipzig, Germany<br>Phone: +49 341 97 16266<br>Fax: +49 341 97 16259<br>E-Mail: nicole.koeppe-bauernfeind@zks.uni-leipzig.de
Funding	EU Framework Programme for Research and Innovation, Horizon 2020:<br>Mr. Grzegorz OWSIANIK<br>Marie-Christine Groenen<br>Medical research and the challenge of ageing<br>European Commission<br>DIRECTORATE-GENERAL FOR RESEARCH & INNOVATION<br>Medical research and the challenge of ageing<br>Directorate Health

	B-1049 Brussels Belgium<br><br>Phone: +32 22951192<br>E-Mail: Grzegorz.OWSIANIK@ec.europa.eu<br>Marie-Christine.Groenen@ec.europa.eu
Data Safety and Monitoring Board	*Organisation:*<br>Prof. Dr. Stefan Anker<br>Charité - Universitätsmedizin Berlin<br>Campus Virchow-Klinikum<br>Augustenburger Platz 1,<br>13353 Berlin, Germany<br>Phone: +49 (0) 30-450 553463<br>Fax: +49 (0) 30-450 553951<br>E-Mail: s.anker@cachexia.de<br><br>*Chair:*<br>Karl Swedberg, Prof. MD<br>Department of Molecular and Clinical Medicine, Sahlgrenska Academy, University of Gothenburg, Sweden, and National Heart and Lung Institute, Imperial College, London, UK<br>E-mail: karl.swedberg@gu.se<br><br>*Members:*<br>Dan Atar, MD, FESC, FACC, FAHA<br>Professor of Cardiology<br>Head of Research, Div. of Medicine<br>Oslo University Hospital, Ullevål<br>Kirkeveien 166, Building 3-A<br>N - 0407 Oslo - Norway<br>Ph: + 47 22 11 91 00<br>Email W: dan.atar@ous-hf.no<br>Email H: dan.atar@online.no<br><br>Luigi Tavazzi, MD, FESC, FACC<br>GVM Care and Research,<br>E.S. Health Science Foundation, Maria Cecilia Hospital<br>Via Corriera, 1<br>48010 Cotignola (RA) - Emilia-Romagna<br>Italy<br>E-mail: 'Elisa Ghizzardi GVM Care&Research' <direzionescientifica-mch@gvmnet.it><br><br>Hans Wedel<br>KB Biostatistics<br>Apelsingatan 115

	426 54 Västra Frölunda<br>Phone: 070-829 72 88<br>E-mail : hans.wedel@biostat.se
Concomitant scientific projects	Endothelial Function/Pulse amplitude tonometry and measurement of HbNO:<br>Dr Ignatios Ikonomidis,MD,PhD,FESC<br>Assistant Professor, Member of EACVI<br>Nucleus member of ESC WG Peripheral Circulation<br>2nd Cardiology Department<br>University of Athens<br>Attikon Hospital<br>Rimini 1, 12462<br>Haidari, Greece<br>E-mail: ignoik@otenet.gr,ignoik@gmail.com<br>tel +30 6944805732<br>fax +30 210 5832351 ,210 5832192<br><br>Abundance/activity of brown/beige fat:<br>Dr Renaud Lhommel (CUSL)<br>Cliniques universitaires Saint-Luc<br>Avenue Hippocrate, 55 bte B1.55.01<br>1200 Bruxelles<br>Belgique<br>Phone: +32 2 764 25 82<br>Fax: +32 2 764 16 02<br>E-Mail: renaud.lhommel@uclouvain.be

Synopsis

Title of the trial	A multi-centre randomized, placebo-controlled trial of mirabegron, a new beta3-adrenergic receptor agonist on the progression of left ventricular mass and diastolic function in patients with structural heart disease.
Acronym	BETA3_LVH
Indication	Structural heart disease at high risk for progressive hypertrophic cardiac remodeling at risk of developing HFpEF (heart failure with preserved ejection fraction).
Primary goal of the trial / primary end point	The primary objective is to evaluate the effect of mirabegron (a new β3-specific agonist) on change in left ventricular mass and/or changes in diastolic function after 12 months of treatment in patients with cardiac structural remodeling with or without symptoms of heart failure (maximum NYHA II).<br><br>Two equally ranked, primary endpoints:<br>• Change in left ventricular mass index (LVMI in g/m^2, defined as left ventricular mass divided by body surface) measured at baseline and 12 months after randomisation.<br>• Change in diastolic function, assessed as the ratio of peak early transmitral ventricular filling velocity to early diastolic tissue Doppler velocity (E/e') measured at baseline and 12 months after randomisation.
Secondary goals of the trial / secondary end points	Effect of mirabegron on other indicators for diastolic heart disease, i.e. cardiac fibrosis, left atrial volume index, diastolic function (E/e'), maximal exercise capacity and laboratory markers (analysed after 6 or 12 months of mirabegron treatment).<br><br>Secondary endpoints:<br>• Further MRI endpoints (all measured in the central MRI core lab)<br>- Cardiac fibrosis at baseline and at 12 months. Fibrosis is a key pathogenic mechanism of diastolic dysfunction, which is at the origin of HFpEF<br>- Left atrial volume index at baseline and at 12 months. This parameter determines diastolic filling (and was shown to predict treatment efficacy in HFpEF in the J-DHF trial (Yamamoto et al. 2013))<br>- LV mass index (by cardiac MRI) at 6 months,<br>- Diastolic function (E/e') at 6 months;<br>• Laboratory parameters at baseline and at 3, 6 and 12 months<br>- serum biomarkers (Galectin3, GDF15, NT-proBNP, hsTnT)<br>- metabolic parameters (fasting glucose, modified HOMA test, HbA1c, serum lipids)<br>• Maximal exercise capacity (peak VO2) at baselineand 12 months.

	• Safety endpoints<br>- Incidence, severity and frequency of adverse and serious adverse events<br>- Mortality
Trial design	Two armed, prospective, randomized, placebo-controlled, multi-centric international phase IIb trial
Trial population	Inclusion criteria:<br>• Age between 18 and 90 years<br>• Morphological signs of structural cardiac remodelling by echocardiography, i.e. increased LV mass index (≥95 g/m^2 or higher for female; ≥115 g/m^2 or higher for male subjects (Ponikowski et al. 2016)) or end-diastolic wall thickness ≥13 mm in at least one wall segment<br>• Written informed consent<br>For subjects unable to read and/or write, oral informed consent observed by an independent witness is acceptable if the subject has fully understood oral information given by the Investigator. The witness should sign the consent form on behalf of the subject.<br><br>Major exclusion criteria:<br>• Unstable arterial hypertension with systolic BP≥160 mm Hg and/or diastolic BP≥100 mm Hg (confirmed at three consecutive office measurements in sitting position); if so, the patient may be re-screened after optimization of anti-hypertensive treatment<br>• Hypertensive patients not under stable therapy according to current guideline algorithm (Mancia et al. 2013) (including stable medication for at least 4 weeks before inclusion)<br>• Documented ischemic cardiac disease:<br>- current angina pectoris or<br>- ischemia on stress test or<br>- untreated coronary stenosis >50% or<br>- history of acute myocardial infarction (AMI) or<br>- coronary artery bypass graft (CABG, < than 3 months prior to screening) or<br>- percutaneous transluminal coronary angioplasty (PTCA) less than 3 months prior to screening.<br>• Patients with uncontrolled recurrent persistent and permanent atrial fibrillation (AF) according to AHA/ACC/ESC guidelines (Dixon et al. 2005) (with a heart rate > 100/min, RACE II - (Groenveld et al. 2013). If AF with HR>100/min, the patient may be rescreened after treatment for rate control.<br>• History of hospitalization for overt heart failure within last 12 months<br>• History of high degree impulse conduction blocks (> 2nd degree AV block type 2)<br>• Patients after heart transplantation<br>• Genetic hypertrophic or dilated cardiomyopathy<br>• Dysthyroidism

	• Severe valvulopathy (less than 1 cm2 aortic valve area, mitral insufficiency of severe grade at Doppler echo)<br>• Congenital valvulopathies<br>• Patients with a known history of QT prolongation (QT>450ms) or patients with documented QT prolongation (QT>450 ms) while taking medicinal products known to prolong the QT interval.<br>• NYHA-class > II<br>• BMI > 40 kg/m^2<br>• EF < 50%, regardless of symptoms<br>• Known other cause (i.e. COPD) of respiratory dysfunction; patients under positive pressure (CPAP) treatment for sleep apnea syndrome may be included, provided they have been efficiently controlled by CPAP for at least one year before inclusion in the study<br>• Moderate renal impairment defined as eGFR < 30 ml/min<br>• Abnormal liver function tests (AST or ALT >2 X upper normal limit or patients with known hepatic impairment defined as Child-Pugh class B or higher)<br>• Type I diabetes, complicated type II diabetes (i.e. with documented coronary macroangiopathy , cfr exclusion criterion 1 or documented other vascular complication) (National Diabetes Education Initiative - NDEI).<br>• Patients with anemia (male: Hb <130 g/l, female: Hb <120 g/l)<br>• Patients with bladder outlet obstruction<br>• Patients using antimuscarinic cholinergic drugs for treatment of OAB<br>• Current use of digitalis, bupranolol, propranolol, nebivolol (known to interfere with β3AR signalling)<br>• Note: patients are allowed to take a β(1-2)-blocker, other than the drugs listed above (for explanation, see chapter 5.4.5).<br>• Patients continuously treated with Sildenafil or other PDE5 inhibitors.<br>• Current use of antifungal azole derivatives (fluconazole, itraconazole, miconazole, posaconazole, voriconazole) (known inhibitors of CYP3A4, the main metabolizer of mirabegron)<br>• Current treatment with mirabegron or indication for future treatment with mirabegron due to other indications<br>• Contraindication for MRI (e.g.defibrillator, ferromagnetic devices or severe claustrophobia, pacemaker - the latter only, if MRI is contraindicated)<br>• Pregnant or nursing women<br>• Women of child bearing potential without highly effective contraceptive measures (Clinical Trial Facilitation Group (CTFG) 9/15/2014):<br>- combined (estrogen and progestogen containing) hormonal contraception associated with inhibition of ovulation (oral, intravaginal, transdermal)<br>- progestogen-only hormonal contraception associated with inhibition of ovulation (oral, injectable, implantable)<br>- intrauterine device (IUD)<br>- intrauterine hormone-releasing system (IUS)

	- bilateral tubal occlusion<br>- vasectomised partner<br>- sexual abstinence (only if in agreement with the preferred and usual lifestyle of the subject)<br>while participating in the trial. There are no known interactions of the trial medication and hormone-based methods of contraception. In particular, no clinically relevant interactions have been observed when mirabegron was co-administered with therapeutic doses of a combined oral contraceptive medicinal product containing ethinylestradiol and levonorgestrel.<br>• Participation in any other interventional trial<br>• Patients unable to give informed consent (people under legal guardianship)<br>• Patients placed in an institution by official or court order<br>• Contraindication to mirabegron (e.g. hypersensitivity) or any other components of the trial medication
Sample size	296 patients overall<br>(148 patients per treatment arm) will be randomised
Therapy	Experimental group:<br>Administration of 50 mg mirabegron once daily per os (Betmiga®, Astellas Pharma) over a period of 12 months.<br><br>Control group:<br>Administration of placebo once daily per os over a period of 12 months.<br><br>Verum and placebo will be manufactured by:<br>Pharmacy of the University Hospital Leipzig<br>Liebigstr. 20<br>D-04103 Leipzig
Biometry	Primary endpoints:<br>Confirmatory analysis follows the intention to treat principle as close as possible and will be based on the full analysis set.<br>In each patient, the primary endpoints will be assessed thrice: at the baseline visit, and at the 6 months and 12 months visits. Analyses of both primary endpoints are identically structured:<br><br>Mean changes from baseline mean will be analysed using a repeated measurement linear mixed model without intercept containing the fixed, categorical effects of<br>• visit (baseline, 6 months, 12 months),<br>• treatment (verum / placebo),<br>• treatment by visit interaction<br>• Atrial fibrillation (yes / no),<br>• Diabetes mellitus (yes / no),<br>as well as a patient-specific, visit random effect (3-dimensional normal with a general unstructured variance covariance matrix).<br><br>Secondary endpoints:

	All other MRI and echocardiographic endpoints as well as peak VO2 will be analysed along the same lines as the primary endpoints. Time courses of metabolic parameters and specific biomarkers will be described.<br>Adverse and serious adverse events will be compared by chi-square tests. Odds ratios with 95% confidence intervals will be provided.
Trial Duration	Individual treatment period:<br>12 months treatment per patient with an additional follow-up-phone visit at month 13<br><br>Trial:<br>• First patient in: April 2016<br>• Patient-screening and recruitment: 51 months<br>• Last patient out (planned): June 2021<br>• Final analysis (planned) December 2021

Schedule of Assessments and Procedures

Visit<br>month[a]	Screening<br>-1 to 0	Baseline<br>0	V1<br>1[b]	V2<br>3[c]	V3<br>6[c]	V4<br>9[c]	V5<br>12[c]	V6<br>13[d]
Eligibility criteria	●	●						
Informed consent	●							
Medical history/current medication	●	●	●	●	●	●	●	●
Concomitant diseases	●	●						
Registration	●							
Randomisation		●						
Clinical assessment								
BP measurement[3]	●	●	●	●	●	●	●	
24 h ABPM		●					●	
NYHA functional class & symptoms, if any	●	●	●	●	●	●	●	
Body Mass Index (BMI)	●	●	●	●	●	●	●	
Heart Rate (HR)	●	●	●	●	●	●	●	
Pregnancy test (if applicable)	●	●	●	●	●	●	●	
ECG	●							
Echocardiography								
standard[4]	●							
extended[5]		●			●		●	
Cardiac MRI[6]		●			●		●	
Maximal exercise capacity		●					●	
Local laboratory tests								
Hemogram (incl. HCT)		●			●		●	
Liver function test[7]	●		●	●	●	(●)	●	
Renal function test[8]	●		●	●	●		●	
Thyroid stimulating hormone	●							
Central laboratory tests								
Metabolic parameters[9]		●		●	●		●	
Specific biomarkers[10]		●		●	●		●	
Blinded study medication		●	●	●	●	●		
Drug accountability		●	●	●	●	●	●	
Adverse events		●	●	●	●	●	●	●
Blood-sampling for later scientific sub-projects		●	●		●	●	●	
Urine-sampling for later scientific sub-projects		●	●		●	●	●	
Sub-study								
Endothelial function		●			●		●	
Blood-sampling central lab (Hb-NO)		●			●		●	
^{18}FDG-PET		●					●	

table 1: Schedule of Assessments and Procedures

a whereby one month is defined as 28 days
b ± 2 days
c ± 14 days
d ± 2 days, Follow-up by phone
3 3x in sitting position at each visit
4 LV mass, wall thickness and function
5 standard & Doppler mitral flow, tissue Doppler imaging, E/E'
2x echocardiographic assessment at each time point → to be analysed in the core lab
6 MRI to be analysed in the core lab
7 AST, ALT, GGT, at baseline visit and visits 4 only if suspicion of liver disease
8 eGFR estimated by MDRD formula; additionally at visit 4, if clinical symptoms of renal dysfunction are observed
9 fasting glucose, HOMA test, HbA1c, serum lipids
10 Galectin3, GDF15, NT-proBNP, hsTnT

Flow Chart

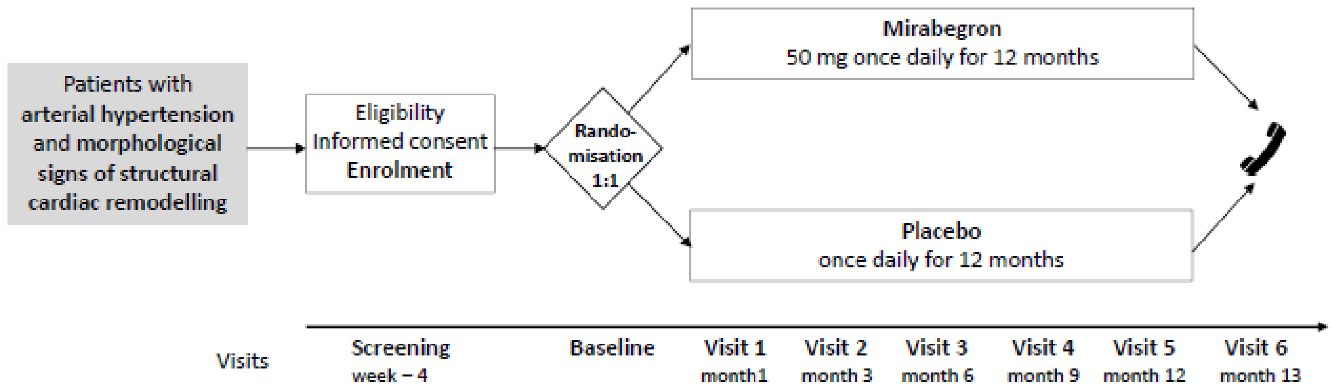

Figure 1: Flowchart of the trial

1 RATIONALE

1.1 Medical Background

Heart Failure: a chronic non-communicable disease that burdens patients and health care systems:

Heart failure (HF) represents a major and growing public health burden, affecting 2% - 3% of adults in developed countries. Importantly, HF is a progressive disorder. Its prevalence increases steeply with age, from <1% in the 20- to 39-year age group to >20% in individuals aged >80 (Lloyd-Jones 2010). Patients with HF are classically divided into two groups: those with HF with preserved ejection fraction (HFpEF), and those with HF and reduced ejection fraction (HFrEF). In the last two decades, the proportion of patients with HFpEF has increased from 38% to 54% of cases of HF, a proportion that will continue to rise at an alarming rate of around 1% per year due to the progressive aging of the population. In the USA, the estimated annual cost of HF in 2010 is estimated to be ≈2% of the total US health-care budget (Lloyd-Jones 2010). Evaluations from different European countries indicate a similar share of HF-related costs in relation to overall health-care expenditure (National Clinic Guideline Center 2010). From an individual perspective, the diagnosis of HF is associated with annual costs of ≈$8.500 per patient according to data from the National Heart and Lung Institute cardiovascular health study. These estimates may greatly undervalue the real costs as they are based on data with HF as the primary diagnosis while costs for HF treatment may also occur in many patients primarily hospitalised for one of the multiple comorbidities that typically accompany HF such as hypertension, diabetes, and renal or lung disease. As a consequence of demographic trends and a broad expansion of the treatment armamentarium, the annual costs for HF have been steadily increasing. In the USA, for example, the estimated annual cost for HF rose from $24.3 billion in 2003 to 39.2 billion in 2010; based on the proportion as stated above, it can be estimated that about half of this cost is due to HFpEF. Beside costs, HFpEF also puts a heavy burden on the quality of life of (mostly elderly) patients, with a loss of autonomy and the dyscomfort of repeated hospitalisations. Therefore, HFpEF is a chronic, costly, debilitating disease.

Compared to HFrEF, patients with HFpEF are older, often female, and have a high prevalence of hypertension, diabetes and obesity, as well as various comorbidities such as atrial fibrillation, renal failure and anemia (Redfield et al. 2003). A major contributor to HFpEF is myocardial remodelling, e.g. hypertrophy and fibrosis, as well as cellular functional/structural modifications leading to alteration in contractile (including relaxation) properties and ventricular distensibility. Unfortunately, despite the growing incidence of HFpEF over the last 15 years, there currently are no evidence-based treatment strategies that will change its evolution (McMurray, John J V et al. 2012). This puts more emphasis on new strategies and targets that may prevent the progression of remodelling towards the development of LV dysfunction and symptomatic HFpEF.

1.2 Rationale

1.2.1 Hypothesis and Experimental Aspects of the Clinical Trial

The proposed clinical trial will support proof of concept in humans to assess the clinical efficacy of a novel therapeutic concept: β_3AR activation to attenuate/prevent cardiac remodeling. It will contribute to optimisation of available therapies, by extending the potential indication of a drug (mirabegron) currently used for non-cardiovascular disease (“drug repurposing”), to a highly prevalent disease affecting mostly elderly patients. As many of these are also susceptible to suffer from overactive bladder disease (the current indication for mirabegron), the trial will contribute valuable information on additional benefit for those suffering from cardiovascular diseases; conversely, in case of adverse effects, it may contribute to early exclusion of an inappropriate therapeutic strategy in such patients.

Recently, a new, specific agonist at human β_3-AR (mirabegron) was developed and marketed for clinical use in a non-cardiovascular disease (overactive bladder disease) (Chapple et al. 2014). Compared with previous preferential agonists, it has superior specificity with lower incidence of side-effects, particularly on the cardiovascular system (cfr European Medicines Agency mirabegron EPAR report EMA/706651/2012; see also safety considerations below). We and others identified the expression of the third isotype of β-adrenergic receptor (β_3AR) in human cardiac and vascular tissues (Moniotte et al. 2001; Gauthier et al. 1996; Gauthier et al. 1998). Using pre-clinical models, we demonstrated that activation of β_3AR attenuates myocardial hypertrophy and fibrosis in response to neurohormonal or hemodynamic stresses, without compromising LV function (Belge et al. 2014). Therefore, the recent availability of this new drug offers the possibility to test the potential benefit of mirabegron (vs placebo) as add-on therapy (on top of standard care) to prevent/delay myocardial remodelling in patients at high risk of developing HFpEF. The concept behind this proposition is not merely to extend the use of mirabegron in the cardiovascular therapeutic area, but to propose a novel treatment modality for a disease where all other strategies have failed over the last decade. Our (and others') preclinical work provides strong evidence in favour of a likely beneficial effect through pleiotropic targeting of both LV remodelling and co-morbidities, whereas the clinical database leading to the development of mirabegron in the urological field did not identify alarming side-effects that would preclude its utilisation in patients with cardiovascular disease. Therefore, the use of mirabegron in HFpEF may potentially yield far-reaching benefits (both economical and in terms of quality of life) at little extra costs of development and little anticipated iatrogenic costs (due to side-effects). This makes it a cost-effective new treatment for HFpEF.

The underlying concept is built on pre-existing pre-clinical research generated by members of this consortium on the effect of β_3AR on cardiac remodelling. Indeed, after the definitive identification of human β_3AR in human myocardial cells (both cardiac myocytes and endothelial cells) (Gauthier et al. 1996, 1996; Gauthier et al. 1998; Dessy et al. 2004), we described the coupling of this receptor to the nitric oxide/cyclicGMP pathway, that resulted in an effect on contractility that is antipathetic to classical $\beta_{1\text{-}2}$AR positive inotropic effects (Gauthier et al. 1998). As ample evidence now points to the adverse effects of sustained activation of $\beta_{1\text{-}2}$AR, leading to receptor desensitisation/ internalisation, loss of BETA3_LVH (Lloyd-Jones 2010) contractile/frequency reserve, adverse remodelling, calcium overload and myocyte loss, we reasoned that activation of the functionally antipathetic β_3AR would protect against such deleterious effects of chronic adrenergic stimulation. To test this hypothesis, we used a transgenic mouse model with cardiac myocyte-specific expression of the human β_3AR, and submitted these mice (and their littermate controls) to a number of interventions all leading to myocardial stress (i.e. mini-pump or i.p. infusions of isoproterenol or angiotensin II, transaortic constriction). The results uniformly showed protection of the transgenic mice from the development of pathologic remodelling contrary to the WT controls (Belge et al. 2014). Importantly, this was not at the expense of LV function, which remained normal. The β_3AR may then be an attractive target to prevent adverse remodelling in the face of chronic adrenergic stimulation, all the more because it is distinctively resistant to homologous desensitisation (rodent and human β_3ARs lack consensus sequences for phosphorylation by βARK or PKA) and retains coupling to downstream signalling in the pathologic heart, as demonstrated by us in human diseased myocardium *ex vivo* (Moniotte et al. 2001). Moreover, contrary to $\beta_{1\text{-}2}$AR, its expression increases in the diseased myocardium (Moniotte et al. 2001). On the basis of our observations in transgenic mice, one can assume that this β_3AR upregulation is a protective mechanism in the face of myocardial stress. However, as the β_3AR is typically activated by higher catecholamines concentrations (than $\beta_{1/2}$AR), it is possible that this protective pathway is not maximally recruited even in circumstances of pathophysiologic adrenergic activation. This would leave a therapeutic margin for an additional activation by a potent and specific β3AR agonist, such as mirabegron.

1.3 Risk-Benefit Considerations

Based on older evidence (Gauthier et al. 1996), there were concerns that the therapeutic potential of β3AR agonists would be limited by cardio-depressive effects, following the demonstration that, *ex vivo*, cardiac β3AR stimulation was associated with negative inotropy in human ventricular samples. That administration of BRL37344, a β_3AR agonist, in large animal models of HF, does not translate to decreased cardiac performance *in vivo* (Bundgaard et al. 2010), may help to alleviate these concerns. Clinical data from the mirabegron clinical studies in Overactive Bladder Disease also do not raise major concerns. In healthy volunteers mirabegron causes a dose-dependent increase in heart rate (3-6 hrs post-dosing) and in systolic blood pressure (24 hr average), presumably as a result of baroreflex activation secondary to short-term hypotensive effects. In the clinical trial populations, in aggregate, this translated to an increase of approximately 1 bpm in heart rate, and an increase in systolic blood pressure of <1 mm Hg, which was not associated with increased cardiovascular complications (European Medicines Agency mirabegron EPAR report EMA/706651/2012). Although the treatment period was limited to 8-12 weeks in the initial phase II trials, this has been confirmed in safety and tolerability studies with a 1 year treatment duration (Chapple et al. 2014).

Based on pre-clinical to clinical development of mirabegron, the efficacy of mirabegron at the target is shown, paired with good tolerability and safety results:

- Mirabegron has excellent specificity on β_3AR (as opposed to $\beta_{1\text{-}2}$AR), with well documented bioavailability and little potential for adverse effects resulting from drug interactions, at least at our chosen dose of 50 mg/day.

- Mirabegron is metabolised by CYP3A4 and is a (weak) inhibitor of CYP2D6 and P-GP; it is also eliminated by urinary excretion; despite expected changes in drug exposure related to age, renal or hepatic dysfunction, a reduction of the dose to 25 mg/day is only recommended in case of concomitant use of a potent CYP3A4 inhibitor (such as ketoconazole) in patients with liver or renal impairment; consumption of such inhibitors will be listed in the "exclusion criteria", as will be patients with significant renal or hepatic impairment; for P-GP, as interactions with digoxin may be foreseen, use of digoxin will also be listed in BETA3_LVH "exclusion criteria"; women taking mirabegron were also documented to present higher exposure to the drug than men (at similar doses); fortunately, during clinical development in phase 2/3 for Overactive Bladder Disease, 72-83% of subjects were female (a gender predominance that we expect to observe in our cardiovascular trial as well, given the higher prevalence of HFpEF in women), and in this mostly female population the incidence of SAE was not different from placebo.

- Mirabegron showed a good tolerability during phase 2/3 trials; overall, 85,6% of patients treated completed the studies (14,4% discontinued the medication)

- Mirabegron showed a favourable safety profile in phase 2/3 trials, on 5648 patients treated for Overactive Bladder Disease in all trials, 622 received mirabegron 50 mg/d during 1 year or more; among these, the cardiovascular safety profile was excellent, i.e. heart rate changes from baseline compared with placebo was 1 bpm or less in both genders and all age categories; adjusted mean difference in blood pressure versus placebo and adjusted mean change from baseline SBP/DBP was approximately 1 mm Hg or less in both 3 months and long-term studies; the frequency of QTc>450 msec was similar to placebo for all doses of mirabegron <200mg/d, with no effect at 50 mg/d (notably all QT studies included 70% of women, with a median age of 60 years, and 35% above age 65); there was no sign of increased malignancies, as the rate of observed malignancies was similar to the incidence of an age-adjusted population, with no specific neoplastic disease domination (European Medicines Agency mirabegron

EPAR report EMA/706651/2012, and UK NICE reports http://www.nice.org.uk/guidance/ta290).

- However, post-marketing observations identified a risk of acute blood pressure increases with possible acute cardiovascular events in patients with uncontrolled high blood pressure (systolic blood pressure > 180 mm Hg and diastolic blood pressure > 110 mm Hg). Therefore, the EMA has asked the manufacturer of mirabegron to include a warning and a contraindication for the use of this drug in this category of patients. (see communication by Astellas). In reaction to this communication, we exclude patients with uncontrolled high blood pressure (see section 4.2)

Considerations of effectiveness and potential clinical benefit:

- Effectiveness: As mirabegron is currently widely used and well tolerated for the treatment of overactive bladder disease, the same oral administration of mirabegron in HFpEF patients would be easily applicable in real-life medical situations, outside and beyond the clinical trial. In favour of this, as OAB also mostly affects elderly patients, there is already post-marketing evidence of tolerability in a patient population similar to that targeted in HFpEF, many of which probably have the same co-morbidity and/or HFpEF.

- Potential clinical benefit: Based on these and our pre-clinical data, as well as independent studies by others (see below), there is a reasonable likelihood that activation of this new target (β_3AR) will result in measurable attenuation of cardiac remodelling, as assessed by validated surrogate endpoints with state-of-the art techniques (cardiac MRI for ventricular hypertrophy and fibrosis; Doppler echocardiography for diastolic function); our measurements of functional parameters (VO_2 max, 6 min walk-test) will add important information that are known to bear on the quality of life of (mostly) elderly patients with heart failure, which in this population category has more importance than the number of years added (i.e. assessment of mortality, for which our trial will be underpowered).

2 OBJECTIVES

2.1 Primary Objective

The primary objective is to evaluate the effect of mirabegron (a recent β3-specific agonist) on change in left ventricular mass and/or changes in diastolic function after 12 months of treatment in patients with cardiac structural remodeling with or without symptoms of heart failure (maximum NYHA II).

2.2 Secondary Objectives

Besides the primary effect of mirabegron on the above mentioned criteria, its effect on other indicators for diastolic heart disease, i.e. cardiac fibrosis, left atrial volume index, diastolic function (E/e'), maximal exercise capacity and laboratory markers (for details see chapter 9.2.2) will be analysed after 6 or 12 months of mirabegron treatment.
For an operational definition of the study endpoints see chapter 9 (Biometry).

3 TRIAL DESIGN AND DESCRIPTION

3.1 Trial Design

BETA3_LVH is a two-armed, prospective, randomized, placebo-controlled, multi-centric European phase IIb clinical trial. The target population consists of patients with cardiac structural remodelling with or without symptoms of heart failure (maximum NYHA II).

3.2 Requirements at the Trial Sites regarding Personnel and Equipment

The (principal) investigator is licensed to practice medicine, is a medical specialist and has at least two years of work experience in cardiology. He has theoretical and practical experience in conducting clinical trials and proven knowledge of the ICH-GCP guidelines (GCP refreshment courses in case of significant changes).

3.2.1 Qualification of investigator/deputy (if applicable) and medical staff in the study team

All medical personnel involved will be trained in the use of the eCRF by the CTC Leipzig as well as in the requirements on image capturing by the involved core labs in advance of trial start.

3.2.1.1 Belgium

The investigator(s) in Belgium have to fulfil the requirements of GCP (ICH-E6, revised 1996) and the Belgian Law of May 7, 2004 concerning experiments on the human person.

3.2.1.2 France

The investigator is licensed to practice medicine and is registered to "Ordre National des Médecins". He/She has to provide his/her updated, dated and signed curriculum vitae to the Sponsor to evaluate his/her qualifications to conduct clinical trial. The dossier provided to the ethical committee will contain this information. The investigator knows and follows the applicable regulation and good clinical practice.

3.2.1.3 Germany

The investigator and deputy are licensed to practice medicine, are specialists in internal medicine and have at least two years work experience in cardiology. They have theoretical and practical experience in conducting clinical trials. Their qualification is defined as follows:

- Documented proof of at least two years experience in conducting clinical trials after August 2004 (12. Amendment of German Drug Law) incl. proof of GCP training

AND

- Updates of GCP knowledge and revisions of German Drug Law every two to three years, if necessary (significant changes since last training)

The Investigator is responsible for selecting and assembling the study team members (especially the medical staff) according to the requirements of this trial protocol. Furthermore, the investigator is responsible for training and supervision of the study team and providing all necessary information. This has to be documented.

Medical staff is licensed to practice and has at least theoretical experience in conducting clinical trials. The qualification is defined as follows

- Certification of successful participation in an investigator course incl. GCP training

OR

- Documented proof of conducting clinical trials after August 2004 (12. Amendment of German Drug Law) incl. proof of GCP training

AND

- Updates of GCP knowledge and revisions of German Drug Law every two to three years, if necessary (significant changes since last training)

3.2.1.4 Greece

According to the current harmonized legislation and the principles of Good Clinical Practice ICH-GCP (Chapter 4.1.1), each person involved in the conduct of a clinical study must have the necessary qualifications to perform the corresponding duties, in terms of education, training and experience in the clinical trials.

Therefore, the below listed points should be documented in Principal Investigator's and sub-investigators' CVs:

- the recent theoretical and practical experience of the involved person in conducting clinical trials, including information on the period of participation and his/her role (PI / Sub-Inv) in the clinical trial, the clinical trial code and phase, the therapeutic area along with the current status of the study (Completed, ongoing etc).
- the proof of ICH-GCP training of the involved person and the updates of knowledge (if applicable), including information on the time duration, the title/content, the organization provided the training, the type of training (F2F course, webtraining, etc), along with the certification date (if issued).

3.2.1.5 Italy

There are no specific requirements for the qualification of Italian investigators. Generally speaking, the principles reported in articles 45, 49 and 65 of the new European regulation have been already aplied in Italy.

There is no need to submit GCP training certificate to the CA and/or EC.

3.2.1.6 Poland

The investigator(s) in Poland shall fulfill the GCP-requirements (ICH-E6, revised 1996).

There is no need for a specific confirmation of GCP certificate from the investigators.

In Poland any clinical trials of a medicinal product must comply with the Good Clinical Practice (The Directive for Good Clinical Practice) and the Pharmaceutical Law (The Act of Pharmaceutical Law). A clinical investigator must have the medical license to practice and professional, research and clinical experience necessary to conduct a clinical trials. The investigator must be able to provide appropriate medical care for the participants. The primary investigator indicated by the sponsor is responsible for the performance of a clinical site (The Act on Pharmaceutical Law). The investigator's responsibilities are listed in the Directive for Good Clinical Practice. The investigator must be prepared for the clinical trial, including detailed knowledge of the medicinal product, the protocol, and other documents of a study. The clinical centre must have facilities necessary for a study. The clinical trial team must secure appropriate time for study procedures.

The Sponsor is responsible for selection of appropriate investigators competent in the care of patients in studied diseases. The sponsor is responsible for selecting appropriate monitoring team for a study. Monitoring of the trial must be according to GCP. The sponsor can delegate some or all of its responsibilities according to GCP to a CRO. This written agreement does not waive sponsor's responsibility for a study. The sponsor must organize the study according to the Pharmaceutical Law and Good Clinical Practice Act.

3.2.1.7 Portugal

The investigator(s) in Portugal have to fulfill the GCP-requirements (Chapter 4. GCP, ICH-E6, revised 1996) and the Portuguese Law nº 21/2014 from 16th of April (art. 2º-w); 10º and 12º) for Clinical Research.

In short:

- All investigators (Principal Investivators (PI) and Co-Investigators must have a degree in medicine and GCP certification (updated each 3 years).
- PI must have experience in conducting clinical trials, preferably in the therapeutic area of the trial under submission.
- The PI is the accountable person for conducting the trial, choose the co-investigators and clinical trial staff and may delegate task and responsabilities to them.

All the documents proving investigators' degree, CGP certification, experience and delegation of tasks must be delivered to the Ethical Commission (CEIC) when submitting the trial.

3.2.1.8 UK

The investigator(s) in United Kingdom have to fulfill requirements of GCP (Chapter 4.1 GCP, ICH-E6, revised 1996) and "The Medicines for Human Use (Clinical Trials) Regulations 2004".

3.2.2 Essential technical equipment at the trial sites and involvement of other facilities in the trial

Participating trial centres have to be specialized in the treatment of cardiac disease and fully equipped for cardiac MRI and diagnostic electrocardiography.

There will be two core labs responsible for analysing the imaging data relating to the primary endpoint:

- MRI core lab - performing the analyses regarding a change in left ventricular mass index
- Echocardiography core lab - performing data analyses assessing diastolic function (E/e')
- Spiroergometry - performing data analyses assessing the maximal exercise capacity

Additionally there will be a central lab, analysing the blood samples from the main study at UCL.

For randomisation and data capture each trial site needs internet access.

Participation in the scientific side projects requires equipment for arterial microtonometry (determination of endothelial function) and/or capture of FDG-PET-scans (abundance/activity of brown/beige fat).

3.3 Trial Sites and Number of Trial Subjects

There will be nine clinical trial sites in eight European countries participating in the BETA3_LVH trial.

It is anticipated that each trial centre will recruit about 30-40 patients, so that the overall recruitment goal of 296 patients (for details see chapter 9.4) can be reached.

3.4 Expected Duration of Trial

Each patient will be treated with placebo/mirabegron over a period of 12 months. Thus trial participation encompasses 52 weeks per patient. There will be no long-time follow-up.

The recruitment period will take 51 months for the recruitment of 296 patients (see chapter 9.4 for details on the sample size), thus the treatment period in the trial from first patient in to last patient out is expected to take 63 months. Trial recruitment will start after approval by the corresponding authorities in the different participating countries.

Trial start is defined as first patient in and the end of the trial is reached with the last visit of the last patient (LPLV).

3.5 Premature Termination of the Trial

The proceeding for the premature termination of the trial for a single patient is described in chapter 6.5

3.5.1 Termination of the Trial at a Single Site

The trial can be aborted at a single site if

- the protocol is not adhered to,
- the quality of data is deficient,
- there is inadequate recruitment.

The coordinating investigator in agreement with the steering committee decides whether or not to exclude the site, together with the sponsor and biometrician if appropriate.

Since BETA3_LVH is an European trial, the trial might be terminated in each involved country, if the responsible ethics committee and/or federal authority revokes its approval or terminates the trial in that country.

Investigators and sites no longer participating in the trial must inform the coordinating investigator immediately and, according to the grant agreement, should provide justification for the decision. Further treatment of patients still involved in the study is to be arranged together with the coordinating investigator.

3.5.2 Termination of the Whole Trial or of Individual Arms of the Trial

The trial can be terminated prematurely by the trial’s sponsor based on the recommendations of the DSMB (see chapter 13.5.1) in the event of

- evidence of study-related adverse effects (based on regular safety analyses)

- factors internal or external to the study that compromise the ethics of the study or impact patient safety (e.g. protocol violations, data unmasking, newly available scientific or therapeutic developments)
- insufficient overall compliance with the study protocol and the goals for recruitment and retention (including related gender issues)

The final decision regarding the premature termination of the trial will be made by the coordinating investigator/the sponsor or his/her authorized representative.

Since the trial is subject to Belgian drug law, the approval can be rescinded or the study can be terminated by the responsible federal authority (Federal Agency for Medicines and Health Products (FAMHP)) or the responsible ethics committee ("Comité d'Ethique Hospitalo-Facultaire" of Université Catholique de Louvain).

In addition, according to the Grant Agreement with the European Commission, the commission may terminate the trial, if the requirements of the grant agreement are not met by the beneficiaries.

4 TRIAL SUBJECTS

The target population are patients with structural heart disease at high risk for progressive hypertrophic cardiac remodelling leading to HFpEF, characterized by the following eligibility criteria.

4.1 Inclusion Criteria

- Age between 18 and 90 years
- Morphological signs of structural cardiac remodelling by echocardiography, i.e. increased LV mass index (≥95 g/m^2 or higher for female; ≥115 g/m^2 or higher for male subjects (Ponikowski et al. 2016)) or end-diastolic wall thickness ≥13 mm in at least one wall segment
- Written informed consent
  For subjects unable to read and/or write, oral informed consent observed by an independent witness is acceptable if the subject has fully understood oral information given by the Investigator. The witness should sign the consent form on behalf of the subject.

This patient population is known to be most likely to develop progressive hypertrophic cardiac remodelling and/or HFpEF (Komajda, Lam, Carolyn S P 2014).

4.2 Exclusion Criteria

- Unstable arterial hypertension with systolic BP≥160 mm Hg **and/or** diastolic BP≥100 mm Hg[1] (confirmed at three consecutive office measurements in sitting position); if so, the patient may be re-screened after optimization of anti-hypertensive treatment
- Hypertensive patients not under stable therapy according to current guideline algorithm (Mancia et al. 2013) (including stable medication for at least 4 weeks before inclusion)
- Documented ischemic cardiac disease:
  - current angina pectoris or

[1] In case of discrepancy between ambulatory and in office blood pressure, the office assessment prevails.

  - ischemia on stress test or
  - untreated coronary stenosis >50% or
  - history of acute myocardial infarction (AMI) or
  - coronary artery bypass graft (CABG, < than 3 months prior to screening) or
  - percutaneous transluminal coronary angioplasty (PTCA) less than 3 months prior to screening.
- Patients with uncontrolled recurrent persistent and permanent atrial fibrillation (AF) according to AHA/ACC/ESC guidelines (Dixon et al. 2005) (with a heart rate > 100/min, RACE II - (Groenveld et al. 2013, 2013)). If AF with HR>100/min, the patient may be rescreened after treatment for rate control.
- History of hospitalization for overt heart failure within last 12 months
- History of high degree impulse conduction blocks (> 2nd degree AV block type 2)
- Patients after heart transplantation
- Genetic hypertrophic or dilated cardiomyopathy
- Dysthyroidism[1]
- Severe valvulopathy (less than 1 cm^2 aortic valve area, mitral insufficiency of severe grade at Doppler echo)
- Congenital valvulopathies
- Patients with a known history of QT prolongation (QT>450ms)or patients with documented QT prolongation (QT>450 ms) while taking medicinal products known to prolong the QT interval.
- NYHA-class > II
- BMI > 40 kg/m^2
- EF < 50%, regardless of symptoms
- Known other cause (i.e. COPD) of respiratory dysfunction; patients under positive pressure (CPAP) treatment for sleep apnea syndrome may be included, provided they have been efficiently controlled by CPAP for at least one year before inclusion in the study
- Moderate renal impairment defined as eGFR < 30 ml/min[2]
- Abnormal liver function tests (AST or ALT >2 X upper normal limit or patients with known hepatic impairment defined as Child-Pugh class B or higher)
- Type I diabetes, complicated type II diabetes (i.e. with documented coronary macroangiopathy , cfr exclusion criterion 1 or documented other vascular complication) (National Diabetes Education Initiative - NDEI).
- Patients with anaemia (male: Hb <130 g/l, female: Hb <120 g/l)
- Patients with bladder outlet obstruction
- Patients using antimuscarinic cholinergic drugs for treatment of OAB
- Current use of digitalis, bupranolol, propranolol, nebivolol (known to interfere with β3AR signalling)

  Note: patients are allowed to take a β(1-2)-blocker, other than the drugs listed above (for explanation, see chapter 5.4.5).
- Patients continuously treated with Sildenafil or other PDE5 inhibitors.
- Current use of antifungal azole derivatives (fluconazole, itraconazole, miconazole, posaconazole, voriconazole) (known inhibitors of CYP3A4, the main metabolizer of mirabegron)

---

[1] there will be a clinical screening for signs and symptoms of dysthyroidism at inclusion and TSH will be measured at screening.

[2] GFR (mL/min/1.73 m^2) = 175 × $(S_{cr})^{-1.154}$ × $(Age)^{-0.203}$ × (0.742 if female) × (1.212 if African American), From http://nkdep.nih.gov/lab-evaluation/gfr/estimating.shtml#mdrd-study-equation

- Current treatment with mirabegron or indication for future treatment with mirabegron due to other indications
- Contraindication for MRI (e.g., defibrillator, ferromagnetic devices or severe claustrophobia, pacemaker – the latter only if MRI is contraindicated)
- Pregnant or nursing women
- Women of child bearing potential[1] without highly effective contraceptive measures (Clinical Trial Facilitation Group (CTFG) 9/15/2014):
    - combined (estrogen and progestogen containing) hormonal contraception associated with inhibition of ovulation (oral, intravaginal, transdermal)
    - progestogen-only hormonal contraception associated with inhibition of ovulation (oral, injectable, implantable)
    - intrauterine device (IUD)
    - intrauterine hormone-releasing system (IUS)
    - bilateral tubal occlusion
    - vasectomised partner
    - sexual abstinence (only if in agreement with the preferred and usual lifestyle of the subject)

  while participating in the trial. There are no known interactions of the trial medication and hormone-based methods of contraception. In particular, no clinically relevant interactions have been observed when mirabegron was co-administered with therapeutic doses of a combined oral contraceptive medicinal product containing ethinylestradiol and levonorgestrel.
- Participation in any other interventional trial
- Patients unable to give informed consent (people under legal guardianship)
- Patients placed in an institution by official or court order
- Contraindication to mirabegron (e.g. hypersensitivity) or any other components of the trial medication

Possible interaction with other medication will be discussed in chapter 5.4.5.

NOTE: In case of current treatment with one of the excluded drugs, patients can be rescreened after a wash out period of 3 half-lives.

4.3 Justification for the Inclusion of vulnerable Populations

This clinical trial will not include particularly vulnerable individuals (minors, pregnant and nursing women, persons unable to fully understand the scope and procedures of the clinical trial, patients unable to give informed consent). However, there will be elderly people included into the trial (>65 years of age), since data coming from this study should mirror normal clinical practice and the patient population usually suffering from structural heart disease at high risk for progressive hypertrophic cardiac remodeling at risk of developing HFpEF mostly belongs to this age group. These patients will only be included, if they are capable of giving informed consent and are not under legal guardianship.

[1] A woman is considered of childbearing potential (WOCBP), if she is fertile, i. e. following menarche and until becoming post-menopausal unless permanently sterile. Permanent sterilisation methods include hysterectomy, bilateral salpingectomy and bilateral oophorectomy. A postmenopausal state is defined as no menses for 12 months without an alternative medical cause. A high follicle stimulating hormone (FSH) level in the postmenopausal range may be used to confirm a post-menopausal state in women not using hormonal contraception or hormonal replacement therapy. However in the absence of 12 months of amenorrhea, a single FSH measurement is insufficient.

4.4 Participation in more than one Clinical Trial

During the verification of the inclusion and exclusion criteria the investigator/ his deputy or authorised medical staff of the study team checks if the patient is currently participating in any other interventional clinical trial. Should this be the case, the patient will not be included. Moreover, by signing the informed consent form, the patient confirms that he/she is not participating in any other interventional clinical trial simultaneously.

4.5 Statement on the Inclusion of Dependent Individuals

During the screening procedure, all patients will be interviewed concerning any potential relationship to the investigator/his deputy or medical staff of the study team, to the coordinating investigator or the sponsor. Should any relationship exist, the patient will not be included in the trial.

4.6 Rationale for Gender Distribution

Heart disease has been defined as a primarily male disease, and “evidence-based” clinical standards have been created based on male pathophysiology and outcomes. As a result, women are often mis- and under-diagnosed (Regitz-Zagrosek, Seeland 2011). Analysing sex and gender in heart disease has required formulating new research questions about disease definitions, symptoms, diagnosis, prevention strategies, and treatments. Once sex and gender were factored into the equation, knowledge about heart disease increased dramatically. As is often the case, including women subjects—of diverse social and ethnic backgrounds—in research has led to a better understanding of disease in both women and men. HFpEF, the disease under consideration here, is no exception (Lam et al. 2012), since it particularly affects aging women, but is not efficiently treated by evidence-based medications that are effective in HFrEF, where mostly male subjects have been enrolled. This points to specific pathophysiologic factors that may more prominently affect women and that, if unveiled, would help to better understand and treat men, too. As such, our trial on patients (mostly women) at risk of developing/worsening HFpEF fulfils the sex and gender-specific consideration inherent to this topic.

Accordingly, although we do not intend to stratify according to sex in the randomisation protocol, at the completion of the trial we will operate an analysis of all data to explore whether sex affects the outcomes and/or treatment effects; if such sex factor appears, we will explore whether this is associated with gender-specific additional variables, such as smoking habits (which may differ between genders), or differences in background treatment.

Effects like these were seen in the I-PRESERVE trial, with prominent sex differences in baseline characteristics, e.g. women were older and more likely to be obese and have hypertension, whereas men were more likely to be affected by ischemic heart disease or atrial fibrillation. Sex also affected the outcomes, since women had a better overall prognosis. The presence of comorbidity, however, seemed to attenuate the difference (Lam et al. 2012).

5 INVESTIGATIONAL PRODUCT

5.1 Trial Drugs

Verum

Generic Name:	Betmiga®
Adress Manufacturer:	Universitätsklinikum Leipzig - AöR<br>Apotheke<br>Liebigstr. 20<br>D-04103 Leipzig
Drug allocation:	Pharmacy of the University Hospital Leipzig<br>Liebigstr. 20<br>D-04103 Leipzig
Batch number:	not yet available
Formulation used:	50 mg tablets in capsules
Packaging:	7 capsules/blister, 4 blisters/box<br>= 28 capsules
Storage conditions:	room temperature
Stability:	1.5 years
Preventive measures/Incompatibility:	none known

Placebo

Name:	P-Tabletten™ weiß 7 mm Lichtenstein
Adress Manufacturer:	Universitätsklinikum Leipzig - AöR<br>Apotheke<br>Liebigstr. 20<br>D-04103 Leipzig
Drug allocation:	Pharmacy of the University Hospital Leipzig<br>Liebigstr. 20<br>D-04103 Leipzig
Batch number:	not yet available
Formulation used:	tablets in capsules
Packaging:	7 capsules/blister, 4 blisters/box<br>= 28 capsules
Storage conditions:	room temperature (15- 25°C)
Stability:	1.5 years

5.2 Packaging and Labelling of the Trial Drug

Betmiga® or placebo tablets will be packed in capsules and then blistered. Packing will be in monthly boxes (four blisters with 7 capsules each).

Each blister will be labelled with:

- Protocol-name
- EudraCT-number
- Name of trial product
- Med-code
- Batch-number
- Expiry date
- Sponsor
- CRO

Protocol No: Beta3_LVH

EudraCT No: 2015-003146-75

Mirabegron 50 mg / Placebo

Contains 7 Capsules for oral use

Med Code: ____ **Pat-ID: __|__|__|- __|**

Batch No.: *xxxxxxxx* Expiry Date: *mm/jjjj*

Sponsor: Université catholique de Louvain, Place de l'Université 1, BE-1348 Louvain la Neuve,

CRO: University of Leipzig, Clinical trial center Leipzig KKS, Haertelstr. 16-18, D-04107 Leipzig

Figure 2: Sample of blister-label

Each box will be labelled with (European Commission 2/3/2010):

- "For Clinical Trial Use only!"
- Protocol name
- EudraCT-number
- Name of trial product
- Med-code
- Batch-number
- Expiry date
- "Keep out of sight and reach of children!"
- Dose
- Storage condition
- Sponsor
- CRO

For Clinical Trial use only!	
Protocol Beta3_LVH	
EudraCT No: 2015-003146-75	
**Mirabegron 50 mg / Placebo**	
28 Capsules for oral use	
**Med Code:____**	**Pat-ID: __ __ __ - __**
**Batch No** *xxxxxxxx* Expiry Date: *mm/jjjj*	
Keep out of the sight and reach of children!	
Dose according to the study protocol!	
Storage at room temperature (15-25°C)	
**Sponsor:** Université catholique de Louvain, Place de l'Université 1, BE-1348 Louvain la Neuve	
**Tel.:** *+32-2-7645260*	
**CRO:** University of Leipzig, Clinical trial center Leipzig KKS, Haertelstr. 16-18, D-04107 Leipzig	
**Tel.:** *+49 (0)341 9716250*	

Figure 3: Sample of box-label

The traded commodity of the investigational product (Betmiga®) will be received from Astellas Pharma BeLux and manufactured, packaged and labelled by the pharmacy of the University Hospital Leipzig, Liebigstr. 20, D-04103 Leipzig according to the standards of Good Manufacturing Practice (GMP).

The traded commodity of the placebo (P-Tabletten™) will be received from Winthrop Arzneimittel GmBH (65927 Frankfurt am Main, Germany) and manufactured, packaged and labelled by the pharmacy of the University Hospital Leipzig, Liebigstr. 20, D-04103 Leipzig according to the standards of Good Manufacturing Practice (GMP).

The study drug will be labelled as required by the ICH-GCP Guideline E6 (European Commission 2/3/2010). The labelling will be in the official language(s) of the countries in which the trial takes place.

The master label is part of the documents that will be submitted to the responsible federal authority.

5.3 Drug Accountability

Blinded study drugs (Betmiga®, placebo) will be provided to the trial sites by the Pharmacy of the University Hospital Leipzig, Germany.

Each study site will receive enough trial medication to treat 10 patients at the beginning of the study. Depending on the number of patients being included in the study, each trial site will receive additional trial product after a written request to the CTC Leipzig, which will inform the pharmacy on the need of medication at the site.

Expended blisters will be replaced.

The investigator will confirm receipt of the study drug in writing and will use the study drug only within the framework of this clinical study and in accordance with this study protocol. He/she will keep a record of the study drug dispensed to and – if applicable - returned from each patient.

Receipt, distribution, return and potentially destruction of the study drug must be properly documented on the forms provided by the sponsor, giving the following information: study protocol number, sender, receiver, date, mode of transport, quantity, batch number, expiration date and retest date, if applicable.

The sponsor or its designee will monitor the drug accountability records at regular points during the study and will perform a drug reconciliation at the end of study.

5.4 Administration of the Study Drug

5.4.1 Procedures

Handling guidelines for the application of the trial drug/placebo:

- patients will receive one capsule daily (50 mg Betmiga®/placebo) in the morning
- treatment commences over one year resulting in a final dose of 18,25 g mirabegron per patient and year

5.4.2 Compliance

At each visit, the patients receive the amount of the trial medication required until the next appointment (including a reserve). The investigator examines the record at each visit and takes back the empty packages or unused medication. The monitor appraises the Drug Accountability and thus compliance of the patients as part of the on-site checks.

5.4.3 Dealing with Side-effects

5.4.3.1 Summary of the safety profile

The safety of Betmiga was evaluated in 8433 patients with OAB, of which 5648 received at least one dose of mirabegron in the phase 2/3 clinical program, and 622 patients received Betmiga for at least 1 year (365 days). In the three 12-week phase 3 double blind, placebo controlled studies, 88% of the patients completed treatment with Betmiga, and 4% of the patients discontinued due to adverse events. Most adverse reactions were mild to moderate in severity.

The most common adverse reactions reported for patients treated with Betmiga 50 mg during the three 12-week phase 3 double blind, placebo controlled studies are tachycardia and urinary tract infections. The frequency of tachycardia was 1.2% in patients receiving Betmiga 50 mg. Tachycardia led to discontinuation in 0.1% patients receiving Betmiga 50 mg. The frequency of urinary tract infections was 2.9% in patients receiving Betmiga 50 mg. Urinary tract infections led to discontinuation in none of the patients receiving Betmiga 50 mg. Serious adverse reactions included atrial fibrillation (0.2%).

Adverse reactions observed during the 1-year (long term) active controlled (muscarinic antagonist) study were similar in type and severity to those observed in the three 12-week phase 3 double blind, placebo-controlled studies.

The placebo tablet contains Lactose and Cellulose (like the verum) and thus might cause intestinal problems. However, due to the low dose administered, these effects are expected to occur rarely.

5.4.3.2 Tabulated list of adverse reactions (mirabegron)

The table below reflects the adverse reactions observed with mirabegron in the three 12-week phase 3 double blind, placebo controlled studies.

The frequency of adverse reactions is defined as follows: very common (≥1/10); common (≥1/100 to <1/10); uncommon (≥1/1,000 to <1/100); rare (≥1/10,000 to <1/1,000); very rare (<1/10,000). Within each frequency grouping, adverse reactions are presented in order of decreasing seriousness.

**MedDRA System organ class**	**Common**	**Uncommon**	**Rare**	**Very rare**	**Not known (cannot be estimated from the available data)**
Infections and infestations	Urinary tract infection	Vaginal infection<br>Cystitis			
Psychiatric disorders					Insomnia*<br>Confusional state *
Nervous system disorders	Headache*<br>Dizziness*				
Eye disorders			Eyelid oedema		
Cardiac disorders	Tachycardia	Palpitation<br>Atrial fibrillation			
Vascular disorders				Hypertensive crisis*	
Gastrointestinal disorders	Nausea*<br>Constipation*<br>Diarrhoea*	Dyspepsia<br>Gastritis	Lip oedema		
Skin and subcutaneous tissue disorders		Urticaria<br>Rash<br>Rash macular<br>Rash papular<br>Pruritus	Leukocytoclastic vasculitis<br>Purpura<br>Angioedema*		
Musculoskeletal and connective tissue disorders		Joint swelling			
Renal and urinary disorders			Urinary retention*		
Reproductive system and breast disorders		Vulvovaginal pruritus			
Investigations		Blood pressure increased<br>GGT increased<br>AST increased<br>ALT increased			

*observed during post-marketing experience

5.4.3.3 Guidelines in case of elevated BP under study drug

Case 1: HBP (SBP>180 and/or DBP>110) under study drug:

- STOP study drug
- adjust background therapy according to standard guidelines
- see patient within next 2 weeks
- if BP stabilized: RE-INTRODUCE study drug (same label)
- see patient after 2 weeks: if BP stable: PROCEED with trial therapy
- If SBP>180 and/or DBP >110: STOP study drug PERMANENTLY; continue patient follow-up
- Document SAE

Case 2: HBP (SBP>160 and/or DBP>100) under study drug

- keep study drug
- adjust background therapy according to standard guidelines
- see patient after 2 weeks: if BP stabilized: PROCEED with trial therapy
- If HBP >180 and/or DBP >110 mmHg: see case 1
- If HBP>160 and/or DBP >100 mmHg: see case 2: allow 1 more adjustment of therapy
- see patient within next 2 weeks: if BP stabilized: PROCEED with trial therapy
- If HBP>160 and/or DBP>100 mmHg: STOP study drug PERMANENTLY; continue patient follow-up
- Document AE

5.4.3.4 Guidelines in case of abnormal laboratory values (renal and/or hepatic impairment, anemia)

<u>Renal impairment:</u>	eGFR < 30 ml/min
<u>Hepatic impairment:</u>	AST or ALT > 2x the upper normal limit
<u>Anemia:</u>	Hb< 130 g/L in males, Hb < 120 g/L in females

- If a patient at least meets one of these 3 limits, the IMP will be stopped, the cause of anomaly will be actively sought and the patient treated accordingly if applicable/possible.
- Once anomalies are corrected the IMP will be RE-INTRODUCED.
- see patient within next 2 weeks: if laboratory values are stable: PROCEED with trial therapy

- If laboratory values worsen again: STOP study drug PERMANENTLY; continue patient follow-up
- Document SAE

5.4.4 Alternative Medication

Substitution of the trial product by other medication is not permitted during trial participation of a patient.

5.4.5 Allowed concomitant medication

In case of necessary treatment of included patients, the current guidelines for treatment will be followed. In the trial target population this will be mostly the treatment of arterial hypertension (Mancia et al. 2013), diabetes (National Diabetes Education Initiative - NDEI) and/or atrial fibrillation (Camm et al. 2010).

To be able to follow the treatment guidelines, among the categories of beta-blockers or anti-arrhythmic drugs, **the following products are allowed for treatment during trial participation**:

- Beta-blocker: Acebutolol, Atenolol, Betaxolol, Bisoprolol, Carvedilol, Celiprolol, Esmolol, Labetolol, Metoprolol, Pindolol.
- Treatment of AF or other arrhythmias: Amiodarone, Cibenzoline, Disopyramide, Flecaïnide, Propafenon, Sotalol.
- Calcium-antagonist:Diltiazem
- I_f current inhibitor: Ivabradin

5.4.6 Counterindicated/Forbidden Concomitant Medication

There are no counterindicated medications listed in the SmPC of Betmiga®.

Medication not allowed during trial participation due to potential interactions and/or weakening of the expected effect is listed in the exclusion criteria (chapter 4.2).

Due to potential interactions with the IMP, **the following medicinal products are forbidden during trial participation**:

- Digitalis is prohibited because it inhibits Na-K ATPase which is a target for the beneficial effect of mirabegron (Galougahi et al. 2012).
- Bupranolol (Dessy et al. 2004), propranolol (Gauthier et al. 2011), nebivolol (Dessy et al. 2005) are prohibited Beta-blockers since they are known to interfere with β3AR signalling.
- Sildenafil or other PDE5 inhibitors taken continuously for treatment of pulmonary hypertension
- Current use of antifungal azole derivatives (e. g.: fluconazole, itraconazole, miconazole, posaconazole, voriconazole) since they are known inhibitors of CYP3A4, the main metabolizer of mirabegron (Center for Drug Evaluation and Research; Repertoire Commente des Medicaments) and ketoconazole, ritonavir or clarithromycin (listed in the SmPC of Betmiga®).
- Antimuscarinic cholinergic drugs for treatment of OAB (due to contraindications of mirabegron and risk of inducing urinary retention)

5.4.7 Overdose and Abuse

Mirabegron has been administered to healthy volunteers at single doses up to 400 mg. At this dose, adverse events reported included palpitations (1 of 6 subjects) and increased pulse rate exceeding 100 beats per minute (bpm) (3 of 6 subjects). Multiple doses of mirabegron up to 300 mg daily for 10 days showed increases in pulse rate and systolic blood pressure when administered to healthy volunteers.

Treatment for overdose should be symptomatic and supportive. In the event of a known or presumed overdose

- the IMP will be withdrawn for 5 days ($t_{1/2}$= 50 h),
- pulse rate, blood pressure, and ECG monitoring will be performed
- Once anomalies are corrected the IMP will be RE-INTRODUCED.

5.5 Blinding and Unblinding

The trial is a double-blind study. The trial medication and placebo will be manufactured by the Pharmacy of the University Hospital Leipzig (see chapter 5.1). Blinding will also be conducted by the Pharmacy of the University Hospital Leipzig following a randomisation list provided by Clinical Trial Centre Leipzig.

In order to preserve the blinding of the study, a minimum number of personnel will see the randomisation table and treatment assignments before the study is complete.

The information regarding the treatment arm of a patient will be kept in a sealed and light-proof envelope (emergency envelope). The envelopes will be kept at the pharmacovigilance department of the Clinical Trial Centre Leipzig, and each trial centre will receive the emergency envelopes for the patients treated at the site. At the trial site, the envelopes have to be kept in the investigator site file (ISF). Another set of envelopes will be sent to the head of the DSMB.

Unblinding for adverse events may be performed ONLY if an inevitable, intermediate medical intervention due to safety reasons requires knowledge of the patient’s treatment assignment. The investigator should make every effort to contact the coordinating investigator prior to unblinding a patient’s treatment assignment. If an investigator, site personnel performing assessments, or patient is unblinded, the patient must be discontinued from the study only if deemed necessary for safety reasons by the coordinating investigator.

If an investigator opens an emergency envelope:

- he has to document date and time of unblinding on the emergency envelope and sign it
- the opened envelope has to be kept in the ISF
- the unblinding has to be documented in the patient’s file as well as on the CRF

Unblinding in case of suspected unexpected serious adverse reactions (SUSARs) will follow the SOPs of the Clinical Trial Centre Leipzig and will be described in separate working instructions of the pharmacovigilance department. In case of unblinding by the pharmacovigilance department of the Clinical Trial Centre Leipzig, this will be documented in the pharmacovigilance database.

The hand-out of emergency envelopes as well as the return of unopened envelopes will be documented on special form sheets.

6 INDIVIDUAL TRIAL PROCEDURES

6.1 Patient Information and Informed Consent

The patient’s consent will be obtained by an approved trial physician before any trial specific medical procedures are performed with the patient. Patients who are considered not

competent to consent to participate, e.g. due to an insufficient level of understanding, cannot participate in the study.

In accordance with international guidelines, the informed consent of trial participants will be in writing (written, dated and signed by the person performing the interview referred to below, and by the subject).

The patient’s consent must refer explicitly to the collection and processing of health-related data. Therefore, the patient will be informed explicitly about the purpose of collecting the data and scope of what is to be collected and that personal data, including health related data, will be stored and used for analyses in a pseudonymized form.

Before obtaining informed consent, the potential trial participant will receive information regarding the clinical trial in an interview. This has to cover the following items:

- the nature, objectives, benefits, implications, risks and potential inconveniences of the clinical trial
- the expected duration of the subject's participation in the clinical trial
- the information that the patient may withdraw his/her consent to participate at any time without giving reasons. The patient is to be informed that in case of revocation of his/her consent, the stored data may be used further, as may be necessary to
    - assess effects of the medicinal product being tested,
    - guarantee that the patient’s personal interests are not adversely affected,
    - comply with the requirement to provide complete authorisation documentation.
- possible treatment alternatives
- follow-up measures in case of early termination of the trial for the patient or overall
- the applicable damage compensation system in case of damage to a patient
- the right on data access, rectification and withdrawal of personal data

The subject will have the opportunity to ask questions at any moment.

Adequate time will be provided for the subject to consider his or her decision.

The interview with a potential trial participant has to be performed by a member of the investigating team qualified for this task under the national law of the Member State where the recruitment takes place.

6.1.1 Withdrawal of Informed Consent

Patients may withdraw their consent to participate at any time without giving reasons. Nevertheless, the patient should be asked for the reason of the premature termination after being informed that he/she does not need to do so. Information as to when and why a patient was registered/ randomized and when he/she withdrew consent must be retained in the documentation.

The patient is to be informed that in case of revocation of his/her consent, the stored data may be used further, as may be necessary to

- assess effects of the drug being tested,
- guarantee that the patient’s personal interests are not adversely affected,
- comply with the requirement to provide complete authorisation documentation.

6.2 Enrolment in the Trial

Patients will be recruited at the participating trial sites, which are all specialized in cardiology. Once potential patients are identified by trial physicians, they will be asked for trial participation and informed consent. If the patients are eligible for the trial according to the eligibility criteria, a web based randomisation will be performed and the patient assigned to a treatment arm.

The assessments and procedures essential for trial inclusion are depicted in table 1: Schedule of Assessments and Procedures. After successful randomisation the patient will receive the trial product including the information on the correct use.

6.2.1 Discovery of a Violation of the Eligibility Criteria after the Fact

In general, the subsequent detection of the violation of eligibility criteria is not a reason for premature withdrawal of the patient from the trial therapy or from the whole trial.

If after randomisation it is discovered that the patient was not eligible at the time of randomisation, this has to be reported to the Clinical Trial Centre Leipzig-Data Management and biometrics as soon as possible. The Clinical Trial Centre Leipzig informs the coordinating investigator immediately as to what is to be done with the patient. Only in case of major violation *will the coordinating investigator report this to the ethic committee. The patient’s data will continue to be recorded unless the patient revokes his/her informed consent. For procedures after premature trial termination of a single patient see chapter 6.4.

6.3 Description of the Treatment Procedures

There will be six visits within the trial, at baseline, month one, three, six, nine and twelve months after randomisation. For a description of the treatment procedures see below

<u>Screening (max. one month before baseline):</u>

- determination of eligibility criteria
- informed consent
- registration
- medical history
- documentation of concomitant diseases and medication
- clinical assessment (including BP measurement, determination of NYHA functional class and pot. symptoms, BMI, heart rate)
- ECG
- standard echocardiography
- local laboratory testing (hemogram, liver function, renal function)
- pregnancy test - if applicable
- measurement of thyroid stimulating hormone

<u>Baseline:</u>

- confirmation of eligibility criteria
- documentation of concomitant diseases
- randomisation
- clinical assessment (including BP measurement, 24 h ABPM, determination of NYHA functional class and pot. symptoms, BMI, heart rate, check of current concomitant

medication)

- extended echocardiography (see extended protocol)
- cardiac MRI (see extended protocol)
- maximal exercise capacity (by bicycle ergometry (see extended protocol))
- central laboratory testing (fasting glucose, HOMA-test, HbA1c, serum lipids for metabolism, Galectin3, GDF15, NT-proBNP, hsTnT as specific biomarkers)
- local laboratory testing (hemogram)
- handing out of trial medication and documentation thereof (drug accountability)
- capturing and documentation of adverse events – if applicable
- blood and urine-sampling for later scientific side projects

if applicable and patient takes part:

- sub-study: endothelial function (EndoPAT, fingertip Peripheral Arterial Tonometry, Hb-NO)
- sub-study: [^{18}F]-FDG-PET for brown adipose tissue visualization
- pregnancy test - if applicable

<u>Visit 1 (month 1 ±3 days):</u>

- clinical assessment (including BP measurement, determination of NYHA functional class and pot. symptoms, BMI, heart rate, check of current concomitant medication)
- local laboratory testing (liver function, renal function)
- capturing and documentation of adverse events – if applicable
- drug accountability (counting of used/returned medication, hand-out of new medication)
- blood and urine-sampling for later scientific side projects
- pregnancy test - if applicable

<u>Visit 2 (month 3 ±14 days):</u>

- clinical assessment (including BP measurement, determination of NYHA functional class and pot. symptoms, BMI, heart rate, check of current concomitant medication)
- local laboratory testing (liver function, renal function)
- central laboratory testing (fasting glucose, HOMA-test, HbA1c, serum lipids for metabolism, Galectin3, GDF15, NT-proBNP, hsTnT as specific biomarkers)
- drug accountability (counting of used/returned medication, hand-out of new medication)
- capturing and documentation of adverse events – if applicable
- pregnancy test - if applicable

<u>Visit 3 (month 6 ±14 days):</u>

- clinical assessment (including BP measurement, determination of NYHA functional class and pot. symptoms, BMI, heart rate, check of current concomitant medication)

- extended echocardiography (see extended protocol)
- cardiac MRI (see extended protocol)
- central laboratory testing (fasting glucose, HOMA-test, HbA1c, serum lipids for metabolism, Galectin3, GDF15, NT-proBNP, hsTnT as specific biomarkers)
- local laboratory testing (hemogram, liver function, renal function)
- drug accountability (counting of used/returned medication, hand-out of new medication)
- capturing and documentation of adverse events – if applicable
- blood and urine-sampling for later scientific side projects

if applicable and patient takes part:

- sub-study: endothelial function (EndoPAT, fingertip Peripheral Arterial Tonometry and Hb-NO)
- pregnancy test - if applicable

Visit 4 (month 9 ±14 days):

- clinical assessment (including BP measurement, determination of NYHA functional class and pot. symptoms, BMI, heart rate, check of current concomitant medication)
- capturing and documentation of adverse events – if applicable
- drug accountability (counting of used/returned medication, hand-out of new medication)
- blood and urine-sampling for later scientific side projects
- pregnancy test - if applicable

Visit 5 (month 12 ±14 days):

- clinical assessment (including BP measurement, 24 h ABPM, determination of NYHA functional class and pot. symptoms, BMI, heart rate, check of current concomitant medication)
- extended echocardiography (see extended protocol)
- cardiac MRI (see extended protocol)
- maximal exercise capacity (by bicycle ergometer see extended protocol)
- central laboratory testing (fasting glucose, HOMA-test, HbA1c, serum lipids for metabolism, Galectin3, GDF15, NT-proBNP, hsTnT as specific biomarkers)
- local laboratory testing (hemogram, liver function, renal function)
- drug accountability (counting of used/returned medication)
- capturing and documentation of adverse events – if applicable
- blood and urine-sampling for later scientific side projects

if applicable and patient takes part:

- sub-study: endothelial function (EndoPAT, fingertip Peripharel Arterial Tonometry and Hb-NO)
- sub-study: [^{18}F]-FDG-PET for brown adipose tissue visualization

- pregnancy test - if applicable

Visit 6 (month 13 ± 2 days), Telephone-Follow-Up:

- medical history/current medication
- capturing and documentation of adverse events – if applicable

6.4 Premature Termination of the Therapy for Individual Patients

Trial therapy (i.e. treatment with mirabegron resp. placebo) must be terminated prematurely in case

- a patient has an adverse event that would, in the investigator's judgment, make continued treatment with trial therapy an unacceptable risk
- a patient becomes pregnant
- a patient is worsening (and not responding to treatment) that contraindicates transiently or permanently the treatment with trial therapy (see stopping rules described in sections 5.4.3.3 or 5.4.3.4)..
- in the opinion of the investigator, a patient is judged to be significantly non-compliant with the requirements of the protocol, thereby endangering patient safety.
- the treatment blind is broken for a patient.
- at the judgment of the investigator for any other reason of medical prudence
- on request of the patient.

With exception of the rules described above, premature termination of therapy should be avoided. In case the patient misses the scheduled visits, the investigator may contact the patient directly, in order to motivate him/her for further continuation.

In case of premature termination of therapy it is necessary to document the reason of termination and the current condition of the patient.

All further study visits until month twelve will take place as planned and described above. **Termination of trial therapy does not mean that the patient is off-study.**

Our primary statistical analysis follows the intention to treat principle as close as possible. For a valid analysis it is of major importance to minimise the rate of drop-outs. Therefore, in patients which do not withdraw their consent, all study visits shall be performed as scheduled. In order to account for possible risks due to trial assessments, section 6.5.

6.5 Premature study termination for individual patients

All randomised patients will be followed up until month twelve (resp. thirteen). Premature termination of trial therapy does not lead to individual study termination (see chapter 6.4 for explanation).

The only circumstances in which a premature study termination (i.e. no further study visits) in a randomised patient is unavoidable are:

- a patient is worsening (and not responding to treatment) that contraindicates permanently participation in any trial visits
- withdrawal of informed consent,
- complete loss of contact to the patient or
- death of the patient.

Each premature termination of the trial has to be documented by the responsible investigator. If possible date, circumstances of, reason for the termination, and - if applicable - the final status of patient should be documented in detail and communicated to the ZKS Leipzig - KKS Data Management.

6.6 Plan for Further Treatment

Since mirabegron has no legal indication for cardiac disease so far; patients will be treated as usual after the individual's trial completion.

7 METHODS OF DIAGNOSTICS AND DATA SAMPLING

Detailed descriptions of shipping and storing of biological samples as well as transferring imaging-data to the core labs will be laid down in individual handling guidelines contained in the investigator site files.

8 ADVERSE EVENTS (AE/SAE)

8.1 Adverse Events (AE)

8.1.1 Definition

An Adverse Event (AE) is any untoward medical occurrence in a patient or clinical investigation subject administered a pharmaceutical product and which does not necessarily have to have a causal relationship with this treatment (ICH Expert Working Group 10/27/1994).

Adverse Events encompass any unfavourable and unintended sign (including an abnormal laboratory finding, for example), symptom, or disease that arose newly or worsened after the inclusion of the patient into the trial.

An adverse drug reaction of a medicinal product carrying a marketing authorization is defined in Article 2(n) of Directive 2001/20/EC as all "untoward and unintended responses to an investigational medicinal product which occurs at doses normally used".

8.1.2 Documentation and Reporting

Adverse events will be documented on special AE-forms from baseline until the last trial visit.

At each visit, the patient will be asked a non-leading question such as "Have you had any health problems since the last visit". All AEs reported in response to questioning, as well as AEs reported spontaneously will be recorded on the 'adverse event' page(s) in the CRF, regardless of causality. In addition, the patient will be trained to inform the clinical trial site of any health problems arising between visits by phone or personal visit.

If an AE fulfils any of the criteria for a SAE (see chapter 8.4.1 for SAE-definition), both the AE pages of the CRF and the SAE form must be completed. This applies to all SAEs, **whether or not they are considered to be related to study treatment.**

For both serious and non-serious AEs, documentation should be supported by an entry in the patient's heath record. Required information includes: the type of AE, seriousness of the event, an estimate of its severity, start date, date of resolution, actions required, outcome and an assessment of its relationship to study drug.

All abnormal physical and/or laboratory results which are considered to be clinically relevant by the investigator should be recorded as AEs. If an abnormal laboratory result meets any of the criteria for a SAE, this must also be reported on the SAE Form.

Adverse Events are classified by their severity, intensity and relationship to the IMP (see also chapter 19.1)

8.2 Safety Analysis

During the course of the trial, every patient will be monitored closely. On every visit this encompasses documenting Adverse Events as well as the following parameters:

- elevated blood pressure under treatment (see chapter 5.4.3.3; if SBP>180 mm Hg AND/OR DBP >110 mm Hg, the AE becomes an SAE)
- heart rate > 110 bpm with or without palpitations
- symptoms related to heart insufficiency (NYHA class, edema …)
- liver function on every visit by local lab

8.3 Concomitant Diseases

Concomitant diseases will be recorded at baseline and confirmed at the first regular trial visit at month 1. Any worsening of concomitant diseases will be regarded as adverse event.

Of particular importance in the BETA3_LVH-trial is high blood pressure, which has to be observed closely, and treated according to guidelines at inclusion, with as stable treatment as possible during trial.

8.4 Serious Adverse Events (SAE)

8.4.1 Definition

An Adverse Event is defined to be serious according to ICH-Guideline E2A, paragraph IIB (ICH Expert Working Group 10/27/1994), if it

- **results in death**,
- **is life-threatening**,

  Note: The term “life-threatening” in the definition of “serious” refers to an event in which the patient was at risk of death at the time of the event; it does not refer to an event which hypothetically might have caused death had it been more severe.

- **requires in-patient hospitalization or prolongation of existing hospitalization**,
- **results in persistent or significant disability/incapacity or**
- **is a congenital abnormal/birth defect**

including events which would have led to one of the above if left untreated.

Medical and scientific judgement should be exercised in deciding whether expedited reporting is appropriate in other situations, such as important medical events that may not be immediately life-threatening or result in death or hospitalisation but may jeopardise the patient

or may require intervention to prevent one of the other outcomes listed in the definition above. These should also usually be considered serious.

In the present trial, a rise of blood pressure above 180 mm Hg systolic and 110 mm Hg diastolic despite stable background anti-hypertensive treatment will also be considered as serious adverse event.

In addition, a pathological laboratory finding for liver and/or kidney function leading to a stop of trial therapy according to the criteria listed under chapter 5.4.3.4 has to be documented as SAE.

8.4.2 Documentation and Reporting Obligations: INVESTIGATOR

Serious Adverse Events will be documented from baseline until the last trial visit.

Serious Adverse Events have to be documented on the SAE-forms and the investigator must report them to the pharmacovigilance department of the Clinical Trial Centre Leipzig immediately. If more information about the SAE becomes available later, it must also be reported to the pharmacovigilance department of the Clinical Trial Centre Leipzig immediately.

In the event of a patient's death, the investigator/ the deputy or the authorised medical staff will provide the leading ethics committee(s), all involved ethics committees in multi-centred trials, the responsible federal authorities and the sponsor with all further information needed to fulfil their tasks **upon request**.

In all reports, personal data are to be pseudonymized by using the patient's identification code. It must be possible to relate the initial and all follow-up reports to each other by means of the patient identification number, name and address or the like.

The investigator, the deputy or the authorised medical staff must report every Serious Adverse Event (incl. death not causally related to using the trial drug) as soon as it is known to the following address:

Clinical Trial Centre Leipzig/ Arzneimittelsicherheit
Universität Leipzig
Zentrum für Klinische Studien Leipzig – KKS
Haertelstr. 16-18, 04107 Leipzig
Phone: +49/341/97-16129
E-mail: pharmacovigilance@zks.uni-leipzig.de

**Fax: +49/341/97-16278**

8.4.3 Documentation and Reporting Obligations: SPONSOR

After the Clinical Trial Centre Leipzig receives the SAE, it is immediately passed on to the coordinating investigator for the medical assessment.

The coordinating investigator forms a second medical opinion of the SAE with respect to causal relationships and the decision as to whether or not it was expected, as described in Chapter 19.1.3 and 19.1.4 and forwards the second opinion, within two days of its arrival to the Clinical Trial Centre Leipzig.

At the Clinical Trial Centre Leipzig the SAE data are entered into the SAE database immediately and the MedDRA coding takes place simultaneously.

Then forwarding as per law and as described in Chapter 8.5 only for Suspected Unexpected Serious Adverse Drug Reactions (SUSARs) takes place.

Details of the sponsor's documentation and reporting obligations will be specified in a special, trial-specific pharmacovigilance plan, which will be written and finalised alongside with this protocol, if possible.

8.5 Periodic Reports

8.5.1 Annual Safety Report

The sponsor writes a safety report annually or upon request (Annual Safety Report, ASR[1]). The sponsor sends the report about the safety of the trial medication to the leading ethics committee and the federal authorities.

The key date is the date of the first authorization of the clinical trial by any of the federal authorities it was applied for. All data obtained up to this date (each year) will be included in the ASR. Beginning with the key date, there is a time-limit of 60 days for the preparation and submission of the ASR.

The ASR will be prepared by the Clinical Trial Centre Leipzig (project manager, responsible biometrician and PV data manager). The final report will be released in co-operation with the principal investigator/sponsor representative.

8.5.2 Periodic SUSAR Reports for Ethic Committees

Because of the procedures concerning SUSAR-reporting (see 8.6.2) it may be necessary to report all SUSARs from other Member States periodically (at least every 6 months) as a line listing accompanied by a brief report by the sponsor highlighting the main points of concern. These periodic reports should only include SUSARs reported within the period covered by the report.

If this is required by any of the involved member states, the SUSAR-reports will be prepared by the Clinical Trial Centre Leipzig (project manager, responsible biometrician and PV data manager). The final report will be released in co-operation with the principal investigator/ sponsor representative.

8.6 Suspected Unexpected Serious Adverse Reactions (SUSAR)

8.6.1 Definition

Suspected Unexpected Serious Adverse Drug Reactions (SUSARs) are side-effects (probably or definitely connected with the administration of the investigational product), the nature or severity of which are inconsistent with the information available about the product. Information about the trial product is contained in the Investigator's Brochure or the SmPC (Summary of medicinal Product Characteristics).

8.6.2 Documentation und Reporting Obligations

Information *for SPONSOR*

The sponsor submits all information available about a SUSAR, including the treatment arm of the patient, immediately to the leading ethics committee, the responsible federal authorities, and to all participating primary investigators, at the latest within 15 calendar days after the event becomes known.

For every SUSAR that results in death or a life-threatening condition, the leading ethics

[1] See "Detailed guidance on the collection, verification and presentation of adverse reaction reports arising from clinical trials on medicinal products for human use").

committee, the federal authority, and all participating investigators must be informed by the sponsor within 7 calendar days after the event becomes known. Additional information has to be given within 8 further calendar days

Details of the sponsor's documentation and reporting obligations will be specified in a special, trial-specific pharmacovigilance plan which will be written and finalised alongside with this protocol, if possible.

Information for INVESTIGATOR

The investigator passes down all relevant information concerning the SUSAR to all participating trial investigators at his/her trial centre. This has to be confirmed by the investigator by signing an acknowledgement document.

To ensure that the investigators and all other trial personnel remain "blind" in the case of a SUSAR, the investigating centres will be sent all potential SUSARs without information concerning the administered drug (regardless of whether it is verum or placebo).

8.7 Other Safety Relevant Issues

Other safety issues also qualify for expedited reporting where they might materially alter the current benefit-risk assessment of an investigational medicinal product or would be sufficient to consider changes in the investigational medicinal products administration or in the overall conduct of the trial, for instance:

New events related to the conduct of a trial or the development of an IMP likely to affect the safety of subjects, such as:

- a serious adverse event which could be associated with the trial procedures and which could modify the conduct of the trial,
- a major safety finding from a newly completed animal study (such as carcinogenicity),
- Recommendations of the DSMB, if any, where relevant for the safety of subjects.

The sponsor, together with the DSMB if appropriate, decides if the number of events or qualitative changes in the expected SARs comprise a safety issue and must be reported.

8.8 Therapeutic Procedures.

If a patient requires treatment as a result of an Adverse Event, then it must meet the recognized standards of medical care in order to restore the patient's health. Appropriate resuscitation devices and medication must be available in order to treat the patient as quickly as possible in the event of an emergency.

The action taken to treat the AE/SAE must be documented by the investigator either in the appropriate CRF and/or using additional documents.

8.9 Dealing with Pregnancy

In WOCB a pregnancy test will be performed at every visit. There are limited data from the use of Betmiga in pregnant women. Studies in animals have shown reproductive toxicity, but only after application of doses way above the maximum recommended human dose (MHRD). Genotoxicity and carcinogenicity studies have shown no genotoxic or carcinogenic potential in vivo. This is in accordance with the recommendations of the CTFG in case of applying an IMPD with a marketing authorisation and with possible human fetotoxicity (Clinical Trial Facilitation Group (CTFG) 9/15/2014).

Every pregnancy that occurs while taking part in the trial must be reported to the Clinical Trial Centre Leipzig by the investigator/ the deputy or the authorised medical staff within 24 hours of having learned of it.

ZKS Leipzig / Pharmacovigilance
Universität Leipzig
Zentrum für Klinische Studien Leipzig – KKS
Haertelstr. 16-18, 04107 Leipzig
Telefon: +49/341/97-16129
Fax: +49/341/97-16278
E-mail: pharmacovigilance@zks.uni-leipzig.de

Pregnancies have to be reported by using the form „Report on the arising of a pregnancy during exposition to a trial medication". Severe side effects and complications during a pregnancy as well as congenital birth defects are Serious Adverse Events per definition and therefore have to be reported additionally on the Serious Adverse Event form according to the reporting procedures described above.

The outcome of a pregnancy has to be reported on the form "Report on the outcome of a pregnancy during/after exposition to a trial medication". This form will document the outcome of the pregnancy, including a spontaneous or voluntary abortion, details of the birth process, the presence or absence of congenital malformations and birth defects, maternal or foetal complications and the potential relationship to the trial drug.

Collecting data concerning the outcome of a pregnancy is only permitted if the trial subject puts down her permission in writing beforehand.

9 BIOMETRY

9.1 Biometrical Aspects of the Trial Design

This is a prospective, randomised, placebo-controlled, multi-centric international phase IIb parallel-group trial with two groups:

- experimental group, in which patients receive mirabegron for a duration of 12 months after randomisation
- control group, in which patients receive placebo for a duration of 12 months after randomisation

All patients will be followed for 13 months after randomisation.

9.1.1 Measures to Prevent Bias

Randomisation of patients between verum and placebo is performed centrally via a secure web-based tool using a modified minimisation procedure with stochastic component according to Pocock (1983) in a 1:1 proportion.

Randomisation will be balanced according to the following criteria:

- Atrial fibrillation (yes / no)
- Diabetes mellitus (yes / no)
- Trial site

The study is double-blind, and in addition, the two primary endpoints will be assessed centrally in the respective core labs, using standardized protocols.

9.2 End Points

The objective of this trial is to evaluate mirabegron (a new β3-specific agonist) over 12 months as add-on to standard treatment compared to placebo as add-on to standard treatment Endpoints focus on cardiac remodelling: Quantitative indices of hypertrophy and left ventricle (LV) function; in addition exercise tolerance is investigated.

9.2.1 Primary End Points

We define two equally ranked, primary endpoints, in order to assess both structural and functional aspects of left ventricular remodelling:

- Change in left ventricular mass index (LVMI in g/m^2, defined as left ventricular mass divided by body surface) measured at baseline, 6 and 12 months after randomisation. Cardiac MRI is performed locally according to a standardized protocol, and LVMI will be measured in the central MRI core lab. Regression of LV hypertrophy, which is reflected by the LV mass index, is known to be associated with favourable clinical outcomes (Okin et al. 2004).

- Change in diastolic function, assessed as the ratio of peak early transmitral ventricular filling velocity to early diastolic tissue Doppler velocity (E/e’) measured at baseline, 6 and 12 months after randomisation. This parameter will be assessed by echocardiography, performed locally according to a standardized protocol, and will be measured in the central echocardiography core lab. E/e’ is an established indicator of diastolic function, and has been shown to reliably detect changes in functional performance (Whalley et al. 2008; Burgess et al. 2006; Lim et al. 2006; Cahill et al. 2002; Ommen et al. 2000). In addition, change in E/e’ has been shown to be associated with the change in self-reported physical functioning (Edelmann et al. 2011).

The Hochberg method will be used to adjust for endpoint multiplicity (see 9.3).

9.2.2 Secondary End Points

We will evaluate changes of the following secondary endpoints:

- Further MRI endpoints (all measured in the central MRI core lab)
    - Cardiac fibrosis at baseline and at 12 months. Fibrosis is a key pathogenic mechanism of diastolic dysfunction, which is at the origin of HFpEF
    - Left atrial volume index at baseline and at 12 months. This parameter determines diastolic filling (and was shown to predict treatment efficacy in HFpEF in the J-DHF trial (Yamamoto et al. 2013))
    - LV mass index (by cardiac MRI) at 6 months,
    - Diastolic function (E/e') at 6 months;
- Laboratory parameters at baseline and at 3, 6 and 12 months
    - serum biomarkers (Galectin3, GDF15, NT-proBNP, hsTnT)
    - metabolic parameters (fasting glucose, modified HOMA test, HbA1c, serum lipids)
- Maximal exercise capacity (peak VO_2) at baseline and 12 months.
- Safety endpoints
    a. Incidence, severity and frequency of adverse and serious adverse events
    b. Mortality

9.3 Statistical Description of the trial hypothesis

This trial wants to demonstrate that mirabegron as add-on to standard treatment compared to standard treatment alone improves at least one of the two primary endpoints over 12 months.

The null hypotheses are that the 12 month mean changes from baseline mean are identical in both arms. Two-sided tests are used, i.e. the alternative is that mean changes from baseline mean differ by arm.

The Hochberg method will be used to adjust for endpoint multiplicity: If both p-values are below 0.05 we will claim efficacy in both primary endpoints; if otherwise the smallest p-value is below 0.025 we will claim efficacy in the respective primary endpoint. This procedure controls the family-wise error rate (FWER) in the strong sense at a two-sided significance level of 5%. (Hochberg 1988). The Hochberg procedure seems appropriate since we expect non-negatively correlated test-statistics.

9.4 Sample Size Discussion

We investigate two equally ranked, primary endpoints. We will use the Hochberg procedure for multiplicity adjustment in order to control the family-wise error rate at a two-sided significance level of 5%. We conservatively plan sample sizes for a significance level of 2.5%.

We base our sample size calculation on the parameter assessing diastolic function, E/e', since reliable and consistent planning data for this parameter are available in the literature (Edelmann et al. 2013; Kosmala et al. 2011; Edelmann et al. 2011). Typically, E/e' decreases during follow-up in treated patients, while it increases in control patients, leading to mean differences of the baseline-to-follow-up changes of up to 2 between control and treatment group, with a typical baseline mean of about 12.

In our trial, we aim to detect a difference of 1.2 between verum and placebo group. This difference roughly corresponds to 5 points on the SF-36 physical function scale (Edelmann et al. 2011), thus indicating a moderate, but patient-relevant difference. Based on the raw data of the ALDO-DHF and ex-DHF-Pilot trials mentioned above, which were available for additional

analysis, we assume a standard deviation of 3. This is in line with the sample size assumptions *of the DIASTOLE trial (Verloop et al. 2013). With these assumptions, a total of 272 patients have to be analysed to achieve a power of 85% at a significance level of 2.5% using a two-sided t-test (NQuery Advisor ® 7.0).*

The other primary endpoint, left ventricular mass index measured by cardiac MRI, varies markedly in baseline means depending on the patient population studied, e.g.

1. Patients with a history of atherosclerotic events: mean baseline LVMI of about 38±8 g/m^2 , with an estimated standard deviation for changes from baseline to follow-up of about 3.5 (ONTARGET - (Cowan et al. 2009))
2. Patients with resistant hypertension, mean LVMI of about 59±17 g/m$_2$, with an estimated standard deviation for changes from baseline to follow-up of about 8 (Mahfoud et al. 2014).
3. Patients with heart failure with preserved ejection fraction, NYHA II or III, mean LVMI of about 109±27 g/m$_2$, with an estimated standard deviation for changes from baseline to follow-up of about 25 (Edelmann et al. 2013), (Note LVMI was measured by echocardiography in this trial.)

Since there is no data on LVMI in our target population, we cannot fully specify a planning scenario. However, with 272 patients, an effect size in the magnitude of 0.4 is detectable with a power of at least 85% at a significance level of 2.5%. This corresponds to a difference in the change from baseline to follow-up of 1.6 g/m2 if the SD is 4 (in the case of baseline LVMI about 40), of 3.2 g/m2 if the SD is 8 (in the case of baseline LVMI about 60), or of 10 g/m2 if the SD is 25 (in the case of baseline LVMI about 110). Thus we clearly have sufficient power for this endpoint whatever the mean LVMI in our population will turn out to be.

In the trials mentioned above, the drop-out rate was low (ALDO-DHF 5% in 12 months, ex-DHF-Pilot 3% in 6 months, (Kosmala et al. 2011) 1% in 6 months). Thus, we expect a drop-out rate not exceeding 8%. Taking this into account, 296 patients will be randomized.

The BETA3_LVH is a phase IIb trial, and investigates endpoints related to cardiac remodelling. The trial does not address hard clinical endpoints, and is not designed nor powered to detect differences in long-term clinical outcome.

9.5 Statistical Methods

9.5.1 Analysis Population

Full analysis set

The full analysis set (FAS, also called modified intention-to-treat (ITT) population) includes all randomised patients with valid informed consent and at least one valid measurement of the primary endpoints (baseline, 6 months or 12 months).

Per protocol set

The per-protocol (PPS) set is defined by all patients belonging to the ITT without major violations of the study protocol.

The following protocol violations are classified as major:

- Violation of an eligibility criterion;
- Patients who did receive less than 50% of the intended total dose of study medication (mirabegron resp. placebo)
- No valid measurement of the primary endpoints at the 12 month visit.

But patients will be included in the PPS if:

- Study medication had to be interrupted because of medical reasons, e.g. (S)AE, and therefore received lower than 50% of the intended cumulative dose,
- they deceased during the treatment phase

This is not an exclusive list. In the light of protocol violations which actually occur during study conduct, major protocol deviations will be defined in the statistical analysis plan prior to unblinding the data.

Safety analysis set

The safety population is defined by all randomised patients belonging to the FAS who received at least one dose of study medication. Patients will be analysed according to treatment given.

9.5.2 Planned Methods for Analysis

9.5.2.1 Primary endpoints

Confirmatory analysis follows the intention to treat principle as close as possible and will be based on the full analysis set.

In each patient, the primary endpoints will be assessed thrice: at the baseline visit, and at the 6 months (without exercise capacity) and 12 months visits. Analyses of both primary endpoints are identically structured:

Mean changes from baseline mean will be analysed using a repeated measurement linear mixed model without intercept containing the fixed, categorical effects of

- visit (baseline, 6 months, 12 months),
- treatment (verum / placebo),
- treatment by visit interaction
- Atrial fibrillation (yes / no),
- Diabetes mellitus (yes / no),

as well as a patient-specific, visit random effect (3-dimensional normal with a general unstructured variance covariance matrix).
An unstructured co(variance) structure will be used to model the residual within-patient errors. If this analysis fails a compound symmetry structure corresponding to a constant correlation will be used.
The analysis will be based on restricted maximum likelihood (REML). The contrast of interest is the treatment by visit interaction at 12 months. Respective inference will be based on Wald type confidence intervals and p-values.
Unless specificed differently and justified in the statistical analysis plan, the analysis will be implemented in R using the “nlme” package.

This choice of a repeated measurements linear mixed model as the primary analytic model is in line with the recommendations of (Mallincrodt et al. 2008).

As described above, we expect a low rate of patients with missing information on the primary endpoints. We expect that missing endpoints will be missing at random (MAR) given the specified model structure. The above model can deal with patients with incomplete data as long as at least one valid measurement is documented.

Sensitivity analyses (to be specified in the statistical analysis plan) will include

- The above model restricted to the per protocol population,
- ANCOVA with baseline values as covariates and randomisation group as factor
    - In all randomized patients with baseline and 12 months measurements

- with imputation of missing values by last information carried forward (LOCF)

Additional baseline sources of variability will be explored during the blinded review of the data, e.g. age, gender, NYHA class and included in explorative multivariate analyses as appropriate.

9.5.2.2 Secondary endpoints

All other MRI and echocardiographic endpoints as well as peak VO_2 will be analysed along the same lines as the primary endpoints. Time courses of metabolic parameters and specific biomarkers will be described.
Adverse and serious adverse events will be compared by chi-square tests. Odds ratios with 95% confidence intervals will be provided.

No confirmatory subgroup analyses are planned. Exploratory subgroup analyses will include use of a beta-blocker in their standard treatment (yes/no); this is to test the hypothesis that differential regulation of the expression and coupling of the beta3 receptors may occur under beta1 AR blockade. Further exploratory subgroup analyses will be performed with respect to gender.

Safety issues will be carefully monitored. Regular safety analyses will be performed and presented to the DSMB. Statistical monitoring will focus on data quality assurance. All analyses will be pre-specified in a detailed statistical analysis plan, which will be finalised before unblinding the data. The trial will be reported according to CONSORT criteria.

9.6 Statistical Monitoring

The trial conduct will be closely supervised by means of central and statistical monitoring. The objectives are

- to detect safety relevant signals as soon as possible
- to detect non-compliance and relevant protocol violations and to prevent their future occurrence by prompt reaction
- to prevent missing visits or measurements by prompt reminders

Therefore, the following issues will be monitored:

- With regard to safety
  - Patients with treatment discontinuation due to adverse events
  - Serious adverse events
- With regard to protocol compliance
  - Patients with major or minor findings concerning the informed consent documents as detected by on site monitoring
  - Patients with treatment discontinuation
  - Patients with other protocol violations as detected e.g. by on site monitoring
- With regard to missing information
  - Missing values with respect to central MRI and echo measurements
  - Missing values with respect to central laboratory measurements

- Missing visits or visits outside schedule

This is not an exclusive list. Statistical monitoring will be continuously adapted in response to the problems detected.

Statistical and central monitoring will start three months after inclusion of the first patient. The relevant reports and descriptive statistics will be updated and discussed at least monthly at the regular meetings of the ZKS Leipzig study team. Problems and abnormalities will be presented timely to the study coordinator.

9.7 Interim Analysis

No formal interim analysis on efficacy is planned.

9.8 Final Analysis

Final analysis will be performed when the data of all enrolled patients have been collected, all queries have been the resolved and the data base has been closed.

10 CONCOMITANT SCIENTIFIC PROJECTS

Detailed protocols to the scientific side projects will be written as separate documents. This chapter will give an overview on the concomitant side projects only.

10.1 Endothelial Function/Pulse amplitude tonometry and measurement of HbNO

Measurement of peripheral vasodilator response with fingertip Peripheral Arterial Tonometry (PAT) technology (EndoPAT, Itamar Medical, Caesarea, Israel) is emerging as a useful method for assessing vascular function. The PAT device consists of two finger-mounted probes, which include a system of inflatable latex air-cushions within a rigid external case. A blood pressure cuff is placed on one upper arm (study arm), while the contralateral arm serves as a control (control arm) (Lekakis et al. 2011). The reactive hyperaemia Peripheral Arterial Tonometry (RH-PAT) index is calculated as the ratio of the average amplitude of the PAT signal over a 1-min time interval starting 1 min after cuff deflation divided by the average amplitude of the PAT signal of a 3.5-min time period before cuff inflation (baseline) (Lekakis et al. 2011; Bonetti et al. 2004; Ikonomidis et al. 2014). An RHI< 1.35 has been related with impaired coronary endothelial function (Bonetti et al. 2004). Additionally, the PAT device offers the ability to assess arterial wave reflection through measurement of the augmentation index, a validated marker of wave reflections. All studies are stored digitally and analyzed by personnel blinded to clinical and laboratory data, using a computerized station.

The assay of HbNO allows a quantitative measurement of nitric oxide complexed to hemoglobin as a 5-coordinate α-HbNO (HbNO) derivative, in human venous erythrocytes using a new technique developed at FATH/ IREC-UCL. Micro-plethysmography will be performed in the same patients in parallel with ex vivo measurement of HbNO. Protocol of sampling and HbNO quantitation were developed over the last 3 years at FATH/ IREC-UCL; a strong correlation was observed between the erythrocytic HbNO level and endothelial function signal measured by micro-plethysmography (ENDO-PAT) [1]. This HbNO biomarker will provide a quantitative and specific characterization of circulating bioavailable nitric oxide, an important regulator of vascular homeostasis and together with ENDO-PAT measurements, may lead to a better stratification of patients at risk of endothelial dysfunction and HFpEF, beyond traditional cardiovascular risk factors.

Endothelial function and blood sampling for Hb-NO measurements will be done at baseline, 6 and 12 months. Endothelial function will be measured by peripheral (digital) arterial microtonometry (ENDO-PAT) and Hb-NO by quantitative measurement of 5-alpha-coordinated nitrosyl-hemoglobin (Hb-NO) from venous erythrocytes. ENDO-PAT measures post-hyperemic vasodilatation after cuff deflation in the test arm, compared to contralateral, control arm (RHI: Reactive Hyperemia Index, used and validated in numerous intervention trials; (Hedetoft, Olsen 2014); Hb-NO measured in erythrocytes from venous blood by Electron Spin Resonance spectroscopy has been correlated to NO-dependent endothelial function in previous pilot trials (Lobysheva et al. 2013). These parameters will evaluate endothelial function which not only bears on hemodynamic load, but on paracrine signalling in the myocardium, thereby influencing remodelling and diastolic function (Tarone et al. 2014; Knöll et al. 2011). Beta3AR are expressed in human (including coronary) endothelium, where their activation releases nitric oxide (Dessy et al. 2004).

In order to obtain correct Hb-NO measurements pO_2 and pCO_2 from venous blood is needed. Thus, an additional sample of venous blood has to be taken (1 or 2 mL before EndoPat test in line with all blood sampling from the median cubital vein).

10.2 Abundance/activity of brown/beige fat

FDG-PET-Scan for brown fat: measures both metabolic activity (FDG-PET) and volume (CT-scan) of supra-clavicular/thoracic beige/brown fat in standardized (fasting, controlled temperature) conditions at baseline and 12 months. These techniques have identified and quantified brown fat in human adults and demonstrated changes in specific conditions (e.g., hyperthyroidism) (Bauwens et al. 2014; Borga et al. 2014). Brown fat is activated by and expresses beta3-AR, and mediates thermogenic lipolysis, with bearing on metabolism (fat depots, circulating lipids, glucose tolerance). Changes in brown fat under mirabegron will be correlated with metabolic parameters; improvement in the latter may also influence cardiac remodelling (Tarone et al. 2014; Knöll et al. 2011). Minimal dose FDG-PET scan, combined with low-dose CT scan centered on the thoracic region (performed according to a standardized protocol, see protocol in Annex) will be performed at baseline and 12 months. Diabetics (with or without insulin), hyper/hypothyroid patients will be excluded from this sub-study, because of known interference with metabolism and/or FDG uptake. All patients taking beta-blockers will be asked to stop taking this medication for 24 hours before assessment. All measurements will be done in the fasting state (since the previous evening) and in a room with strictly controlled and standardized temperature.

10.3 Analysis of blood and urine samples for scientific purposes related to cardiac disease

During ongoing research in the field of Heart Failure with preserved Ejection Fraction (HFpEF) new markers will be continuously identified, which might be relevant for diagnostics and/or therapy of the disease. To be able to analyse these new candidates in more detail, patients participating in the BETA3_LVH-trial will be asked, if they agree to urine and blood sampling for later scientific sub-projects and if they agree to genetic analyses as part of the informed consent procedure. The samples will be conserved for future research studies targeting gene polymorphisms or metabolic parameters potentially involved in HFpEF and highlighting posteriori correlations between genetic polymorphism/metabolic marker and development of heart failure.

Samples will be drawn at the recruiting centre and labelled exclusively with the trial identification number of the trial participant. Samples will be processed, stored and analysed pseudonymised by solely using the trial identification number.

These samples will be transferred to and stored for 5 years at the Université catholique de Louvain in an anonymised way: only site, trial identification number, date of sampling and type

of sample if applicable (serum or plasma) will be mentioned on the label, to assure the protection and confidentiality of patients' data.

The scientific research making use of the data outside the protocol of the clinical trial will be conducted in accordance with the applicable laws on data protection.

11 ETHICAL, LEGAL AND ADMINISTRATIVE ASPECTS

11.1 GCP-Statement

All persons participating in the conduct of the trial (sponsor, authorized representative of the sponsor, investigators, etc.) commit themselves to observe the Declaration of Helsinki (in its current version, (WMA)), as well as all pertinent national laws and the ICH guidelines for Good Clinical Practice (European Medicines Agency 2006) issued in 2006 and CPMP/ICH/135/95 from September 1997.

11.2 Initial Submission

The trial design together with the available background information and the available safety data on mirabegron has been submitted to the Ethics Committee ("Comité d'Ethique Hospitalo-Facultaire") of Université Catholique de Louvain, and provisional acceptance has been obtained (ref: 2014-11MAR-093).
Following the European legislation, the full trial protocol will be submitted to the competent authorities and responsible ethics committees in all participating countries. The trial will not start before approval has been obtained.

11.2.1 Submission to the Ethics Committee and Competent Authority

Prior to submitting the trial related documents to the leading (and involved) ethics committee(s) and the responsible competent authorities, the sponsor will enter the trial into the European database for clinical trials (EudraCT).

Afterwards, the protocol and all other associated documents according to GCP-V §7 will be submitted to the leading ethics committee for approval. Parallel to the submission to the leading ethics committee (EC), each participating EC is informed of the submission and also receives a copy of the documents including those of the trial sites, which they have to approve. At the same time the study documents will be submitted to the responsible federal authorities according to the requirements of GCP-V §7.

The trial can start only after obtaining a positive review by the leading ethics committee and approval from the responsible federal authorities in each participating country according to the applicable national laws and regulations. The written approval of the ECs and CAs must be filed in the trial master file (TMF). Additionally, every participating centre must receive a copy of these documents to be filed in the investigator site file (ISF).

11.3 Protocol Amendments

Changes made to the protocol that was appraised positively by the ethics committees and approved by the responsible federal authorities must be positively reappraised and approved if the changes

a) are such that they may affect the subjects' safety, (e. g. fundamental changes to the therapeutic procedures)

b) result in further data collection that necessitates changes to the patient information and/or informed consent form,

c) affect the interpretation of the scientific documents upon which the trial is based or the significance of the results of the trial,

d) significantly affect the leadership or conduct of the trial,

e) concern the quality or the innocuousness of the investigational drug, or

Changes to the protocol may only be performed by the co-ordinating investigator in co-operation with the biometrician and DSMB.

After approval of the trial changes, all participating trial sites have to be informed about the changes in writing and supplied with potentially changed documents.

The whole process has to be documented in the Trial Master File (TMF).

12 DOCUMENTATION

12.1 Case Report Forms (CRF)

The SAE form will be provided as paper form. It will be prepared and provided by the Clinical Trial Centre Leipzig in cooperation with the coordinating investigator and printed on 2-part, no carbon required (NCR) paper. The Clinical Trial Centre Leipzig on behalf of the sponsor receives the completed original CRF form. The copy is retained at the trial site.

All other Case report Form (CRF) pages will be designed by the Clinical Trial Centre Leipzig in cooperation with the Coordinating Investigator and DSMB and provided as electronic form (eCRF).

The Investigator or an authorised member of the study team for this task will connect to the database via internet and enter data directly into the database via eCRF data entry masks.

In order to facilitate the documentation as per protocol in case of malfunction of the electronic system or any of its components, a paper version of the CRF will be additionally provided. The content of this paper version will be transferred to the eCRF as soon as the electronic system is available again.

The eCRF will be filled in shortly after each study visit.

Each eCRF page will be signed electronically by the investigator or a therefore authorised member of the study team by assigning the status “completed”. This represents the electronic equivalent of a signature on paper and confirms that all data on the eCRF is correct and hasn’t been changed. If a value gets changed on the eCRF later on, the status “completed” will be set back automatically and has to be assigned again by the investigator or an authorised member of the study team. This ensures that changes on the eCRF will be dated and signed as well. All entries and data changes will be tracked automatically including date, time and person who entered/changed information (audit trail). Major correction or major missing data have to be explained.

If the Investigator authorises other members of the study team to enter and sign CRF data, their name, initials, position, signature must be supplied to the Sponsor or its authorised representative via Staff Signature und Delegation Log.

However, at all times the **investigator has final responsibility** for the accuracy and authenticity of all clinical and laboratory data entered in the CRF.

An eCRF will be provided for each patient. The patient will be identified as per the Patient-ID only. All information required by the protocol and therefore collected during the clinical trial must be recorded by the Investigator or an authorised member of the study team as source data in the source documentation for the study.

Source data according to ICH-GCP E6 are defined as any information in original records and certified copies of original records of clinical findings, observations, or other activities in a clinical trial necessary for the reconstruction and evaluation of the trial. Source data are contained in source documents (original records or certified copies).

It is defined for each trial site, what source data for respective CRF entries are and where these are filed (Source Data Agreement).

12.2 Data Management

For creation of the study database the EDC Tool eData Entry (eDE) by OmniCom or an equivalent professional Clinical Data Management Software (CDMS) will be used. The database will be validated according to the Standard Operating Procedures (SOPs) of the Clinical Trial Centre Leipzig prior to data capture.

The information entered into the eCRF by the investigator or an authorised member of the study team is systematically checked for completeness, consistency and plausibility by routines implemented in the CDMS, running every night. Error messages generated by these routines will be checked by data management staff of the Clinical Trial Centre Leipzig.

Discrepancies, errors or omissions will be passed to the investigator or an authorised member of the study team at the investigational site by the query management tool of eDE. The investigator will receive notification of all queries concerning his/her investigational site. The Clinical Trial Centre Leipzig will supervise and support the solution of the queries. Corrected data will be re-checked by automatic routines during the night after entry. In case a query cannot be solved, the Data Management staff of the Clinical Trial Centre Leipzig may close the query. This shall happen in agreement with the study biometrician and clarification, if the information addressed by the query is relevant for the results of the study, or not.

The access concept for the trial database will be based on a strict hierarchy and role model. Thus, data access is limited to authorized persons only, and unauthorised access to pseudonymised patient data is prevented. Any change of data (e.g. when data is changed in the database during query management) is recorded automatically via audit trail within the database. At the end of the study, once the database has been declared complete and accurate, the database will be locked. Thereafter, any changes to the database are possible only by joint written agreement between coordinating investigator, biometrician and data manager.

During the whole course of the study, a backup all data is made on a daily basis. Unauthorised access to patient data is prevented by the access concept of the study database which is based on a strict hierarchy and role model. Any change of data (e.g. when data is changed in the database during query management) is recorded automatically via audit trail within the database.

At the end of the study, once the database has been declared complete and accurate, the database will be locked. Thereafter, any changes to the database are possible only by joint written agreement between coordinating investigator, biometrician and data manager.

12.3 Archiving

All relevant trial documentation (Trial Master File), the electronically stored data, the original CRFs and the final report will be stored for 20 years by the sponsor after the trial’s completion.

At the investigating sites, the investigators’ files, patient identification lists, signed written consent forms, copies of all CRFs and the patients’ files will be stored for 10 years after the trial’s completion. If local rules or other legal requirements (e.g. radiation protection regulatories) require longer periods of archiving, these will be met.

13 SUPERVISION OF THE CLINICAL TRIAL

13.1 Access to Source Data

According to ICH-GCP, the investigator must permit all authorized third parties access to the trial site and the medical records of the trial subjects (source data). These include the clinical trial monitors, auditors and other authorized employees of the sponsor, as well as members of the local or federal authorities. All these persons are sworn to secrecy.

13.2 Monitoring

Monitoring is mandatory for all participating study groups. Monitoring procedures will be country specific and as agreed by the Sponsor and the individual regional offices. General principles for monitoring will be outlined in the Monitoring Manuals for those regional offices.

Central and biometrical monitoring procedures will be combined with on-site monitoring visits in order to achieve high protocol compliance and data quality, as well as to ensure patients' safety and rights.

A risk-based monitoring strategy will be implemented, using the risk-based approach proposed by the ADAMON project group (Brosteanu et al. 2009). According to ADAMON risk analysis, treatment delivery parameters, adverse events, follow-up information, data transmission and protection and informed consent documents comprise risk-bearing trial aspects and will be monitored.

Prior to recruitment, each participating centre will receive a site initiation visit, during which a Sponsor representative will review the protocol and the eCRFs with centre staff and provide any necessary training. During the study, trial monitors will maintain regular contact with trial centre personnel (by telephone/fax/email/post) to track the progress of the trial, respond to any problems, and provide general assistance and support.

The first regular monitoring visit at a site will be scheduled after the inclusion of the site's first patient to check protocol compliance and to prevent further systematic errors due to misunderstandings. All trial sites will be visited on a regular basis. The frequency of monitoring visits will depend on the trial site's recruitment rate and on whether problems have been detected during previous on-site visits or by central monitoring.

Prior to every scheduled on-site visit, the monitor will receive summaries of the site`s patient data already documented in the database, and if applicable with data indicating possible protocol deviations or inconsistencies. During the visits, the monitor will

- check informed consent forms of all patients enrolled
- perform source data verification of the key data (selected baseline parameters, therapy delivery, toxicities, serious adverse events, follow-up) in a random sample of at least 20% of the site's patients
- perform targeted source data verification for patients with possible deviations
- random testing of drug accountability
- discuss open queries raised by data management or drug safety personnel
- check essential parts of the investigator site file (see Monitoring Manual)
- check source data for AEs or SAEs, which have not been properly reported in the CRF/eCRF
- check for major GCP-breaches and/or protocol violations

13.3 Audits

In order to guarantee that the conduct of the study is in accordance with ICH-GCP and the national laws, the sponsor may initiate for cause or random audits at the trial sites, core labs or involved institutions to be carried out by an unbiased auditor.

The investigator agrees to give the auditor access to all relevant documents for review.

13.4 Inspections

According to the GCP-guidelines (GCP-V), inspections of the trial sites may be performed by the local or federal authorities at any time during or after completion of the trial.

The investigator agrees to give the inspectors access to all relevant documents for review.

13.5 Independent Supervision of the Trial

13.5.1 Data Safety and Monitoring Board (DSMB)

An Independent Data Safety and Monitoring Board (DSMB) will be set-up and will meet periodically to review the results of the clinical trial, to evaluate any safety or efficacy issues that may arise during the course of the study and to advise the study investigators on the required course of action.

The DSMB will consist of three individual experts who are not involved in the BETA3_LVH clinical trial activities and who have no conflict of interest (financial, proprietary, professional or other) with any of the participating organisations.

These core members will be chosen to have sufficient combined expertise in the medical disciplines at hand:

- The clinical aspects of the HFpEF disease, specifically in an ageing population and discerning the differences between men and women
- Biostatistics
- Clinical trial conduct and methodology

Other ad hoc specialists may be invited as a non-voting member of the DSMB whenever additional expertise is required.

Before the start of the clinical trial, the DSMB will review the clinical protocol for any major concern. During the trial, the DSMB will perform a periodic review and evaluation of the accumulated study data, in order to control the participant safety, appropriate study conduct, study progress, scientific validity and integrity of the trial. In order to allow them to do so, the safety reports will be submitted to the DSMB on a quarterly basis. While reviewing, the DSMB will consider the study-specific data as well as any relevant background knowledge of the HFpEF disease, the tested drug mirabegron and the information provided about the patient population in the study. The DSMB will specifically review:

- The quality, completeness, accuracy and timeliness of the collected data
- The collected data so far that may provide evidence of study-related adverse effects
- The performance of the individual clinical centres that are involved in the study
- The overall compliance with the study protocol and the goals for recruitment and retention (including related gender issues)

- All factors internal or external to the study that may affect the study outcome, compromise the confidentiality or the ethics of the study or impact patient safety (protocol violations, data unmasking, newly available scientific or therapeutic developments…)

The DSMB will meet at regular intervals (four times a year, preferably by telephone, but in person if needed) in open sessions (for issues relating to the general conduct and progress of the study) or in closed sessions (when reviewing coded grouped safety data). The DSMB will maintain the data confidentiality during all phases of the reviews and deliberations. Only when it is determined that the identity of the patient groups are necessary for the decision-making of the DSMB, the data will be unmasked before reviewing and only voting members will be present to review the data.

Every 3 months, the DSMB will provide written recommendations to the Trial Steering Committee on the continuation, modification or termination of the clinical trial. Such recommendations can be based on the detection of emerging negative data trends or prospects of ethical or safety guidelines not being met. Contacts between the DSMB and the Trial Steering Committee by teleconferencing can also be requested by the DSMB.

The DSMB will be supported by an independent team of statisticians. This team will have regular access to the safety and trial conduct data accumulated during the trial, as well as to the randomisation code. They will regularly liaise with the Clinical Trial Centre Leipzig, who will provide updated data in appropriate format for statistical analysis. The statistical team will produce regular (quarterly) safety reports on the trial. They will also independently replicate the final statistical analysis, to ensure credible results at the end of the trial.

13.5.2 Event Adjudication Committee (EAC)

An Event Adjudication Committee (EAC) will be set up, consisting of 3 external expert cardiologists who will analyse Serious and Adverse Events (SAE) from the trial. An "Adjudication form" will be developed in coordination with UNI-Leipzig, in order to facilitate the collection of all the relevant data to be reported when a SAE occurs. As a guideline, the minimal set of criteria for SAE reporting as defined in Eudravigilance guidance document CT-3, 2011/C 172/01, Pt. 97 will be used.

The EAC will liaise with the data manager (UNI-Leipzig) to assemble all data on SAE and put them in a format that can be analysed by the experts. The EAC will analyse all the events data at the end of trial and duly adjudicate. A summary meeting will be held by teleconference after which a final report will be produced and sent to the Trial Manager (UNI-Leipzig).

14 DATA PROTECTION AND CONFIDENTIALITY

The Clinical Trial Centre Leipzig will be responsible, on behalf of the legal trial sponsor Université catholique de Louvain, for implementation of procedures for data collection, storage, protection, retention and destruction. The Clinical Trial Centre Leipzig has implemented a data safety and security concept according to the requirements of the German Federal Office for Information Security (www.bsi.bund.de). All BETA3_LVH related procedures will be developed in cooperation with the data security engineer of Clinical Trial Centre Leipzig (Matthias Collier, matthias.collier@zks.uni-leipzig.de), and have to be approved by the official data protection officer of UNI-Leipzig (Thomas Braatz, Universität Leipzig, Datenschutzbeauftragter Hochschulbereich/Medizinische Fakultät, dsb@uni-leipzig.de) prior to implementation.

Two types of data will be collected in the BETA3_LVH trial: clinical data (including patient demographics, medical conditions, therapy related information, course of disease, clinical outcomes) and imaging data (MRI, echocardiography). All data will be initially collected by investigators in the recruiting trial centres. Together with information on the trial, eligible patients will be informed about data capture, transmission and analysis processes. Once a patient is eligible and has given his/her informed consent to trial participation and data collection, the investigator will assign the patient a unique patient identification code. Patient identification code lists will be generated in advance by Clinical Trial Centre Leipzig and

forwarded to the recruiting centres. These lists are part of the investigator site file and remain at the recruiting site. These lists are the only documents that allow for re-identification of the patients.

All clinical data will be entered by the investigators (or their designated staff) into eCRFs (electronic case report forms). Patient data will be recorded in pseudonymised form (i.e. without reference to the patient's name) using the patient's identification code.

Imaging data for reference evaluation of the MRIs and echocardiography will be exchanged between the institutions using an “ownCloud” file-hosting system, which is hosted on a server of the UNI-Leipzig (Clinical Trial Centre) and underlies the same hierarchical role concept as the trial database. Data will be uploaded without personal information, using exclusively the trial identification number. Trial centres will only be able to upload data and see data concerning their own patients, while the reference organisation may exclusively download data essential for their evaluation. An access concept for the trial database will be implemented, based on a strict hierarchy and role model. Thus, data access is limited to authorized persons only, and unauthorised access to pseudonymised patient data is prevented. Data are protected against unauthorized access.

Using an eCRF as well as the “ownCloud” file-hosting system, both located on servers of the UNI-Leipzig (Clinical Trial Centre) and thus behind the firewall of University of Leipzig, reduces the risk of unauthorised or unlawful access, disclosure, dissemination, alteration, destruction or accidental loss in comparison to data transmission over a network. Access to the servers is secured via https protocol, and requires user-specific login and password.

Additionally, urine and blood sampling for later scientific sub-projects is planned to be able to analyse so far unknown markers of e.g. diagnostic, therapeutic or prognostic value, once they become known. Samples will be drawn at the recruiting centre and labelled exclusively with the trial identification number of the trial participant. Samples will be processed, stored and analysed pseudonymised by solely using the trial identification number. The scientific research making use of the data outside the protocol of the clinical trial will be conducted in accordance with the applicable law on data protection and the patient will explicitly be asked for his consent on participation in the scientific projects and pseudonymised storage and use of his/her samples (see below).

Clinical monitors appointed by Clinical Trial Centre Leipzig (for Germany) and ECRIN (for all other countries) will regularly visit the recruiting centres and verify the informed consent forms. Data will only be used for analysis after it has been verified by monitors that the patient has unambiguously given his or her consent for trial participation as well as for data capture, transmission and analysis.

In the event of withdrawal of consent, the necessity for storing data and/or samples will be evaluated. While Regulation (EC) No 45/2001 of the European Parliament and of the Council strengthen personal data protection rights, encompassing the right to access, rectification and withdrawal of data, it also specifies the situations when restriction on those rights may be imposed. The withdrawal of informed consent should not affect the results of activities already carried out, such as the storage and use of data obtained on the basis of informed consent before. Data not needed will be deleted as requested, with full documentation of the reasons for deletion. Accordingly samples will be discarded as wished.

After reaching the study aim/after finishing of all concomitant scientific projects, personal data will be stored in an anonymous manner for 10 years, if there are no other regulatory or contractual time periods for archiving.

Since in the course of the trial contact between the trial centre and the patients might be necessary, the patients' full name, address and telephone number will be ascertained and stored after obtaining written permission to do so. This information will be stored separately from the trial data.

14.1 Declaration regarding Data Protection

We hereby confirm that all clinical trial information will be recorded, processed, handled, and stored by Clinical Trial Centre Leipzig, Härtelstr. 16-18, 04107 Leipzig, Germany on behalf of the sponsor, by the reference centres and by the investigators, in such a way that it can be accurately reported, interpreted and verified while the confidentiality of records and the personal data of the subjects remain protected in accordance with the applicable law on personal data protection and in accordance with Directive 95/46/EEC and regulation (EC) No 45/2001 of the European parliament and of the council.

14.2 Declaration regarding the Pseudonymized Transfer of Personal Data

The sponsor certifies herewith that the transfer of pseudonymized personal data will take place according to the documentation and communication regulations in §§12 und 13 of the GCP-guidelines. Moreover, the sponsor certifies that trial participants who do not permit the transfer of data will not be admitted to the trial.

15 ADMINISTRATIVE AGREEMENTS

15.1 Adherence to the Protocol

The clinical trial described here will be conducted and analysed in accordance with local laws and ICH guidelines for Good Clinical Practice (GCP).

Protocol violations are all deviations from the procedures outlined in this document, e.g.

- examinations that are missed or that take place at the wrong time
- non-compliance (with the protocol, SOPs, GCP and /or applicable regulatory requirements) by an investigator/institution
- intake of prohibited medication
- mistakes in the informed consent procedure

After a patient has been enrolled, it is the investigator's responsibility to avoid protocol violations in order to obtain unbiased data for the trial.

Those protocol violations that are to be deemed major are listed in section 9.5.1 and will be further detailed in a separate document belonging to the risk assessment performed before trial start. This list can be augmented in the course of the trial. Major protocol violations will be reported to CTC Leipzig who will inform the coordinating investigator who will report to the ethic committee.

All protocol violations will be documented and discussed with the responsible biometrician before closing the data bank and carrying out the statistical analysis. This is done without revealing the treatment allocation of the patient.

The investigator must ensure that the recorded data are documented as per protocol. Minor variations are inevitable, but must be documented and justified.

In the UK serious breaches have to be reported to the MHRA GCP Inspectorate within 7 days of the Sponsor becoming aware of the breach. This will be done using the Serious Breaches Notification Form of the MHRA.

15.2 Funding and Insurance

This trial is funded by the European Union’s Horizon 2020 research and innovation program under grant agreement N° 634559.

The authorised institutions of all participating countries are responsible for the provision of insurance or indemnity to cover the liability of the investigator and the sponsor in the respective

country (if required by national law), as required by the Directive 2001/20/EC of the European Parliament and of the Council of 4 April 2001 and the corresponding national laws.

Details will be specified with the application to the ethics committees in each country.

At each trial site, a copy of the policy and the general policy conditions will be filed in the ISF, if applicable. The patient will receive a copy of the general policy conditions upon request.

15.3 Notification of the Local Authorities (Germany only)

Prior to enrolment of the first patient in the trial, the sponsor, his/her legal representatives/ contractors and all investigators and their deputies are responsible according to German drug law AMG §67 (1) and the requirements of the GCP-V §12 and 13 for notifying the local regulatory authority of their participation in the trial.

According to §67 (3) AMG and §§ 12,13 GCP-V the sponsor, his/her legal representatives/ contractors and all investigators and their deputies are also responsible for notifying the local regulatory authority of amendments, premature termination of trial arms or of the whole study and the regular trial termination.

15.4 Publication Policy and Registration

The results of this trial will be submitted for publication in a peer-reviewed, international english-language journal of appropriate aim and scope. Accordingly, the clinical trial will be registered at clinicaltrials.gov and in the ISRCTN register before recruitment starts. According to the results of main and concomitant studies, the results may be submitted in separate or combined manuscripts; decisions about the form and scope of individual manuscripts will be discussed among all persons participating in the design, conduct and analysis of the study who qualify for authorship. The coordinating investigator together with the biometrician(s) is responsible for drafting and circulating manuscripts and for discussing and handling requests by co-authors or/and sponsors to edit the text.

The authorship will follow the criteria for authorship developed by the International Committee of Medical Journal Editors (ICMJE), including those that distinguish authors from other contributors.

The ICMJE recommends that authorship be based on the following 4 criteria:

- Substantial contributions to the conception or design of the work; or the acquisition, analysis, or interpretation of data for the work; AND
- Drafting the work or revising it critically for important intellectual content; AND
- Final approval of the version to be published; AND
- Agreement to be accountable for all aspects of the work in ensuring that questions related to the accuracy or integrity of any part of the work are appropriately investigated and resolved.

All those designated as authors should meet all four criteria for authorship, and all who meet the four criteria should be identified as authors. Those who do not meet all four criteria will be acknowledged in the manuscript.

The scientific use of data resulting from this trial by local trial sites is ruled by the site contracts between the sponsor and the local trial sites. Generally, sites might use data for own scientific questions (independent from the questions discussed in this trial protocol) and publication after consultation with the sponsor.

16 REFERENCES

Publication bibliography

Abraham, William T.; Fisher, Westby G.; Smith, Andrew L.; Delurgio, David B.; Leon, Angel R.; Loh, Evan et al. (2002): Cardiac resynchronization in chronic heart failure. In: *The New England journal of medicine* 346 (24), S. 1845–1853. DOI: 10.1056/NEJMoa013168.

Baba, Shingo; Jacene, Heather A.; Engles, James M.; Honda, Hiroshi; Wahl, Richard L. (2010): CT Hounsfield units of brown adipose tissue increase with activation: preclinical and clinical studies. In: *Journal of nuclear medicine : official publication, Society of Nuclear Medicine* 51 (2), S. 246–250. DOI: 10.2967/jnumed.109.068775.

Baulmann, Johannes; Schillings, Ulrich; Rickert, Susanna; Uen, Sakir; Düsing, Rainer; Illyes, Miklos et al. (2008): A new oscillometric method for assessment of arterial stiffness: comparison with tonometric and piezo-electronic methods. In: *Journal of hypertension* 26 (3), S. 523–528. DOI: 10.1097/HJH.0b013e3282f314f7.

Bauwens, Matthias; Wierts, Roel; van Royen, Bart; Bucerius, Jan; Backes, Walter; Mottaghy, Felix; Brans, Boudewijn (2014): Molecular imaging of brown adipose tissue in health and disease. In: *European journal of nuclear medicine and molecular imaging* 41 (4), S. 776–791. DOI: 10.1007/s00259-013-2611-8.

Belge, Catharina; Hammond, Joanna; Dubois-Deruy, Emilie; Manoury, Boris; Hamelet, Julien; Beauloye, Christophe et al. (2014): Enhanced expression of β3-adrenoceptors in cardiac myocytes attenuates neurohormone-induced hypertrophic remodeling through nitric oxide synthase. In: *Circulation* 129 (4), S. 451–462. DOI: 10.1161/CIRCULATIONAHA.113.004940.

Boellaard, Ronald; O'Doherty, Mike J.; Weber, Wolfgang A.; Mottaghy, Felix M.; Lonsdale, Markus N.; Stroobants, Sigrid G. et al. (2010): FDG PET and PET/CT: EANM procedure guidelines for tumour PET imaging: version 1.0. In: *European journal of nuclear medicine and molecular imaging* 37 (1), S. 181–200. DOI: 10.1007/s00259-009-1297-4.

Bonetti, Piero O.; Pumper, Geralyn M.; Higano, Stuart T.; Holmes, David R.; Kuvin, Jeffrey T.; Lerman, Amir (2004): Noninvasive identification of patients with early coronary atherosclerosis by assessment of digital reactive hyperemia. In: *Journal of the American College of Cardiology* 44 (11), S. 2137–2141. DOI: 10.1016/j.jacc.2004.08.062.

Borga, Magnus; Virtanen, Kirsi A.; Romu, Thobias; Leinhard, Olof Dahlqvist; Persson, Anders; Nuutila, Pirjo; Enerbäck, Sven (2014): Brown adipose tissue in humans: detection and functional analysis using PET (positron emission tomography), MRI (magnetic resonance imaging), and DECT (dual energy computed tomography). In: *Methods in enzymology* 537, S. 141–159. DOI: 10.1016/B978-0-12-411619-1.00008-2.

Brosteanu, Oana; Houben, Peggy; Ihrig, Kristina; Ohmann, Christian; Paulus, Ursula; Pfistner, Beate et al. (2009): Risk analysis and risk adapted on-site monitoring in noncommercial clinical trials. In: *Clinical trials (London, England)* 6 (6), S. 585–596. DOI: 10.1177/1740774509347398.

Bundgaard, Henning; Axelsson, Anna (2016): The-First-in-Man Randomized Trial of a BETA3-adrenoceptor Agonist in Chronic Heart Failure - The BEAT-HF Trial. Online verfügbar unter http://www.abstractsonline.com/pp8/#!/3795/presentation/49208, zuletzt aktualisiert am 08.06.2016, zuletzt geprüft am 14.07.2016.

Bundgaard, Henning; Liu, Chia-Chi; Garcia, Alvaro; Hamilton, Elisha J.; Huang, Yifei; Chia, Karin K M et al. (2010): β(3) adrenergic stimulation of the cardiac Na+-K+ pump by reversal of an inhibitory oxidative modification. In: *Circulation* 122 (25), S. 2699–2708. DOI: 10.1161/CIRCULATIONAHA.110.964619.

Burgess, Malcolm I.; Jenkins, Carly; Sharman, James E.; Marwick, Thomas H. (2006): Diastolic stress echocardiography: hemodynamic validation and clinical significance of estimation of ventricular filling pressure with exercise. In: *Journal of the American College of Cardiology* 47 (9), S. 1891–1900. DOI: 10.1016/j.jacc.2006.02.042.

Busko, Marlene (2015): Beta-3 Agonist Mirabegron May Help in Severe HF, BEAT HF Hints. Online verfügbar unter http://www.medscape.com/viewarticle/854954, zuletzt aktualisiert am 24.11.2015, zuletzt geprüft am 14.07.2016.

Cahill, John M.; Horan, Mairead; Quigley, Peter; Maurer, Brian; McDonald, Ken (2002): Doppler-echocardiographic indices of diastolic function in heart failure admissions with preserved left ventricular systolic function. In: *European journal of heart failure* 4 (4), S. 473–478.

Camm, A. John; Kirchhof, Paulus; Lip, Gregory Y. H.; Schotten, Ulrich; Savelieva, Irene; Ernst, Sabine et al. (2010): Guidelines for the management of atrial fibrillation: the Task Force for the Management of Atrial Fibrillation of the European Society of Cardiology (ESC). In: *European heart journal* 31 (19), S. 2369–2429. DOI: 10.1093/eurheartj/ehq278.

Center for Drug Evaluation and Research: Drug Interactions & Labeling - Drug Development and Drug Interactions: Table of Substrates, Inhibitors and Inducers. Center for Drug Evaluation and Research. Online verfügbar unter http://www.fda.gov/Drugs/DevelopmentApprovalProcess/DevelopmentResources/DrugInteractionsLabeling/ucm093664.htm#major, zuletzt geprüft am 19.01.2016.

Chapple, Christopher R.; Cardozo, Linda; Nitti, Victor W.; Siddiqui, Emad; Michel, Martin C. (2014): Mirabegron in overactive bladder: a review of efficacy, safety, and tolerability. In: *Neurourology and urodynamics* 33 (1), S. 17–30. DOI: 10.1002/nau.22505.

CHMP/ICH: E 14 The Clinical Evaluation of QT/QTc Interval Prolongation and Proarrhythmic Potentialfor Non-Antiarrhythmic Drugs. Online verfügbar unter http://www.ema.europa.eu/docs/en_GB/document_library/Scientific_guideline/2009/09/WC500002879.pdf, zuletzt geprüft am 04.05.2016.

CHMP/ICH: E 14 The Clinical Evaluation of QT/QTc Interval Prolongation and Proarrhythmic Potentialfor Non-Antiarrhythmic Drugs. Online verfügbar unter http://www.ema.europa.eu/docs/en_GB/document_library/Scientific_guideline/2009/09/WC500002879.pdf, zuletzt geprüft am 19.01.2016.

Chugh, Sumeet S.; Blackshera Joseph L.; Shen Win-Kuang; Hammill Stephen C.; Gersh Bernhard J. (2001): Epidemiology and natural history of atrial fibrillation: clinical implications. In: *JACC. Cardiovascular imaging* (Vol. 37, No.2), S. 371–378, zuletzt geprüft am 11.01.2016.

Clinical Trial Facilitation Group (CTFG) (15.09.2014): Recommendations related to contraception and pregnancy testing in clinical trials, S. 1–13. Online verfügbar unter http://www.hma.eu/fileadmin/dateien/Human_Medicines/01-About_HMA/Working_Groups/CTFG/2014_09_HMA_CTFG_Contraception.pdf, zuletzt geprüft am 12.01.2016.

Cohn J. N.; Ferrari R.; Sharpe N. (2000): Cardiac remodeling—concepts and clinical implications: a consensus paper from an international forum on cardiac remodeling. In: *Journal of the American College of Cardiology* 35 (3), S. 569–582, zuletzt geprüft am 21.01.2016.

Colish, Jane; Walker, Jonathan R.; Elmayergi, Nader; Almutairi, Saleh; Alharbi, Fawaz; Lytwyn, Matthew et al. (2012): Obstructive sleep apnea: effects of continuous positive airway pressure on cardiac remodeling as assessed by cardiac biomarkers, echocardiography, and cardiac MRI. In: *Chest* 141 (3), S. 674–681. DOI: 10.1378/chest.11-0615.

Committee for Medicinal Products for Human Use (CHMP) (2012): Betmiga, INN-mirabegron. Assessment report. Hg. v. European Medicines Agency. Online verfügbar unter http://www.ema.europa.eu/docs/en_GB/document_library/EPAR_-_Public_assessment_report/human/002388/WC500137308.pdf, zuletzt geprüft am 18.01.2016.

Conrath, C. E.; Wilde, A. A. M.; Jongbloed, R. J. E.; Alders, M.; van Langen, I. M.; van Tintelen, J. P. et al. (2002): Gender differences in long QT syndrome: effects of ß-adrenoceptor blockade. In: *Cardiovascular Research* 53, S. 770–776. Online verfügbar unter http://cardiovascres.oxfordjournals.org/content/cardiovascres/53/3/770.full.pdf, zuletzt geprüft am 19.01.2016.

Cowan, Brett R.; Young, Alistair A.; Anderson, Craig; Doughty, Robert N.; Krittayaphong, Rungroj; Lonn, Eva et al. (2009): Left ventricular mass and volume with telmisartan, ramipril, or combination in patients with previous atherosclerotic events or with diabetes mellitus (from the ONgoing Telmisartan Alone and in Combination With Ramipril Global Endpoint Trial [ONTARGET]). In: *The American journal of cardiology* 104 (11), S. 1484–1489. DOI: 10.1016/j.amjcard.2009.07.018.

Cypess, Aaron M.; Chen, Yih-Chieh; Sze, Cathy; Wang, Ke; English, Jeffrey; Chan, Onyee et al. (2012): Cold but not sympathomimetics activates human brown adipose tissue in vivo. In: *Proceedings of the National Academy of Sciences of the United States of America* 109 (25), S. 10001–10005. DOI: 10.1073/pnas.1207911109.

Cypess, Aaron M.; Weiner, Lauren S.; Roberts-Toler, Carla; Franquet Elía, Elisa; Kessler, Skyler H.; Kahn, Peter A. et al. (2015): Activation of human brown adipose tissue by a β3-adrenergic receptor agonist. In: *Cell metabolism* 21 (1), S. 33–38. DOI: 10.1016/j.cmet.2014.12.009.

Dahlöf, Björn; Devereux, Richard B.; Kjeldsen, Sverre E.; Julius, Stevo; Beevers, Gareth; Faire, Ulf de et al. (2002): Cardiovascular morbidity and mortality in the Losartan Intervention For Endpoint reduction in hypertension study (LIFE). A randomised trial against atenolol. In: *The Lancet* 359 (9311), S. 995–1003. DOI: 10.1016/S0140-6736(02)08089-3.

DCTD, NCI, NIH, DHHS (2003): Cancer Therapy Evaluation Program, Common Terminology Criteria for Adverse Events, Version 3.0, 31.03.2003. Online verfügbar unter http://ctep.cancer.gov.

Dessy, C.; Moniotte, S.; Ghisdal, P.; Havaux, X.; Noirhomme, P.; Balligand, J. L. (2004): Endothelial beta3-adrenoceptors mediate vasorelaxation of human coronary microarteries through nitric oxide and endothelium-dependent hyperpolarization. In: *Circulation* 110 (8), S. 948–954. DOI: 10.1161/01.CIR.0000139331.85766.AF.

Dessy, Chantal; Saliez, Julie; Ghisdal, Philippe; Daneau, Géraldine; Lobysheva, Irina I.; Frérart, Françoise et al. (2005): Endothelial beta3-adrenoreceptors mediate nitric oxide-dependent vasorelaxation of coronary microvessels in response to the third-generation beta-blocker nebivolol. In: *Circulation* 112 (8), S. 1198–1205. DOI: 10.1161/CIRCULATIONAHA.104.532960.

Devereux, R. B.; Reichek, N. (1977): Echocardiographic determination of left ventricular mass in man. Anatomic validation of the method. In: *Circulation* 55 (4), S. 613–618.

Dixon, Brian J.; Bracha, Yiscah; Loecke, Steven W.; Guerrero, Marco A.; Taylor, Richard D.; Asinger, Richard W. (2005): Principal atrial fibrillation discharges by the new ACC/AHA/ESC classification. In: *Archives of internal medicine* 165 (16), S. 1877–1881. DOI: 10.1001/archinte.165.16.1877.

Dodson, John A.; Neilan, Tomas G.; Shah, Ravi V.; Farhad, Hoshang; Blankstein, Ron; Steigner, Michael et al. (2014): Left atrial passive emptying function determined by cardiac magnetic resonance predicts atrial fibrillation recurrence after pulmonary vein isolation. In: *Circulation. Cardiovascular imaging* 7 (4), S. 586–592. DOI: 10.1161/CIRCIMAGING.113.001472.

Douglas, Pamela S.; DeCara, Jeanne M.; Devereux, Richard B.; Duckworth, Shelly; Gardin, Julius M.; Jaber, Wael A. et al. (2009): Echocardiographic imaging in clinical trials: American Society of Echocardiography Standards for echocardiography core laboratories: endorsed by the American College of Cardiology Foundation. In: *Journal of the American Society of Echocardiography : official publication of the American Society of Echocardiography* 22 (7), S. 755–765. DOI: 10.1016/j.echo.2009.05.020.

Edelmann, Frank; Gelbrich, Götz; Düngen, Hans-Dirk; Fröhling, Stefan; Wachter, Rolf; Stahrenberg, Raoul et al. (2011): Exercise training improves exercise capacity and diastolic function in patients with heart failure with preserved ejection fraction: results of the Ex-DHF (Exercise training in Diastolic Heart Failure) pilot study. In: *Journal of the American College of Cardiology* 58 (17), S. 1780–1791. DOI: 10.1016/j.jacc.2011.06.054.

Edelmann, Frank; Wachter, Rolf; Schmidt, Albrecht G.; Kraigher-Krainer, Elisabeth; Colantonio, Caterina; Kamke, Wolfram et al. (2013): Effect of spironolactone on diastolic function and exercise capacity in patients with heart failure with preserved ejection fraction: the Aldo-DHF randomized controlled trial. In: *JAMA* 309 (8), S. 781–791. DOI: 10.1001/jama.2013.905.

European Commission (03.02.2010): EU Guidelines to Good Manufacturing Practice. Medicinal Products for Human and Veterinary Use, Annex 13, zuletzt geprüft am 14.07.2015.

European Commission (2004.04):: Detailed guidance on the collection, verification and presentation of adverse reaction reports arising from clinical trials on medicinal products for human use, revision 2.

European Medicines Agency (2006): ICH Topic E6 (R1) - Guideline for Good Clinical Practice. Online verfügbar unter http://ethikkommission.meduniwien.ac.at/fileadmin/ethik/media/dokumente/rechtsgrundlagen/GCP.pdf, zuletzt geprüft am 06.10.2015.

Evans, B. A.; Sato, M.; Sarwar, M.; Hutchinson, D. S.; Summers, R. J. (2010): Molecular pharmacology of G protein-coupled receptors. Editorial. In: *British journal of pharmacology* 159 (5), S. 983–985. DOI: 10.1111/j.1476-5381.2010.00695.x.

Ferreira, Vanessa M.; Piechnik, Stefan K.; Dall'Armellina, Erica; Karamitsos, Theodoros D.; Francis, Jane M.; Choudhury, Robin P. et al. (2012): Non-contrast T1-mapping detects acute myocardial edema with high diagnostic accuracy: a comparison to T2-weighted cardiovascular magnetic resonance. In: *Journal of cardiovascular magnetic resonance : official journal of the Society for Cardiovascular Magnetic Resonance* 14, S. 42. DOI: 10.1186/1532-429X-14-42.

Flett, Andrew S.; Hasleton, Jonathan; Cook, Christopher; Hausenloy, Derek; Quarta, Giovanni; Ariti, Cono et al. (2011): Evaluation of techniques for the quantification of myocardial scar of differing etiology using cardiac magnetic resonance. In: *JACC. Cardiovascular imaging* 4 (2), S. 150–156. DOI: 10.1016/j.jcmg.2010.11.015.

Flett, Andrew S.; Hayward, Martin P.; Ashworth, Michael T.; Hansen, Michael S.; Taylor, Andrew M.; Elliott, Perry M. et al. (2010): Equilibrium contrast cardiovascular magnetic resonance for the measurement of diffuse myocardial fibrosis: preliminary validation in humans. In: *Circulation* 122 (2), S. 138–144. DOI: 10.1161/CIRCULATIONAHA.109.930636.

Galougahi, Keyvan Karimi; Liu, Chia-Chi; Bundgaard, Henning; Rasmussen, Helge H. (2012): β-Adrenergic regulation of the cardiac Na+-K+ ATPase mediated by oxidative signaling. In: *Trends in cardiovascular medicine* 22 (4), S. 83–87. DOI: 10.1016/j.tcm.2012.06.017.

Gauthier, C.; Leblais, V.; Kobzik, L.; Trochu, J. N.; Khandoudi, N.; Bril, A. et al. (1998): The negative inotropic effect of beta3-adrenoceptor stimulation is mediated by activation of a nitric oxide synthase pathway in human ventricle. In: *The Journal of clinical investigation* 102 (7), S. 1377–1384. DOI: 10.1172/JCI2191.

Gauthier, C.; Tavernier, G.; Charpentier, F.; Langin, D.; Le Marec, H. (1996): Functional beta3-adrenoceptor in the human heart. In: *The Journal of clinical investigation* 98 (2), S. 556–562. DOI: 10.1172/JCI118823.

Gauthier, Chantal; Rozec, Bertrand; Manoury, Boris; Balligand, Jean-Luc (2011): Beta-3 adrenoceptors as new therapeutic targets for cardiovascular pathologies. In: *Current heart failure reports* 8 (3), S. 184–192. DOI: 10.1007/s11897-011-0064-6.

Groenveld, Hessel F.; Tijssen, Jan G P; Crijns, Harry J G M; Van den Berg, Maarten P; Hillege, Hans L.; Alings, Marco et al. (2013): Rate control efficacy in permanent atrial fibrillation: successful and failed strict rate control against a background of lenient rate control: data from RACE II (Rate Control Efficacy in Permanent Atrial Fibrillation). In: *Journal of the American College of Cardiology* 61 (7), S. 741–748. DOI: 10.1016/j.jacc.2012.11.038.

Hany, Thomas F.; Gharehpapagh, Esmaiel; Kamel, Ehab M.; Buck, Alfred; Himms-Hagen, Jean; von Schulthess, Gustav K (2002): Brown adipose tissue: a factor to consider in symmetrical tracer uptake in the neck and upper chest region. In: *European journal of nuclear medicine and molecular imaging* 29 (10), S. 1393–1398. DOI: 10.1007/s00259-002-0902-6.

Hedetoft, Morten; Olsen, Niels Vidiendal (2014): Evaluation of endothelial function by peripheral arterial tonometry and relation with the nitric oxide pathway. In: *Nitric oxide : biology and chemistry / official journal of the Nitric Oxide Society* 42, S. 1–8. DOI: 10.1016/j.niox.2014.07.003.

Hochberg, Yosef (1988): A sharper Bonnferroni procedure for multiple tests of significance. In: *Biometrika* 75 (4), S. 800–802.

Hochberg, Yosef; Rom, Dror (1995): Extensions of multiple testing procedures based on Simes' test. In: *Journal of Statistical Planning and Inference* 48 (2), S. 141–152. DOI: 10.1016/0378-3758(95)00005-T.

Hofman, Michael S.; Hicks, Rodney J. (2011): White fat, factitious hyperglycemia, and the role of FDG PET to enhance understanding of adipocyte metabolism. In: *EJNMMI research* 1 (1), S. 2. DOI: 10.1186/2191-219X-1-2.

Holstila, Milja; Virtanen, Kirsi A.; Grönroos, Tove J.; Laine, Jukka; Lepomäki, Virva; Saunavaara, Jani et al. (2013): Measurement of brown adipose tissue mass using a novel dual-echo magnetic resonance imaging approach: a validation study. In: *Metabolism: clinical and experimental* 62 (8), S. 1189–1198. DOI: 10.1016/j.metabol.2013.03.002.

Hudsmith, Lucy E.; Petersen, Steffen E.; Francis, Jane M.; Robson, Matthew D.; Neubauer, Stefan (2005): Normal human left and right ventricular and left atrial dimensions using steady state free

precession magnetic resonance imaging. In: *Journal of cardiovascular magnetic resonance : official journal of the Society for Cardiovascular Magnetic Resonance* 7 (5), S. 775–782.

ICH Expert Working Group (27.10.1994): ICH HARMONISED TRIPARTITE GUIDELINE CLINICAL SAFETY DATA MANAGEMENT: DEFINITIONS AND STANDARDS FOR EXPEDITED REPORTING E2A. ICH Guideline E2A, vom step 4. Online verfügbar unter http://www.ich.org/fileadmin/Public_Web_Site/ICH_Products/Guidelines/Efficacy/E2A/Step4/E2A_Guideline.pdf, zuletzt geprüft am 18.09.2015.

Ikonomidis, Ignatios; Kadoglou, Nikolaos N P; Tritakis, Vlassis; Paraskevaidis, Ioannis; Dimas, Kleanthi; Trivilou, Paraskevi et al. (2014): Association of Lp-PLA2 with digital reactive hyperemia, coronary flow reserve, carotid atherosclerosis and arterial stiffness in coronary artery disease. In: *Atherosclerosis* 234 (1), S. 34–41. DOI: 10.1016/j.atherosclerosis.2014.02.004.

Ikonomidis, Ignatios; Ntai, Konstantina; Kadoglou, Nikolaos P E; Papadakis, Ioannis; Kornelakis, Michael; Tritakis, Vlassis et al. (2013): The evaluation of pulse wave velocity using Arteriograph and Complior apparatus across multiple cohorts of cardiovascular-related diseases. In: *International journal of cardiology* 168 (5), S. 4890–4892. DOI: 10.1016/j.ijcard.2013.07.025.

Kaneko, Yasuyuki; Floras, John S.; Usui, Kengo; Plante, Julie; Tkacova, Ruzena; Kubo, Toshihiko et al. (2003): Cardiovascular effects of continuous positive airway pressure in patients with heart failure and obstructive sleep apnea. In: *The New England journal of medicine* 348 (13), S. 1233–1241. DOI: 10.1056/NEJMoa022479.

Katz, A. M. (1985): Effects of digitalis on cell biochemistry: sodium pump inhibition. In: *Journal of the American College of Cardiology* 5 (5 Suppl A), S. 16–21.

Knöll, Ralph; Iaccarino, Guido; Tarone, Guido; Hilfiker-Kleiner, Denise; Bauersachs, Johann; Leite-Moreira, Adelino F. et al. (2011): Towards a re-definition of 'cardiac hypertrophy' through a rational characterization of left ventricular phenotypes: a position paper of the Working Group 'Myocardial Function' of the ESC. In: *European journal of heart failure* 13 (8), S. 811–819. DOI: 10.1093/eurjhf/hfr071.

Komajda, Michel; Lam, Carolyn S P (2014): Heart failure with preserved ejection fraction: a clinical dilemma. In: *European heart journal* 35 (16), S. 1022–1032. DOI: 10.1093/eurheartj/ehu067.

Kosmala, Wojciech; Przewlocka-Kosmala, Monika; Szczepanik-Osadnik, Hanna; Mysiak, Andrzej; O'Moore-Sullivan, Trisha; Marwick, Thomas H. (2011): A randomized study of the beneficial effects of aldosterone antagonism on LV function, structure, and fibrosis markers in metabolic syndrome. In: *JACC. Cardiovascular imaging* 4 (12), S. 1239–1249. DOI: 10.1016/j.jcmg.2011.08.014.

Lam, Carolyn S. P.; Carson, Peter E.; Anand, Inder S.; Rector, Thomas S.; Kuskowski, Michael; Komajda, Michel et al. (2012): Sex differences in clinical characteristics and outcomes in elderly patients with heart failure and preserved ejection fraction: the Irbesartan in Heart Failure with Preserved Ejection Fraction (I-PRESERVE) trial. In: *Circ Heart Fail* 5 (5), S. 571–578. DOI: 10.1161/CIRCHEARTFAILURE.112.970061.

Laurent, Stephane; Cockcroft, John; van Bortel, Luc; Boutouyrie, Pierre; Giannattasio, Cristina; Hayoz, Daniel et al. (2006): Expert consensus document on arterial stiffness: methodological issues and clinical applications. In: *European heart journal* 27 (21), S. 2588–2605. DOI: 10.1093/eurheartj/ehl254.

Lekakis, John; Abraham, Pierre; Balbarini, Alberto; Blann, Andrew; Boulanger, Chantal M.; Cockcroft, John et al. (2011): Methods for evaluating endothelial function: a position statement from the European Society of Cardiology Working Group on Peripheral Circulation. In: *European journal of cardiovascular prevention and rehabilitation : official journal of the European Society of Cardiology, Working Groups on Epidemiology & Prevention and Cardiac Rehabilitation and Exercise Physiology* 18 (6), S. 775–789. DOI: 10.1177/1741826711398179.

Lim, Tiong K.; Ashrafian, Houman; Dwivedi, Girish; Collinson, Paul O.; Senior, Roxy (2006): Increased left atrial volume index is an independent predictor of raised serum natriuretic peptide in patients with suspected heart failure but normal left ventricular ejection fraction: Implication for diagnosis of diastolic heart failure. In: *European journal of heart failure* 8 (1), S. 38–45. DOI: 10.1016/j.ejheart.2005.05.008.

Lloyd-Jones, Donald M. (2010): Cardiovascular risk prediction: basic concepts, current status, and future directions. In: *Circulation* 121 (15), S. 1768–1777. DOI: 10.1161/CIRCULATIONAHA.109.849166.

Lobysheva, Irina I.; Biller, Pauline; Gallez, Bernard; Beauloye, Christophe; Balligand, Jean-Luc (2013): Nitrosylated hemoglobin levels in human venous erythrocytes correlate with vascular endothelial function measured by digital reactive hyperemia. In: *PloS one* 8 (10), S. e76457. DOI: 10.1371/journal.pone.0076457.

Mahfoud, Felix; Urban, Daniel; Teller, Desiree; Linz, Dominik; Stawowy, Philipp; Hassel, Jan-Hendrik et al. (2014): Effect of renal denervation on left ventricular mass and function in patients with resistant hypertension: data from a multi-centre cardiovascular magnetic resonance imaging trial. In: *European heart journal* 35 (33), S. 2224. DOI: 10.1093/eurheartj/ehu093.

Mallincrodt, Craig H.; Lane, Peter W.; Schnell, Dan; Peng, Yahong; Mancuso, James P. (2008): Recommendations for the Primary Analysis of Continuous Endpoints in Longitudinal Clinical Trials. In: *Drug Information Journal* (42), S. 303–319, zuletzt geprüft am 06.08.2015.

Mancia, Giuseppe; Fagard, Robert; Narkiewicz, Krzysztof; Redon, Josep; Zanchetti, Alberto; Böhm, Michael et al. (2013): 2013 ESH/ESC guidelines for the management of arterial hypertension: the Task Force for the Management of Arterial Hypertension of the European Society of Hypertension (ESH) and of the European Society of Cardiology (ESC). In: *European heart journal* 34 (28), S. 2159–2219. DOI: 10.1093/eurheartj/eht151.

McMurray, John J V; Adamopoulos, Stamatis; Anker, Stefan D.; Auricchio, Angelo; Böhm, Michael; Dickstein, Kenneth et al. (2012): ESC guidelines for the diagnosis and treatment of acute and chronic heart failure 2012: The Task Force for the Diagnosis and Treatment of Acute and Chronic Heart Failure 2012 of the European Society of Cardiology. Developed in collaboration with the Heart Failure Association (HFA) of the ESC. In: *European journal of heart failure* 14 (8), S. 803–869. DOI: 10.1093/eurjhf/hfs105.

Moniotte, S.; Kobzik, L.; Feron, O.; Trochu, J. N.; Gauthier, C.; Balligand, J. L. (2001): Upregulation of beta(3)-adrenoceptors and altered contractile response to inotropic amines in human failing myocardium. In: *Circulation* 103 (12), S. 1649–1655.

Nagueh, Sherif F.; Appleton, Christopher P.; Gillebert, Thierry C.; Marino, Paolo N.; Oh, Jae K.; Smiseth, Otto A. et al. (2009): Recommendations for the evaluation of left ventricular diastolic function by echocardiography. In: *European journal of echocardiography : the journal of the Working Group on Echocardiography of the European Society of Cardiology* 10 (2), S. 165–193. DOI: 10.1093/ejechocard/jep007.

National Diabetes Education Initiative - NDEI: 2015 ADA Diabetes Guidelines - Summary Recommendations. Online verfügbar unter http://www.ndei.org/uploadedFiles/Common/NDEI/Treatment_Guidelines/ADA%202015%20Summary%20PDF.pdf, zuletzt geprüft am 15.01.2016.

NICE: Mirabegron for treating symptoms of overactive bladder | Guidance and guidelines | NICE. Online verfügbar unter http://www.nice.org.uk/guidance/ta290, zuletzt geprüft am 20.01.2016.

Ntusi, Ntobeko A B; Piechnik, Stefan K.; Francis, Jane M.; Ferreira, Vanessa M.; Rai, Aitzaz B S; Matthews, Paul M. et al. (2014): Subclinical myocardial inflammation and diffuse fibrosis are common in systemic sclerosis--a clinical study using myocardial T1-mapping and extracellular volume quantification. In: *Journal of cardiovascular magnetic resonance : official journal of the Society for Cardiovascular Magnetic Resonance* 16, S. 21. DOI: 10.1186/1532-429X-16-21.

Okin, Peter M.; Devereux, Richard B.; Jern, Sverker; Kjeldsen, Sverre E.; Julius, Stevo; Nieminen, Markku S. et al. (2004): Regression of electrocardiographic left ventricular hypertrophy during antihypertensive treatment and the prediction of major cardiovascular events. In: *JAMA* 292 (19), S. 2343–2349. DOI: 10.1001/jama.292.19.2343.

Ommen, S. R.; Nishimura, R. A.; Appleton, C. P.; Miller, F. A.; Oh, J. K.; Redfield, M. M.; Tajik, A. J. (2000): Clinical utility of Doppler echocardiography and tissue Doppler imaging in the estimation of left ventricular filling pressures: A comparative simultaneous Doppler-catheterization study. In: *Circulation* 102 (15), S. 1788–1794.

Orava, Janne; Nuutila, Pirjo; Lidell, Martin E.; Oikonen, Vesa; Noponen, Tommi; Viljanen, Tapio et al. (2011): Different metabolic responses of human brown adipose tissue to activation by cold and insulin. In: *Cell metabolism* 14 (2), S. 272–279. DOI: 10.1016/j.cmet.2011.06.012.

Pandey, Ambarish; Garg, Sushil; Matulevicius, Susan A.; Shah, Amil M.; Garg, Jalaj; Drazner, Mark H. et al. (2015): Effect of Mineralocorticoid Receptor Antagonists on Cardiac Structure and Function in Patients With Diastolic Dysfunction and Heart Failure With Preserved Ejection Fraction: A Meta-Analysis

and Systematic Review. In: *Journal of the American Heart Association* 4 (10), S. e002137. DOI: 10.1161/JAHA.115.002137.

Ponikowski, Piotr; Voors, Adriaan A.; Anker, Stefan D.; Bueno, Hector; Cleland, John G. F.; Coats, Andrew J. S. et al. (2016): 2016 ESC Guidelines for the diagnosis and treatment of acute and chronic heart failure: The Task Force for the diagnosis and treatment of acute and chronic heart failure of the European Society of Cardiology (ESC)Developed with the special contribution of the Heart Failure Association (HFA) of the ESC. In: *European heart journal* 37 (27), S. 2129–2200. DOI: 10.1093/eurheartj/ehw128.

Regitz-Zagrosek, Vera; Seeland, Ute (2011): Sex and gender differences in myocardial hypertrophy and heart failure. In: *Wien Med Wochenschr* 161 (5-6), S. 109–116. DOI: 10.1007/s10354-011-0892-8.

Rosa, Gian Marco; Ferrero, Simone; Nitti, Victor W.; Wagg, Adrian; Saleem, Tahir; Chapple, Christopher R. (2016): Cardiovascular Safety of β3-adrenoceptor Agonists for the Treatment of Patients with Overactive Bladder Syndrome. In: *European urology* 69 (2), S. 311–323. DOI: 10.1016/j.eururo.2015.09.007.

Ruth, Megan R.; Wellman, Tyler; Mercier, Gustavo; Szabo, Thomas; Apovian, Caroline M. (2013): An automated algorithm to identify and quantify brown adipose tissue in human 18F-FDG-PET/CT scans. In: *Obesity (Silver Spring, Md.)* 21 (8), S. 1554–1560. DOI: 10.1002/oby.20315.

Sarkar, Sanat K. (1998): Some Probability Inequalities for Ordered MTP 2 Random Variables: A Proof of the Simes Conjecture. In: *The Annals of Statistiscs* 26 (2), S. 494–504.

Solomon, Scott D.; Appelbaum, Evan; Manning, Warren J.; Verma, Anil; Berglund, Tommy; Lukashevich, Valentina et al. (2009): Effect of the direct Renin inhibitor aliskiren, the Angiotensin receptor blocker losartan, or both on left ventricular mass in patients with hypertension and left ventricular hypertrophy. In: *Circulation* 119 (4), S. 530–537. DOI: 10.1161/CIRCULATIONAHA.108.826214.

Tarone, Guido; Balligand, Jean-Luc; Bauersachs, Johann; Clerk, Angela; Windt, Leon de; Heymans, Stephane et al. (2014): Targeting myocardial remodelling to develop novel therapies for heart failure: a position paper from the Working Group on Myocardial Function of the European Society of Cardiology. In: *European journal of heart failure* 16 (5), S. 494–508. DOI: 10.1002/ejhf.62.

Turpen, Heidi Camus; Zimmern, Philippe E. (2015): Mirabegron: A new option in treating overactive bladder. In: *JAAPA : official journal of the American Academy of Physician Assistants* 28 (11), S. 16–18. DOI: 10.1097/01.JAA.0000472558.50124.7e.

van Marken Lichtenbelt Wouter D.; Vanhommerig Joost W.; Smulders Nanda M.; Drossaerts Jamie M.A.F.L.; Kemerink Gerrit J.; Bouvy Nicole D. et al.: Cold-Activated Brown Adipose Tissue in Healthy Men. Online verfügbar unter http://www.nejm.org/doi/pdf/10.1056/NEJMoa0808718, zuletzt geprüft am 08.09.2015.

van Marken Lichtenbelt, Wouter D; Vanhommerig, Joost W.; Smulders, Nanda M.; Drossaerts, Jamie M A F L; Kemerink, Gerrit J.; Bouvy, Nicole D. et al. (2009): Cold-activated brown adipose tissue in healthy men. In: *The New England journal of medicine* 360 (15), S. 1500–1508. DOI: 10.1056/NEJMoa0808718.

Verdecchia, P.; Reboldi, G.; Gattobigio, R.; Bentivoglio, M.; Borgioni, C.; Angeli, F. et al. (2003): Atrial Fibrillation in Hypertension. Predictors and Outcome. In: *Hypertension* 41 (2), S. 218–223. DOI: 10.1161/01.HYP.0000052830.02773.E4.

Verloop, Willemien L.; Beeftink, Martine M A; Nap, Alex; Bots, Michiel L.; Velthuis, Birgitta K.; Appelman, Yolande E. et al. (2013): Renal denervation in heart failure with normal left ventricular ejection fraction. Rationale and design of the DIASTOLE (DenervatIon of the renAl Sympathetic nerves in hearT failure with nOrmal Lv Ejection fraction) trial. In: *European journal of heart failure* 15 (12), S. 1429–1437. DOI: 10.1093/eurjhf/hft119.

Virtanen, Kirsi A.; Lidell, Martin E.; Orava, Janne; Heglind, Mikael; Westergren, Rickard; Niemi, Tarja et al. (2009): Functional brown adipose tissue in healthy adults. In: *The New England journal of medicine* 360 (15), S. 1518–1525. DOI: 10.1056/NEJMoa0808949.

Whalley, Gillian A.; Wright, Susan P.; Pearl, Ann; Gamble, Greg D.; Walsh, Helen J.; Richards, Mark; Doughty, Robert N. (2008): Prognostic role of echocardiography and brain natriuretic peptide in symptomatic breathless patients in the community. In: *European heart journal* 29 (4), S. 509–516. DOI: 10.1093/eurheartj/ehm647.

White, Steven K.; Sado, Daniel M.; Fontana, Marianna; Banypersad, Sanjay M.; Maestrini, Viviana; Flett, Andrew S. et al. (2013): T1 mapping for myocardial extracellular volume measurement by CMR: bolus only versus primed infusion technique. In: *JACC. Cardiovascular imaging* 6 (9), S. 955–962. DOI: 10.1016/j.jcmg.2013.01.011.

WMA: Ethical Principles for Medical Research Involving Human Subjects. Declaration of Helsinki, vom October 2013, zuletzt geprüft am 28.07.2015.

Yamamoto, Kazuhiro; Origasa, Hideki; Hori, Masatsugu (2013): Effects of carvedilol on heart failure with preserved ejection fraction: the Japanese Diastolic Heart Failure Study (J-DHF). In: *European journal of heart failure* 15 (1), S. 110–118. DOI: 10.1093/eurjhf/hfs141.

International Conference on Harmonisation of Technical Requirements for the Registration of Pharmaceutical Products for Human Use: ICH Harmonized Tripartite Guideline, "Clinical Data Safety Management: Definitions and Standards for Expedited Reporting". Recommended for Adoption at Step 4 of the ICH Process on 27 May 1994. Online verfügbar unter www.ifpma.org/ich5e.html#Safety.

Repertoire Commente des Medicaments. Online verfügbar unter http://www.cbip.be/GGR/index_intro.cfm?keyword=A6&anker=Tbl1b, zuletzt geprüft am 19.01.2016.

ICH-E6, vom 1996 (July 1996): Good Clinical Practice, Directive 75/318/EEC. Online verfügbar unter http://ec.europa.eu/health/files/eudralex/vol-10/3cc1aen_en.pdf, zuletzt geprüft am 17.09.2015.

(Geltung ab 26.10.2012): Gesetz über den Verkehr mit Arzneimitteln (Arzneimittelgesetz - AMG) zuletzt geändert durch das Gesetz zur Änderung arzneimittelrechtlicher und anderer Vorschriften vom 19.10.2012. In: BGBl. I S. 2192 (Nr. 50).

(Geltung ab 26.10.2012): Verordnung über die Anwendung der Guten Klinischen Praxis bei der Durchführung von klinischen Prüfungen mit Arzneimitteln zur Anwendung am Menschen (GCP-Verordnung - GCP-V) vom 19.10.2012. In: BGBl. I S. 2192 (Nr. 50).

17 PROTOCOL SIGNATURES

Confirmation of the Final Protocol

We hereby certify that this is the final version of the protocol:

Authorized representative of the sponsor and Coordinating investigator<br>Prof. Dr. Jean-Luc Balligand	22 october 2019<br>Date	[redacted]
Biometrician(s)<br>Dr. Oana Brosteanu	23.10.2019<br>Date	
Dr. Dirk Hasenclever	2019-10-23<br>Date	

18 PROTOCOL AGREEMENT

(to be signed by the investigator at each trial site before trial implementation/inclusion of patients at the trial site)

Herewith I declare that I have read and understood the present protocol and agree to honour each part of it. I will ensure that all the patients enrolled in the trial by my site will be treated, observed and documented in accordance with this protocol. I will ensure that all persons assisting with the study under my supervision are adequately informed about the protocol, the investigational product and their duties.

Date: ____________________________

Signature of Investigator: ____________________________

Affiliation/address (stamp): ____________________________

____

____

____

19 APPENDIX

19.1 Classification of Adverse Events

19.1.1 Degree of Severity

The degree of severity of an Adverse Event will be determined in accordance with the definitions in 8.1 and 8.4.

19.1.2 Assessment of Intensity

The assessment of the intensity accords with CTCAE V4.0

Mild Adverse Event	• asymptomatic or mild symptoms;<br>• clinical or diagnostic observations only;<br>• intervention not indicated.
Moderate Adverse Event	• minimal, local or noninvasive intervention indicated;<br>• limiting age-appropriate instrumental ADL*[1].
Severe Adverse Event	• medically significant but not immediately life-threatening;<br>• hospitalization or prolongation of hospitalization indicated;<br>• disabling;<br>• limiting self care ADL**
Life-threatening Adverse Event	• Life-threatening consequences;<br>• urgent intervention indicated
Death related to Adverse Event	

19.1.3 Determining the Causal Relationship

The investigator/ the deputy or the authorized medical staff must assess whether or not the Adverse Event is causally related to the administration of the trial medication. The following classification is to be used.

- Reasonable possibility
- No reasonable possibility

A reasonable possibility exists, if one of the following WHO-UMC criteria is met:

- occurring in a plausible time relationship to drug administration, and which cannot be explained by concurrent disease or other drugs or chemicals. The response to withdrawal of the drug (dechallenge) should be clinically plausible. The event must be

[1] **Activities of Daily Living (ADL):**
*Instrumental ADL refer to preparing meals, shopping for groceries or clothes, using the telephone, managing money, etc.
**Self care ADL refer to bathing, dressing and undressing, feeding self, using the toilet, taking medications, and not bedridden.

definitive pharmacologically or phenomenologically, using a satisfactory rechallenge procedure if necessary.

- with a reasonable time sequence to administration of the drug, unlikely to be attributed to concurrent disease or other drugs or chemicals, and which follows a clinically reasonable response on withdrawal (dechallenge). Rechallenge information is not required to fulfil this definition.
- with a reasonable time sequence to administration of the drug, but which could also be explained by concurrent disease or other drugs or chemicals. Information on drug withdrawal may be lacking or unclear.
- more data is essential for a proper assessment or the additional data are under examination
- cannot be judged because information is insufficient or contradictory, and which cannot be supplemented or verified

No reasonable possibility exists, if the following WHO-UMC criterion is met:

- with a temporal relationship to drug administration which makes a causal relationship improbable, and in which other drugs, chemicals or underlying disease provide plausible explanations.

19.1.4 Expected/Unexpected

Adverse Events are unexpected if they do not occur in the manner or with the intensity described in the *SmPC/Investigator's Brochure* (see investigator's files).

19.1.5 Outcome of an Adverse Event

The outcome of an Adverse Event is classified as follows:

- recovered/resolved
- recovering/resolving
- not recovered/not resolved
- recovered/resolved with sequelae
- fatal*
- unknown

*Note: A patient's death is not in itself an event, but the consequence of one. The event that led to the patient's death must be documented completely and reported even if death occurs four weeks after stopping medication and independent of whether or not there is a relation to the therapy or not.

19.2 Definitions

19.2.1 NYHA-Class

NYHA Class	Symptoms
I	Cardiac disease, but no symptoms and no limitation in ordinary physical activity, e.g. no shortness of breath when walking, climbing stairs etc.
II	Mild symptoms (mild shortness of breath and/or angina) and slight limitation during ordinary activity.

III	Marked limitation in activity due to symptoms, even during less-than-ordinary activity, e.g. walking short distances (20–100 m).<br>Comfortable only at rest.
IV	Severe limitations. Experiences symptoms even while *at rest*. Mostly bedbound patients.

19.3 Acronyms

ABPM	Ambulatory blood pressure monitoring
AE	Adverse Event
AF	Atrial Fibrillation
AR	Adrenergic Receptor
BMI	Body Mass Index
BPM	Beats per Minute
CA	Competent Authority
CTC	Clinical Trial Centre
DSMB	Data Safety and Monitoring Board
EAC	Event Adjudication Committee
EF	Ejection Fraction
EC	Ethics Committee
GCP	Good Clinical Practice
HCT	Hematocrit
HFpEF	Heart failure with preserved ejection fraction
HFrEF	Heart failure with reduced ejection fraction
HR	Heart Rate
ICH	International Conference on Harmonisation
ICMJE	International Committee of Medical Journal Editors
ISF	Investigator Site File
LGE	Left Gadolinium Enhancement
LV	Left Ventricle
MHRD	Maximum recommended human dose
OAB	Overactive Bladder
PKA	Protein Kinase A
PTCA	Percutaneous transluminal coronary angioplasty
SAE	Serious Adverse Event
SAR	Serious Adverse Reaction
SUSAR	Suspected Unexpected Serious Adverse Reaction
TMF	Trial Master File
UCL	Université Catholique de Louvain

WHO-UMC	World Health Organization – Uppsala Monitoring Centre
WOCB	Women of child bearing potential

19.4 Labelling

The master label(s) of trial medication will be supplied as separate document(s).

Statistical Analysis Plan
(SAP)

for the trial

A multi-centre randomized, placebo-controlled trial of mirabegron, a new beta3-adrenergic receptor agonist on the progression of left ventricular mass and diastolic function in patients with structural heart disease

(BETA3_LVH)

NCT01067703

Coordinating investigator
Prof. Jean-Luc Balligand
Full professor, Head of Department
Pharmacology and Therapeutics (FATH)
Avenue Mounier, 52, B1.53.09
1200 Brussels, Belgium
Phone: +32-2-7645260/+32-2-7645262
Fax: +32-2-7645269

Biometry
Dr. Oana Brosteanu, Dr. Dirk Hasenclever
Clinical Trial Centre Leipzig
Universität Leipzig
Härtelstraße 16-18,
04107 Leipzig, Germany
Phone: +49-341-97 16 250
Fax: +49-341-97 16 189

Sponsor
UNIVERSITÉ CATHOLIQUE DE LOUVAIN
PLACE DE L'UNIVERSITE 1, 1348 LOUVAIN LA NEUVE, Belgium

Date of the document: 2022-08-09
Version of the document: Final 1.0

We hereby approve the Statistical Analysis Plan in its final version:

Coordinating Investigator	10-08-2022	J-L Balligand
	Date	Signature
Biometrician		09.08.2022<br>X<br>Dirk Hasenclever<br>Signiert von: Dr. Dirk Rainer Hasenclever
	Date	Signature
Biometrician		09.08.2022<br>X<br>Oana Brosteanu<br>Signiert von: Dr. Oana Brosteanu
	Date	Signature

Table of contents

1 Introduction 5

2 Changes in the planned analysis 5

2.1 Fine-tuning the basic analytic model 5
2.2 Sample size revisited in the light of the COVID-19 pandemic 5
2.3 Additional secondary endpoints 6

3 Endpoints and further variables 6

3.1 Primary Endpoints 6
3.1.1 Change in left ventricular mass index (LVMI) 6
3.1.2 Change in diastolic function (E/e’) 7
3.1.3 Dealing with co-primary endpoints 7
3.2 Secondary Endpoints 7
3.3 Safety endpoints 9
3.3.1 Adverse and serious adverse events 9
3.3.2 Safety events of special interest 9
3.4 Covariates for confirmatory analyses 10
3.5 Further Variables 10
3.5.1 Baseline characteristics 10
3.5.2 Trial intervention 12

4 General Analysis Definitions 12

4.1 Study periods 12
4.2 Analysis Populations 12
4.3 Handling of centre effects 13

5 Planned analysis 13

5.1 Demographic and other baseline parameter 13
5.2 Concomitant Diseases and Medication 13
5.3 Compliance with regard to the Study Intervention 13
5.4 Primary and secondary efficacy endpoints 14
5.4.1 Choice of scale of analysis 14
5.4.2 Basic analytic model 14
5.4.3 Sensitivity analyses 16
5.5 Subgroup analysis 16
5.6 Safety aspects 17
5.6.1 Adverse events 17
5.6.2 Serious adverse events 18
5.6.3 Events of special interest 18

6 References 19

7 Appendix 19

7.1 Abbreviations 19

1 Introduction

The purpose of this document is to provide a detailed elaboration of the statistical analysis described in the protocol, including detailed procedures for the confirmatory analysis of the primary and secondary endpoints and other variables.

The Statistical Analysis Plan (SAP) assumes familiarity with the Study Protocol (Version Final 2.0 dated 13.11.2015), including Protocol Amendments.

The SAP is based on the planned analysis specification as written in the study protocol Section 9 “Biometry”. SAP readers may consult the study protocol for more background information on the study, e.g., on study objectives, study design and population, trial intervention, definition of measurements and variables, planning of sample size, and randomization.

R 4.2.0 (R packages named below) will be used for all statistical analyses.

2 Changes in the planned analysis

2.1 Fine-tuning the basic analytic model

There were two challenges in choosing the structure of the basic analytic model:

- Dealing with missing of failed measurements at study visits and
- Adjusting for chance baseline imbalances

Because failed measurements were already expected to occur at baseline, in the study protocol we chose a linear mixed model with a 3-dimentional random effect (Baseline, V3, V5) with a general variance covariance structure. The random effect models the intra-individual dependence in the vector of residuals from the mean structure for each patient. This random effect includes the baseline measurement. This allows the model to accommodate all patients with at least one valid measurement.

However, as the baseline measurement is included in the random effect and can be missing, it cannot be used as a fixed effect adjustment covariate, without restricting the analysis set to patients with valid baseline only.

Fitzmaurice et al. 2004 (Chapter 5.7) propose a solution to this short-coming: Because the study is randomised, it is justified to force the model to estimate a common mean at baseline without separating the arms. This change in the mean structure of the model performs an indirect baseline adjustment via its influence on the random effect. Simulations of Fitzmaurice et al. 2004 (and as well as our own) confirm that results with this indirect baseline adjustment are nearly identical to using baseline as fixed effect covariate. In addition, the gain in power compared to unadjusted analysis is the same.

Therefore, indirect baseline adjustment was added to the basic analysis (details see 5.4 below).

2.2 Sample size revisited in the light of the COVID-19 pandemic

The COVID-19 pandemic affected the study in slowing down accrual and increasing the risk for dropouts due to missed visits. Therefore, the steering committee of BETA3_LVH discussed on 2021-01-19 whether to end the study as planned or to increase the sample size due to a higher than expected dropout rate.

In a statistical appraisal (NoReasonsForOverrecuiting 2021-03-01.pdf), the eventual dropout rates were projected and the assumptions underlying the sample size calculations revisited based on blinded data.

- With conservative assumptions, the projected number of fully + partially informative patients at the time was at least 75% for both primary endpoints.

- The original sample size calculation was based on a very conservative assumption on the standard deviation of the changes in E/e'. The study data at the time suggested sd < 2.5 instead of sd = 3.
- Thus, BETA3_LVH is sufficiently powered for drop-out rates up to 30%, although initially a dropout rate of only 8% was assumed.

Accrual into the study was stopped as planned when the target sample size was achieved (decision of the Steering Committee 2021-04-06).

2.3 Additional secondary endpoints

While we were completing our data collection, the group of H. Bundgaard in Denmark published the results of an independent study on mirabegron in patients with severe heart failure with reduced ejection fraction (<35%); while mirabegron was used at 300 mg daily (under full beta1-blocker therapy) for only 1 week, invasive hemodynamic measurements showed a significantly larger increase in cardiac index in the verum compared to placebo (Bundgaard H et al. 2022). Therefore, we decided to add a secondary key endpoint reflecting the LV systolic function in our analysis, i.e. left ventricular ejection fraction (LVEF) measured by high precision cMRI.

Likewise, we are aware of the protocol of the SPHERE study (Garcia-Lunar I. et al 2020), testing the effect of mirabegron (vs. placebo) in patients with both pre- and post-capillary pulmonary hypertension from both heart failure with preserved or reduced ejection fraction, where right ventricle ejection fraction, reflecting the RV function, is a key secondary endpoint (and pulmonary vascular resistance is the primary endpoint). As we postulate a favourable myocardial remodelling and improved diastolic function with mirabegron as our main hypothesis, if this is verified, we expect an improvement in filling pressures that may have an impact on pulmonary vascular pressures and RV function. For this reason, we decided to add an index of RV systolic function as a secondary key endpoint, i.e. right ventricular ejection fraction (RVEF) measured by high-precision cMRI.

3 Endpoints and further variables

3.1 Primary Endpoints

Two equally ranked primary endpoints are defined, in order to assess both structural and functional aspects of left ventricular remodelling.

The measurements at baseline, 6 months and 12 months will be analysed together by a linear mixed model for repeated measurements (see 5.4). The treatment effect at 12 months is the primary contrast of interest. The treatment effect at 6 months is a secondary endpoint.

3.1.1 Change in left ventricular mass index (LVMI)

Change in left ventricular mass index (**LVMI** in g/m^2 is defined as left ventricular mass (LVM) divided by body surface area, BSA) measured at baseline, 6 months (V3 = visit 3), and 12 months (V5 = visit 5) after randomisation. Cardiac MRI is performed locally according to a standardized protocol, and LVM is measured in the central MRI core lab.

LVMI will be computed using

- o LVM as measured by the core lab
- o body surface area (BSA) derived from height and weight as documented in the electronic CRF for the respective visit, using the DuBois formula

$$BSA\ [m^2] = 0.007184\ m^2 * height\ [cm]^{0.725} * weight\ [kg]^{0.425}$$

The MRI core lab has classified the quality of the LVM measurement with a quality score from 0 to 3, meaning

- o 0 = OK
- o 1 = minor problems (values probably ok)

- o 2 = major problems (affecting values)
- o 3 = data missing

MRI scans classified as having "major problems (affecting values)" will be counted as missing.

3.1.2 Change in diastolic function (E/e')

Change in diastolic function, assessed as the ratio of peak early transmitral ventricular filling velocity to early diastolic tissue Doppler velocity (**E/e'**) measured at baseline, 6 and 12 months after randomisation. This parameter is assessed by echocardiography, performed locally according to a standardized protocol, and is measured in the central echocardiography core lab.

E/e' will be measured by the echo core lab according to the echo core lab echo image analysis plan. Only average E/e' values will be used for the statistical analysis of this co-primary endpoint. E/e' average values are calculated as the ratio of E divided by the average of e' septal and e' lateral. In cases either e' septal or e' lateral cannot be reliably measured by the echo core lab, the respective E/e' average value will be set as missing value due to low image quality in the echo core lab report.

3.1.3 Dealing with co-primary endpoints

This trial wants to demonstrate that mirabegron as add-on to standard treatment compared to standard treatment alone improves at least one of the two primary endpoints over 12 months.

The null hypotheses are that the 12 month mean changes from baseline mean are identical in both arms. Two-sided tests are used, i.e. the alternative is that mean changes from baseline mean differ by arm.

The Hochberg method will be used to adjust for endpoint multiplicity: If both p-values are below 0.05 we will claim efficacy in both primary endpoints; if otherwise the smallest p-value is below 0.025 we will claim efficacy in the respective primary endpoint. This procedure controls the family-wise error rate (FWER) in the strong sense at a two-sided significance level of 5%. (Hochberg 1988). The Hochberg procedure seems appropriate since we expect non-negatively correlated test-statistics.

3.2 Secondary Endpoints

- Further cMRI endpoints (all measured in the central MRI core lab)
    - o Cardiac fibrosis at baseline and at 12 months (V5). Fibrosis is a key pathogenic mechanism of diastolic dysfunction, which is at the origin of HFpEF. Cardiac fibrosis has been shown to be reduced by beta3AR in pre-clinical studies.
      The following separate markers of cardiac fibrosis are analyzed:

      <u>Late Gadolinium Enhancement</u>
        - ▪ LGEvf: Late Gadolinium Enhancement volume fraction

      <u>Native T1</u>
        - ▪ T1_native: T1 relaxation time

      <u>Extracellular Volume Fraction</u>
        - ▪ ECV_AllDataPoints as summary (based on ECV_5m, ECV_15m, ECV_30m as raw data)
        - ▪ The four parameters are non-independent

      The MRI core lab has classified the quality of measurements of cardiac fibrosis with a quality score from 0 to 3. As suggested by the core lab, only measurements classified as 0 or 1 will be used for analysis. All other measurements will be counted as missing.

    - o Left atrial volume index (LAVOLI) at baseline and at 12 months (V5). This parameter determines diastolic filling (and was shown to predict treatment efficacy in HFpEF in the J-

DHF trial (Yamamoto et al. 2013).

LAVOLI will be computed using

- Left atrial volume (LAVOL) as measured by the core lab
- body surface area (BSA) derived from height and weight as documented in the electronic CRF for the respective visit, using the DuBois formula

$$BSA\ [m^2] = 0.007184\ m^2 * height\ [cm]^{0.725} * weight\ [kg]^{0.425}$$

The MRI core lab has classified the quality of the LAVOL measurement with a quality score from 0 to 3. As suggested by the core lab, only measurements classified as 0 or 1 will be used for analysis. All other measurements will be counted as missing.

- Left Ventricular Ejection fraction (LVEF) by cardiac MRI at Baseline and 12 months

  The MRI core lab has classified the quality of the left ventricular measurements with a quality score from 0 to 3. As suggested by the core lab, only measurements classified as 0 or 1 will be used for analysis. All other measurements will be counted as missing.

- Right Ventricular Ejection Fraction (RVEF) by cardiac MRI at Baseline and 12 months

  The MRI core lab has classified the quality of the right ventricular measurements with a quality score from 0 to 3. As suggested by the core lab, only measurements classified as 0 or 1 will be used for analysis. All other measurements will be counted as missing.

- LV mass index (by cardiac MRI measured by the MRI core lab) at 6 months,

- Diastolic function (E/e'), measured by the echo core lab, at 6 months;

- Laboratory parameters at baseline and at 3 (V2), 6 (V3) and 12 months (V5), measured by the central core lab
  - serum biomarkers
    - NT-proBNP
    - hsTnT
    - Galectin3
    - GDF15
  - metabolic parameters
    - Glycemic profile
    - HbA1c
    - Total cholesterol
    - Calculated LDL cholesterol
    - HDL cholesterol
    - Triglycerides
    - modified HOMA test (HOMA test /%S, HOMA test / % B)
    - Insulin
- Maximal exercise capacity (peak VO_2) at baseline and 12 months (V5).

  Up to four different parameters are provided depending on the device used and are recorded in the eCRF:
  - VO_2max in [L/min]
  - VO_2max in [mL/min/kg]
  - VO_2max in [L/min] in % predicted values
  - VO_2max in [mL/min/kg] in % predicted values

In principle, all parameters should be interconvertible:

- There is a formula provided by Wonisch et al for the predicted values which could be used for transformation of absolute values into % predicted values
- Weight is recorded in the eCRF, allowing for transformation between the two units used for absolute measurements.

The preferred secondary endpoint for analysis is peak VO_2max in [mL/min/kg] according to the core lab.

In rare cases where the preferred secondary endpoint for analysis is VO_2max in [mL/min/kg] is not documented directly, but any of the three other endpoints, this formula will be used to derive it.

Because of the large number of secondary endpoints and in order to limit multiplicity problems, we prioritize those secondary efficacy endpoints in which we particularly expect a potential treatment effect:

Key secondary endpoints are:

- NT-proBNP (as continuous variable)
- Left atrial volume index
- myocardial fibrosis: ECV_AllDataPoints
- metabolic measurement: HOMA test: HOMA /%S
- peak VO_2 max (ml/min/kg)
- ~~LVEF~~
- RVEF

All other secondary endpoints are of lower ranking interest.

3.3 Safety endpoints

3.3.1 Adverse and serious adverse events

Adverse and serious adverse events are recorded on corresponding forms, which contain the following information:

- Type of event
- Start and end date
- Maximal intensity (mild / moderate / severe / life-threatening / death related to AE)
- Therapy of event (yes / no)
- Outcome of event (recovered / recovering / not recovered / recovered with sequelae / fatal / unknown)
- Causal relationship with trial drug (reasonable possibility / no reasonable possibility)
- Seriousness (yes / no)
- Seriousness criterion (death / life risk / hospitalization / disability / birth defect / Intervention required)
- Action taken with trial drug due to event (drug withdrawn / dose reduced / dose increased / dose not changed / unknown / not applicable)

Adverse Events (AE) and Serious Adverse Events (SAE) will be coded according to Medical Dictionary for Regulatory Activities (MedDRA) Version 24.1. For analysis, classifications by body system and by preferred term will be used.

3.3.2 Safety events of special interest

In addition to the adverse event documentation, the following safety events of special interest were closely monitored.

Blood pressure

Three measurements of blood pressure (systolic and diastolic), taken after 5 min of rest in sitting position are documented at every visit. The average of these three measurements is used for further analyses.

Elevated blood pressure (yes / no) is defined as systolic blood pressure > 160 mm Hg or diastolic blood pressure > 100 mm Hg after start of study treatment.

Highly elevated blood pressure (yes / no) is defined as systolic blood pressure > 180 mm Hg or diastolic blood pressure > 110 mm Hg after start of study treatment.

Hepatic impairment

Alanine aminotransferase (ALT) and aspartate aminotransaminase (AST) are measured locally at baseline and at visits V1, V2, V3 and V5.

Hepatic impairment (yes / no) is defined as ALT > 2 x upper level normal or AST > 2 x upper level normal after start of study treatment, where the upper level of normal is defined by the local laboratory.

Renal impairment

Serum Creatinine (SCr) is measured locally at baseline and at visits V1, V2, V3 and V5. The estimated glomerular filtration rate eGFR is derived from this measurement using the MDRD formula:

$$eGFR = 175 \times (SCr\ in\ mg/dl)^{-1.154} \times (age\ in\ years)^{-0.203} \times 0.742\ [if\ female] \times 1.212\ [if\ Black]$$

Renal impairment (yes / no) is defined as eGFR < 30 ml/min after start of study treatment.

Anaemia

Haemoglobin (Hb) is measured locally at baseline, V3 and V5.

Anaemia (yes / no) is defined as Hb < 13 g/dl in males and Hb < 12 g/dl in females after start of study treatment.

Atrial fibrillation /atrial flutter

Any event of atrial fibrillation (yes / no) or atrial flutter (yes / no) after start of study treatment.

Mortality

Death of any cause (yes / no) after start of study treatment.

3.4 Covariates for confirmatory analyses

According to the protocol, atrial fibrillation (yes / no) and diabetes mellitus (yes / no) as documented at randomisation will be incorporated as covariates in the primary analysis because they were used to stratify randomisation.

Unfortunately, the documentation at time of randomisation did not distinguish between permanent or paroxysmal atrial fibrillation.

3.5 Further Variables

3.5.1 Baseline characteristics

Age [years] is computed as difference between year of registration and birth year as recorded on the patient registration CRF.

Sex is recorded on the patient registration CRF.

Ethnic origin (Caucasian / Asian / African / other) is recorded on the patient registration CRF.

Baseline Assessments

The following baseline assessments are recorded on the baseline CRF:

- **Body mass index** [kg/m^2] is derived from weight [kg] and height [m] as recorded on the baseline CRF
- **Waist / hip ratio** is derived from waist circumference [cm] and hip circumference [cm] as recorded on the baseline CRF
- **Blood pressure** [mm Hg]: as defined in 3.3.2
- **Heart frequency** [bpm] after 5 min rest
- **Left ventricular mass index** [g/m^2] as determined by local echocardiography and recorded at registration.
- **End diastolic wall thickness** [mm] as determined by local echocardiography and recorded at registration.
- **Ejection fraction** [%] as determined by local echocardiography and recorded at registration.
- **QT-interval [ms]** as determined at screening ECG and recorded at registration**.**
- **Atrial fibrillation (yes / no)** is recorded thrice:
    - at patient registration
    - on the baseline CRF (result of the baseline electrocardiogram: rhythm coded as sinus rhythm / atrial fibrillation / atrial flutter)
    - as assessed by the central echocardiography core lab from the baseline echo

  For atrial fibrillation in general (without distinction between permanent and paroxysmal), the status reported on the registration CRF will be used. However, this item is crosschecked with the ECG resp. the echo documentation triggering queries in case of inconsistency.

  It may be possible to derive the distinction permanent versus paroxysmal atrial fibrillation based on additional information from ECG resp. baseline echocardiography.

<u>**Heart Failure classification**</u>

- **NYHA class** (no heart failure / I / II / III / IV)
- If NYHA class I-IV: **classification of heart failure** (right sided / left-sided / global / unknown)

<u>**Cardiovascular risk factors**</u>

The following risk factors are recorded on the baseline CRF. Documentation as “unknown” is counted as missing.

- **Diabetes mellitus** (yes / no) and type of diabetes mellitus

  Diabetes mellitus (yes / no) is recorded twice: at patient registration and on the baseline CRF.

  The status reported on the baseline CRF will be used

  We crosschecked with the documentation on the registration form as well as the reported use of anti-diabetes drugs triggering queries in case of inconsistency.
- **Hyperlipidaemia** (yes/no/unknown)
- **Hyperuricemia** (yes/no/unknown)
- **Hypertension** (yes/no/unknown)
- **Sleep apnoea syndrome** (yes/no/unknown)
- **Myocardial infarction in relatives of first degree** (parents, siblings, children) before 60 years of age (yes/no/unknown)
- **Smoking habits** (never / yes / former smoker) as packyears (=twenty cigarettes smoked every day for one year) in active or former smoker
- **Drinks per week:** 1 drink = 0.25 l beer or 0.1 l wine or 0.02 l spirits

Current medication

On the baseline CRF, a list of relevant current medication is specified and current application recorded as (yes / no)

Concomitant diseases

On the baseline CRF, a list of relevant concomitant disease is specified and current diseases are recorded as (yes / no /unknown). Documentation as "unknown" is counted as missing.

Concomitant diseases documented in addition to the pre-specified list will be coded according MedDRA preferred terms.

3.5.2 Trial intervention

Compliance with the randomised intervention (mirabegron/placebo) will be assessed by:

Total dose taken

- **Days on treatment**, i.e. total number of capsules handed out to the patient and not documented as returned

Days on treatment will be computed using the following documentation on the CRF:

- Number of complete boxes (à 4 blisters = 28 capsules in total) handed out
- Number of capsules returned

Intended total dose

Days on study, i.e. from randomisation to individual visit 5 after 12 months or to the day of premature study termination (if before visit 5)

Relative dose

Days on treatment divided by days on study

4 General Analysis Definitions

4.1 Study periods

The following study periods are distinguished for analysis

- Screening period
- Baseline Assessment
- Treatment period, including regular assessment of end points
- Follow-up at 1 month after end of treatment

4.2 Analysis Populations

Analysis populations are defined according to the protocol of the BETA3_LVH-Study (see protocol chapter 9.5.1).

The **Full Analysis Set** (FAS, also called intention-to-treat (ITT) population) includes all randomised patients with valid informed consent and at least one valid measurement in at least one of the primary endpoints (baseline, 6 months (V3) or 12 months (V5)).

The confirmatory analysis is performed on the FAS and is based on the randomized arm.

The **Per-Protocol Set (PPS)** is a subset of the FAS consisting of **patients without major protocol deviations.**

The PPS approximates an ideal (contra-factual) study in which conduct was perfect and patients compliant and selected such that they tolerate the treatment. In a study to demonstrate a treatment effect, the PPS analysis is particularly useful in case of a non-significant result, in order to assess whether the ideal biological efficacy was compromised by protocol deviations.

As specified in the protocol, the following **protocol deviations are classified as major**:

- Violation of an eligibility criterion
- Patients who received less than 50% of the total dose taken of study medication (mirabegron resp. placebo)
- Patients who received both mirabegron and placebo due to a mix-up of the study drug boxes
- No valid measurement of any of the primary endpoints at the 12 month (V5) visit.

However, patients remain in the PPS if:

- Study medication had to be interrupted because of medical reasons, e.g. (S)AE, and therefore the patient received lower than 50% of the intended cumulative dose,
- they deceased during the treatment phase

The **Safety Analysis Set** consists of all randomised patients belonging to the FAS who received at least one dose of study medication. Patients will be analysed according to treatment taken.

4.3 Handling of centre effects

Random centre effects are not included in the primary analytical model since the number of patients per trial site is small in most centres in order to avoid convergence problems in over-complex models. A subgroup analysis of the treatment effect by centre will be provided as sensitivity analysis.

5 Planned analysis

A flowchart according to the CONSORT statement will describe the disposition of all patients registered to the trial detailing screening failure before randomization, withdrawals, drop-outs and inclusion in the analyses sets defined above. Respective listings will be provided.

In addition, patients with major protocol violations excluded from the PPS will be listed.

Standard methods of descriptive statistics will be used always indicating the number of valid and missing values. Summary statistics will be reasonably rounded to avoid pseudo-precision.

5.1 Demographic and other baseline parameter

Demographic and other baseline parameter will be described by randomization arm.

5.2 Concomitant Diseases and Medication

Frequencies of concomitant diseases and medication will be described by randomization arm.

5.3 Compliance with regard to the Study Intervention

Patients will be listed on whom no intervention or an intervention not corresponding to the randomization arm was performed.

Percentage of received dose of intended dose will be described by randomization arm and plotted as an empirical cumulative distribution function.

5.4 Primary and secondary efficacy endpoints

Confirmatory analysis follows the intention to treat principle as close as possible and will be based on the full analysis set.

5.4.1 Choice of scale of analysis

All time courses of metric measurements will be analysed on an appropriate scale such that linear models are adequate.

- The two primary endpoints will be analysed without scale transformation.
- The laboratory parameters
    - GDF15
    - NT-proBNP
    - hs TnT
    - Glycemic Profile
    - Insulin
    - HOMA test / % B
    - HOMA test / % S
    - Triglycerides

  are analysed on the log10 scale.
- All other parameters are analysed without scale transformation.

5.4.2 Basic analytic model

Analyses of both primary endpoints are identically structured. For secondary endpoints the same model is used, possibly adapting the dimension of the random effect to the number of visits.

We use a linear mixed model for repeated measurements. This choice of a repeated measurements linear mixed model as the primary analytic model is in line with the recommendations of (Mallincrodt et al. 2008). There is a certain rate of patients with missing measurements on the endpoints, also at baseline.

A 3-dimensional random effect with a general unstructured variance covariance matrix is used to model the dependence of measurements within-patients.

The mean structure is modelled using (0/1)-coded variables and include the following fixed effects:

- Intercept corresponding to baseline
- Timepoints: V3 and V5
- Treatment: Verum ,
- Treatment by Timepoint interaction at 6 months, 12 months: V3:Verum, V5:Verum
- Atrial fibrillation,
- Diabetes mellitus

The model implicitly uses the estimated covariance matrix to deal with missed visits. We expect that missing endpoints will be missing at random (MAR) given the specified model structure above. The above model can deal with patients with incomplete data in the endpoints as long as at least one valid measurement is documented.

The Treatment by baseline interaction is not included in the model as patients are randomised at baseline. This indirectly adjusts for possible random chance fluctuation at baseline and generally increases the power (Fitzmaurice et al. 2004, Chapter 5.7). For further discussion compare 2.1 above.

The model is fitted using the “nlme” R-package using the formula:

```
nlme::lme(Endpoint ~  V3 + V5 + V3:Verum + V5:Verum  +  AF +  DB ,
          random = ~ BASE + V3 + V5 - 1 | PATNO,
          data = Beta3, method="REML")
```

As specified in the protocol, analysis will be based on restricted maximum likelihood (REML), which is a method to remove bias from the estimation of variance components and was shown to be advantageous in simulation studies (e.g. McNeish D 2017).

The contrast of interest for the primary endpoints is the treatment by visit interaction V5:Verum at 12 months. Respective inference will be based on Wald type confidence intervals and p-values.

The secondary endpoints concerning treatment effects at 6 months will be assessed **within the same model** by looking at the treatment by visit interaction at 6 months.

The model results will be illustrated as in this prototype figure using fake example data for e/e’ depicting

- the 95% confidence intervals for the estimates of the mean time courses by treatment arm as well as
- p-values for the null hypotheses of no treatment effect at the respective time points and

The intervention effect estimate will be quantified the 95% confidence interval for the main treatment effect at 12 months.

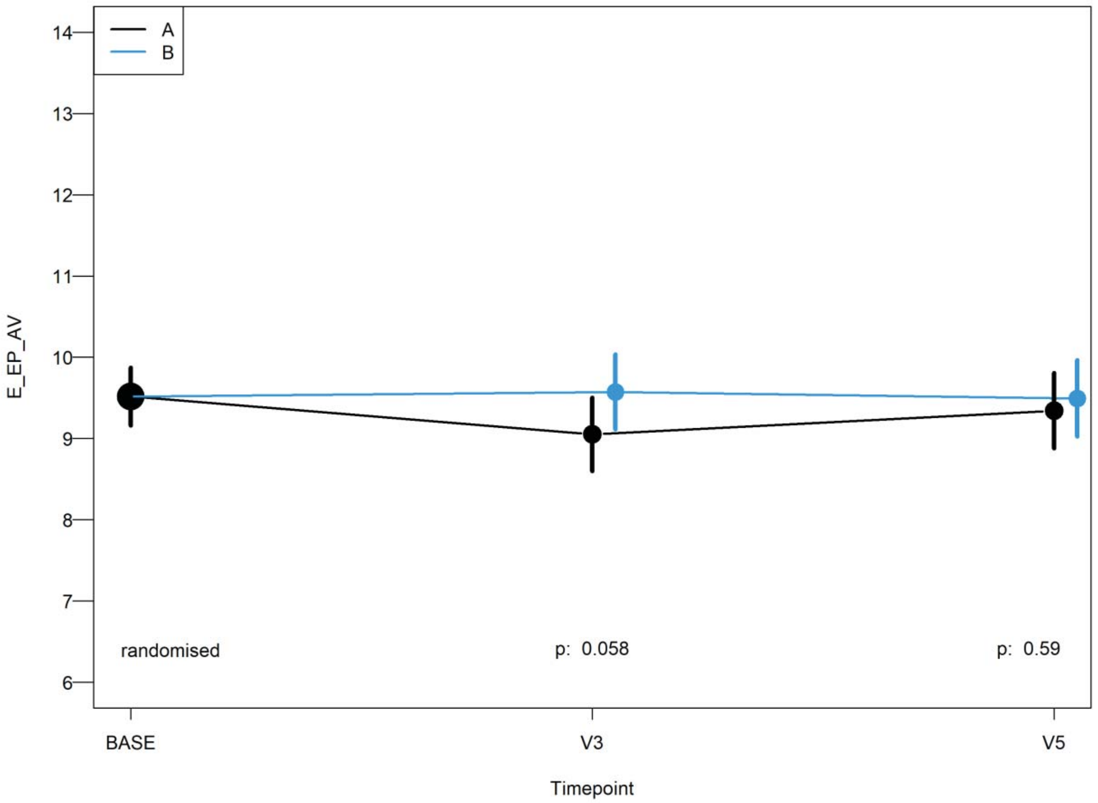

5.4.3 Sensitivity analyses

There will be two sensitivity analyses for both primary endpoints:

1. Analysis restricted to the per protocol population. The per-protocol analysis tries to answer the study question in a hypothetical world in which the treatment strategy was implemented optimally. The per-protocol analysis becomes important, when superiority is not shown: Is there simply no effect or did we miss to demonstrate the treatment effect due to protocol deviations?
2. An ANCOVA with Baseline values, Atrial fibrillation, Diabetes mellitus and randomised arm as covariates in all randomized patients with baseline and 12 months measurements. This is a complete case analysis using a robust simple linear model without random effects.

The protocol also mentions an ANCOVA “with imputation of missing values by last information carried forward (LOCF)”. We drop this, since last information carried forward (LOCF) is an obsolete, no more state-of-the-art method.

5.5 Subgroup analysis

No confirmatory subgroup analyses are planned.

Strictly exploratory subgroup analyses for the two primary endpoints will be performed to assess whether the **overall treatment effect is homogenous across relevant subgroups**.

Planned explorative subgroups by baseline characteristics:

- Gender
- Use of a beta-blocker in their standard treatment (yes/no)
- Diabetes mellitus (yes / no)
- Atrial fibrillation (yes / no) at registration
- Age > 65 years at baseline
- BMI > 30 [kg/m2] at baseline
- Region (Poland / Germany / Other countries)

Results will be displayed as a forest plot with interaction tests as illustrated below with a dummy arm.

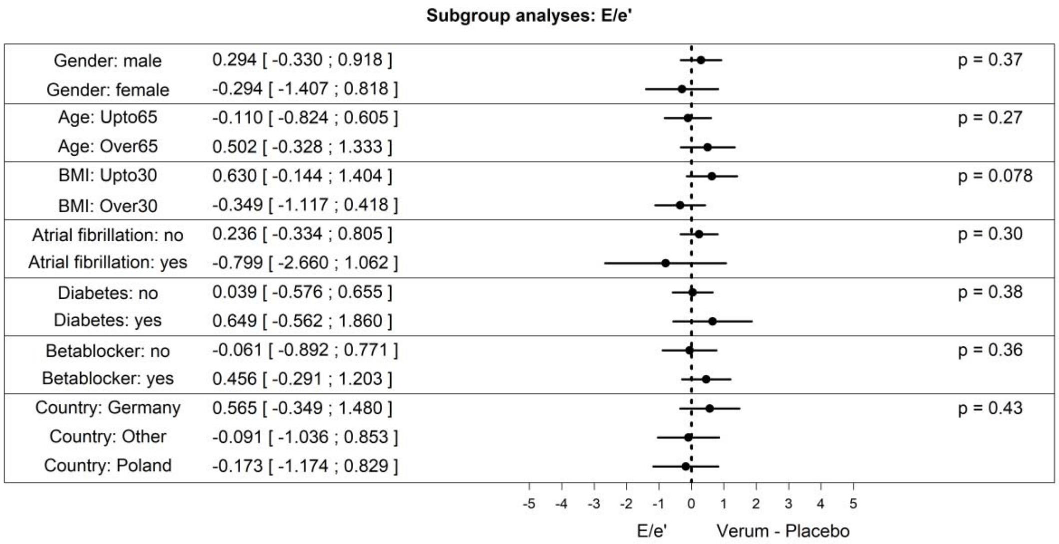

Subgroup analyses will be performed by looking at a threefold interaction:

Example for sex with an (0/1) indicator variable Male:

```
nlme::lme(Endpoint ~ Male + V3 + V3:Male + V3:Verum + V3:Verum:Male
    + V5 + V5:Male + V5:Verum + V5:Verum:Male +  AF +  DB
        random = ~ BASE + V3 + V5 - 1 | PATNO ,
            data = Beta3, method="REML")
```

The term `V5:Verum:Male` assesses the relevant interaction of sex with the treatment effect at 12 months and is used to test for the presence of a treatment by subgroup interaction.

In addition, subgroup specific estimates of the treatment effect will be provided with 95% confidence intervals.

5.6 Safety aspects

5.6.1 Adverse events

The following descriptive analyses will be provided:

- Patients with at least one AE (number and %) total and by arm
- AEs per patient – descriptive statistics (number, mean, SD, minimum, maximum) total and by arm

- AEs per month of drug exposure, total and by arm
- Characteristics of AEs (seriousness, relatedness, severity, outcome, measures regarding study drug) number and % of all adverse events, total and by arm
- AEs by body system resp. preferred term: number of adverse events total and by arm
- AEs by body system: number of patients with at least one event, total and by arm,
- AEs by preferred term: number of patients with at least one event, total and by arm

5.6.2 Serious adverse events

The following descriptive analyses will be provided:

- SAEs by body system resp. preferred term: number of adverse events total and by arm
- SARs (serious adverse reactions) by body system resp. preferred term: number of adverse events total and by arm
- Listings of all serious adverse events

5.6.3 Events of special interest

The following descriptive analyses will be provided:

<u>Blood pressure</u>

- Patients with at least one event of elevated blood pressure, total and by arm,
- Patients with at least one event of highly elevated blood pressure, total and by arm,
- Listing of all patients with elevated blood pressure
- Individual time course of systolic and diastolic blood pressure, by arm (in particular for patients with elevated blood pressure)

<u>Hepatic impairment</u>

- Patients with at least one event of hepatic impairment, total and by arm,
- Listing of all patients with hepatic impairment
- Individual time course of ALT [ULN] and AST [ULN], by arm (in particular for patients with hepatic impairment), displayed on the log10 scale

<u>Renal impairment</u>

- Patients with at least one event of renal impairment, total and by arm,
- Listing of all patients with renal impairment
- Individual time course of eGFR [ml/min] by arm (in particular for patients with renal impairment)

<u>Anaemia</u>

- Patients with at least one event of anaemia, total and by arm,
- Listing of all patients with anaemia
- Individual time course of Hb [g/dl] by arm (in particular for patients with anaemia)

<u>Atrial fibrillation / flutter</u>

- Patients with at least one event of atrial fibrillation, total and by arm,
- Listing of all patients with atrial fibrillation

<u>Deaths</u>

- Listing of all deceased patients

6 References

Bundgaard H.; Axelsson Raja, A.; Iversen, K.; Valeur, N; Tønder, N.; Schou, M.; Hørby Christensen, A.; Eske Bruun, N.; Søholm, H.; Ghanizada, M.: Fry, N.A.S.; Hamilton, E.J.; Boesgaard, S.; Møller, M.B.; Wolsk, E.; Rossing, K.; Køber, L.; Rasmussen, H.H.; Vissing, C.R.: Hemodynamic Effects of Cyclic Guanosine Monophosphate-Dependent Signaling Through β3 Adrenoceptor Stimulation in Patients With Advanced Heart Failure: A Randomized Invasive Clinical Trial. Circ Heart Fail 2022 Jul;15(7):e009120.

DuBois D, DuBois EF: A formula to estimate the approximate surface area if height and weight be known (1916). Archives of Internal Medicine. 17, 1916, S. 863–871.

Fitzmaurice, Garrett M.; Laird, Nan M.; Ware, James H. (2004): Applied Longitudinal Analysis, Book: Wiley-Interscience, Hoboken, New Jersey.

Garcia-Lunar L.; Blanco, I. ; Fernández-Friera, L. ; Gonzàlez, S. ; Jordà, P.; Sánchez, P.; Pereda, D.; Pujadas S.; Rivas M.; Solé-Gonzalez, E.; Vázquez J.; Blázquez, Z.; García-Picart J.; Caravaca, P.; Escalera, N.; Garcia-Pavia, P.; Delgado, J.; Segovia-Cubero J.; Fuster V.; Roig, E.; Albert Barberá, J.; Ibanez, B.; García-Álvarez, A.: Design of the β3-Adrenergic Agonist Treatment in Chronic Pulmonary Hypertension Secondary to Heart Failure Trial. JACC Basic Transl Sci 2020 Mar 11;5(4):317-327.

Hochberg, Yosef (1988): A sharper Bonferroni procedure for multiple tests of significance. In: Biometrika 75 (4), S. 800–802.

McNeish, Daniel (2017) Small Sample Methods for Multilevel Modeling: A Colloquial Elucidation of REML and the Kenward-Roger Correction, Multivariate Behavioral Research, 52:5, 661-670.

Mallincrodt, Craig H.; Lane, Peter W.; Schnell, Dan; Peng, Yahong; Mancuso, James P. (2008): Recommendations for the Primary Analysis of Continuous Endpoints in Longitudinal Clinical Trials. In: Drug Information Journal (42), S. 303–319

Wonisch M, Hofmann P, Pokan R, Kraxner W, Hödl R, Maier R, Watzinger N, Smekal G, Klein W, Fruhwald F. (2003). Spiroergometrie in der Kardiologie-Grundlagen der Physiologie und Terminologie. Journal für Kardiologie-Austrian Journal of Cardiology, 10(9), 383-390.

Yamamoto, Kazuhiro; Origasa, Hideki; Hori, Masatsugu (2013): Effects of carvedilol on heart failure with preserved ejection fraction: the Japanese Diastolic Heart Failure Study (J-DHF). In: European journal of heart failure 15 (1), S. 110–118.

7 Appendix

7.1 Abbreviations

ABPM	Ambulatory blood pressure monitoring
AE	Adverse Event
AF	Atrial Fibrillation
ALT	Alanine aminotransferase
AR	Adrenergic Receptor
AST	Aspartate aminotransaminase
BMI	Body Mass Index
BPM	Beats per Minute
BSA	Body Surface Area
CA	Competent Authority
cMRI	cardiac MRI
CRF	Case Report Form
CTC	Clinical Trial Centre

DSMB	Data Safety and Monitoring Board
EAC	Event Adjudication Committee
EC	Ethics Committee
eCRF	Electronic Case Report Form
EF	Ejection Fraction
eGFR	Estimated glomerular filtration rate
GCP	Good Clinical Practice
Hb	Hemoglobin
HCT	Hematocrit
HFpEF	Heart failure with preserved ejection fraction
HFrEF	Heart failure with reduced ejection fraction
HR	Heart Rate
ICH	International Conference on Harmonisation
ICMJE	International Committee of Medical Journal Editors
ISF	Investigator Site File
LAVOLI	Left atrial volume index
LGE	Left Gadolinium Enhancement
LV	Left Ventricle
LVM	Left Ventricular Mass
LVMI	Left Ventricular Mass Index
MHRD	Maximum recommended human dose
OAB	Overactive Bladder
REML	Restricted maximum likelihood estimation
SAE	Serious Adverse Event
SAR	Serious Adverse Reaction
SUSAR	Suspected Unexpected Serious Adverse Reaction
TMF	Trial Master File
V1-5	Visits 1-6, at 1, 3, 6, 9, and 12 months after randomisation
WOCB	Women of child bearing potential
