# Supplementary material for: Repurposing the β3-Adrenergic Receptor Agonist Mirabegron in Patients With Structural Cardiac Disease: The Beta3-LVH Phase 2b Randomized Clinical Trial
Source: JAMA Cardiol. 2023 Sep 20;8(11):1031–40. doi: 10.1001/jamacardio.2023.3003 (PMC10512168; doi:10.1001/jamacardio.2023.3003)
Supplement: Supplement 2. — eMethods eResults eReference eTable 1. Participant Inclusion and Exclusion Criteria eTable 2. Compliance With Study Medication eTable 3. Model-Estimated Means of Left Ventricular Mass Index (LVMI) and E/e′ Over Time in the Mirabegron and Placebo Groups eTable 4. Model-Estimated Treatment Effect on Left Ventricular Mass Index (LVMI) and E/e′ (Difference vs Placebo) eTable 5. Adverse Events of Special Interest eTable 6. Twenty-Four–Hour Ambulatory Blood Pressure Monitoring eFigure 1. Trial Flow Chart and Schedule of Assessments eFigure 2. Change in Primary Outcomes Over Time in the Mirabegron and Placebo Groups: Per-Protocol Analysis eFigure 3. Comparative Effect of Mirabegron and Placebo on the Main Prespecified Secondary Outcomes eFigure 4. Comparative Effect of Mirabegron and Placebo on Other End Points eFigure 5. Substudy on Endothelial Function [file jamacardiol-e233003-s002.pdf]

Supplementary Online Content

Balligand JL, Brito D, Brosteanu O, et al. Repurposing the β_3-adrenergic receptor agonist mirabegron in patients with structural cardiac disease: the Beta3-LVH phase 2b randomized clinical trial. *JAMA Cardiol*. Published online September 20, 2023. doi:10.1001/jamacardio.2023.3003

eMethods

eResults

eReference

eTable 1. Participant Inclusion and Exclusion Criteria

eTable 2. Compliance With Study Medication

eTable 3. Model-Estimated Means of Left Ventricular Mass Index (LVMI) and E/e′ Over Time in the Mirabegron and Placebo Groups

eTable 4. Model-Estimated Treatment Effect on Left Ventricular Mass Index (LVMI) and E/e′ (Difference vs Placebo)

eTable 5. Adverse Events of Special Interest

eTable 6. Twenty-Four–Hour Ambulatory Blood Pressure Monitoring

eFigure 1. Trial Flow Chart and Schedule of Assessments

eFigure 2. Change in Primary Outcomes Over Time in the Mirabegron and Placebo Groups: Per-Protocol Analysis

eFigure 3. Comparative Effect of Mirabegron and Placebo on the Main Prespecified Secondary Outcomes

eFigure 4. Comparative Effect of Mirabegron and Placebo on Other End Points

eFigure 5. Substudy on Endothelial Function

This supplementary material has been provided by the authors to give readers additional information about their work.

© 2023 Balligand JL et al. *JAMA Cardiology*.

eMethods

Manufacturing of study medication: Manufacturing of mirabegron and placebo was carried out at the Hospital Clinic Pharmacy of Leipzig University (Universitätsklinikum Leipzig, AoeR, Apotheke, Liebigstrasse 20, Leipzig, Germany). Mirabegron bought from Astellas Pharma (Tokyo, Japan) and placebo were re-conditionned in capsules of exactly similar appearance, introduced in polyethylene bottles and labeled as study medication with a batch number and expiration date. After shipment to the clinical trial centers, they were kept in required conservation conditions (ambient temperature <25°C).

Allocation concealment: Randomization was produced by a statistician not otherwise involved with the trial at the Biometrics Department of Universität Leipzig. Patients and staff were masked to the identity of the trial drug, which was administered in accordance with the randomization procedure.

Procedures. A subgroup of patients underwent a [18F]-FDG-Positron Emission Tomographic and CT-scan assessment of beige/brown fat abundance and activity, as well as measurement of endothelial function by post-occlusive digital microplethysmography (fingertip peripheral arterial tonometric device, Endo-PAT; Itamar, IL). They were then randomized to one treatment arm, and re-evaluated at 1, 3, 6, 9 and 12 months. Evaluations at 6 and 12 months included all of the above, except ABPM and [18F]-FDG-PET and CT scans which were only repeated at 12 months; evaluations at intermediate visits were restricted to clinical assessment at 1, 3 and 9 months, and urine and blood sampling at 9 months. All visits (beyond baseline) included an assessment of adherence to the allocated treatment (counting of used/returned medication) and documentation of adverse effects. During the COVID-19 pandemic, depending on individual country's restrictions, we anticipated that some patients might have been unable or reluctant to attend research visits in person. Therefore, some intermediate evaluations at 1 or 9 months were conducted by telephone. A final follow-up assessment by telephone call was done for all patients one month after treatment completion, i.e. at 13 months.

Outcomes

In addition to primary and key secondary outcomes, additional secondary endpoints included further assessments of focal cardiac fibrosis (late gadolinium enhancement, by cMRI), LV EF, HOMA_beta-cell function, fasting serum glycemia, insulin, total-, LDL-, HDL-cholesterol, triglycerides, hs-TnT, GDF-15 and Galectin-3 at 3, 6 and 12 months. A subset of patients underwent additional measurements of endothelial function by digital tonometry and of beige/brown fat activation by Positron Emission Tomography/CT scan.

Statistics

A 3-dimensional random effect with a general unstructured variance covariance matrix is used to model the dependence of measurements within-patients. The model is fitted using the "nlme" R-package using the formula:

nlme::lme(Endpoint ~ V3 + V5 + V3:Verum + V5:Verum + AF + DB ,
random = ~ BASE + V3 + V5 - 1 | PATNO,
data = Beta3, method="REML")

The patients' time courses are modelled as a joint three-dimensional normal distribution with fixed effects as mean and a variance-covariance matrix capturing the intra-patient dependance.
This means that e.g., even if the 12-month value is missing, Baseline and 6-month values influence the likelihood estimate of the 12 months mean through their leverage via the variance-covariance matrix.

Not including "**BASE:Verum**" in the model results in more statistical power (1))

Exploratory subgroup analyses for the two primary endpoints were also performed by baseline characteristics including: gender, use of beta-blocker in standard treatment, diabetes mellitus, atrial fibrillation at registration, age >65 yrs; BMI >30 kg/m2 at baseline, and region (Poland/Germany/Other countries)

© 2023 Balligand JL et al. *JAMA Cardiology*.

eResults

Patient population

From the FAS population, 63 patients (31 in the mirabegron arm; 32 in the placebo arm) were excluded from the per protocol set (PPS) because of major protocol violations. In the mirabegron arm, an eligibility criterion was violated in 7 patients, 2 patients had a relative dose below 50%, and a mix-up of study drug happened in 1 patient; in a further 21 patients, both LVMI and E/e' measurements were missing at the 12-month visit. In the placebo group, an eligibility criterion was violated in 8 patients, 1 patient had a relative dose below 50%, 1 patient cumulated both violations, and a mix-up of study drug happened in 1 patient; in a further 21 patients, both LVMI and E/e' measurements were missing at the 12-month visit.

Adverse events

A total of 61 serious AEs were reported, 31 in the mirabegron group (in 19 patients), and 30 in the placebo group (in 22 patients). Of them, 18 events were considered to be related to the study medication by two independent evaluators, 12 in 9 patients in the mirabegron group (5 increased blood pressure, 2 elevated ALT, 3 paroxysmal atrial fibrillation in one patient, 2 paroxysmal atrial flutter in one patient); and 6 in 6 patients in the placebo group (3 increased blood pressure, 1 abnormal glomerular filtration rate, 1 atrial flutter, 1 atrial fibrillation).

Study medication was discontinued because of SAE in 3 patients in the mirabegron group (granular cell tumour, prostate cancer, ALT increase) and 4 patients in the placebo group (joint arthroplasty, anemia and sepsis in one patient; increased blood pressure; coronary artery disease; myocardial infarction).

Sub-study on endothelial function

101 patients in the mirabegron arm and 108 patients in the placebo arm underwent measurements of endothelial function at baseline. Of these, 81 and 63 underwent subsequent measurements at 6 months and 12 months, respectively, in the mirabegron arm; and 83 and 74 patients at 6 months and 12 months in the placebo arm. Mirabegron had a neutral effect on endothelial function and augmentation index measured by digital microtonometry, as the baseline and covariates adjusted differences at 12 months between mirabegron and placebo was -0.046 [95% confidence interval (CI): -0.141; 0.048; p=0.335] in the Reactive Hyperemia Index (lnRHI) (eFig 5a); and +5.228 [-0.068; 10.58; p=0.053] in augmentation index (eFig 5b).

Sub-study on beige/brown fat

Nine patients in the mirabegron arm and 12 patients in the placebo arm underwent measurements of beige/brown fat by [^{18}F-FDG]-PET and CT scan at baseline. Of these, 9 and 9 patients underwent subsequent measurements at 12 months in the mirabegron arm and in the placebo arm, respectively. In none of these patients was beige/brown fat detectable by PET-CT at baseline. No increase in ^{18}FDG uptake was detected in either treatment arm after 12 months.

eReference

1. Fitzmaurice, Garrett M; Laird, Nan M.; Ware, James H, eds. Applied Longitudinal Analysis (chapter 5.7) Hoboken, New Jersey: Wiley-Interscience, 2004

© 2023 Balligand JL et al. *JAMA Cardiology*.

eTable 1. Participant Inclusion and Exclusion Criteria

**Inclusion criteria**
Age between 18 and 90 years
Morphological signs of structural cardiac remodelling by echocardiography, that is, increased LV mass index (95 g/m^2 or higher for female; 115 g/m^2 or higher for male subjects or end-diastolic wall thickness ≥13 mm in at least one wall segment
Written informed consent: for subjects unable to read and/or write, oral informed consent observed by an independent witness is acceptable if the subject has fully understood oral information given by the investigator. The witness should sign the consent form on behalf of the subject.
Note: patients are allowed to take a $\beta_{1–2}$-blocker, other than the drugs listed in the exclusion criteria
**Exclusion criteria**
Uncontrolled hypertension with systolic BP ≥160 mmHg and/or diastolic BP ≥100 mmHg (confirmed at three consecutive office measurements in sitting position); if so, the patient may be re-screened after optimization of anti-hypertensive treatment.
Hypertensive patients not under stable therapy according to current guideline algorithm (including stable medication for at least 4 weeks before inclusion)
Documented ischemic cardiac disease defined as follows: current angina pectoris, ischaemia on stress test, untreated coronary stenosis >50%, history of AMI, CABG (<3 months prior to screening), or PTCA less than 3 months prior to screening.
Patients with uncontrolled recurrent persistent and permanent AF according to AHA/ACC/ESC guidelines (with a HR >100 per minute, RACE II). If AF with HR >100 per minute, the patient may be re-screened after treatment for rate control.
History of hospitalization for overt heart failure within last 12 months
Patients after heart transplantation
History of high-degree impulse conduction blocks (greater than second-degree AV block Type 2)
Genetic hypertrophic or dilated cardiomyopathy
EF <50%, regardless of symptoms
Significant valvulopathy (less than 1 cm^2 aortic valve area or significant mitral valve insufficiency at Doppler echocardiography) and/or previous valvular surgery
Congenital valvulopathies
Patients with a known history of QT prolongation (QT >450 ms) or patients with documented QT prolongation (QT >450 ms) while taking medicinal products known to prolong the QT interval
NYHA Class >II
BMI ≥ 40 kg/m^2
Hyperthyroidism/hypothyroidism
Known other cause (i.e. COPD) of respiratory dysfunction. Patients under positive pressure (CPAP) treatment for sleep apnea syndrome may be included, provided they have been efficiently controlled under regular treatment for at least 1 year before inclusion in the study
Moderate renal impairment defined as eGFR <30 mL/min
Abnormal liver function tests (AST or ALT >2× upper normal limit or patients with known hepatic impairment defined as Child–Pugh Class B or higher)
Type I diabetes, complicated Type II diabetes (i.e. with documented coronary macroangiopathy, *cfr* exclusion criterion 1, or documented other vascular complication)
Patients with anemia (male: Hb <13.0 g/L; female: Hb <12.0 g/L)
Patients with bladder outlet obstruction
Patients using antimuscarinic cholinergic drugs for treatment of OBD

© 2023 Balligand JL et al. *JAMA Cardiology*.

Exclusion criteria (continued)

Current use of digitalis, bupranolol, propranolol, and nebivolol (known to interfere with β_3AR signaling)

Patients continuously treated with sildenafil or other PDE5 inhibitors

Current use of antifungal azole derivatives (fluconazole, itraconazole, miconazole, posaconazole, and voriconazole) (known inhibitors of CYP3A4, metabolizer of mirabegron)

Current treatment with mirabegron or indication for future treatment with mirabegron due to other indications

Contraindication for MRI (e.g. defibrillator, ferromagnetic devices, or severe claustrophobia)

Pregnant or nursing women

Participation in any other interventional trial: patients unable to give informed consent (people under legal guardianship)

Women of child-bearing potential without highly effective contraceptive measures

Contra-indication to mirabegron (e.g. hypersensitivity)

eTable 2. Compliance With Study Medication

		**Mirabegron**		**Placebo**				
		N	%	N	%	Total N	%	p-value
**Relative Dose**	<50%	6	4.1	6	4.0	12	4.1	
	50-80%	22	15.0	25	16.8	47	15.9	
	>80%	119	81.0	118	79.2	237	80.1	
	N Valid	147	100.0	149	100.0	296	100	0.913

eTable 3. Model-Estimated Means of Left Ventricular Mass Index (LVMI) and E/e′ Over Time in the Mirabegron and Placebo Groups

		**Baseline (pool)**	**6 Months**		**12 Months**	
			**Mirabegron**	**Placebo**	**Mirabegron**	**Placebo**
**LVMi**	Est.Mean	59.6	60.0	59.7	60.2	58.9
(g/m^2)	C.I.	(58.2-60.9)	(58.5-61.5)	(58.2-61.2)	(58.7-61.8)	(57.4-60.5)
**E/e’**	Est Mean	9.5	9.3	9.3	9.4	9.5
	C.I.	(9.2-9.9)	(8.8-9.7)	(8.9-9.8)	(8.9-9.8)	(9.0-10.0)

eTable 4. Model-Estimated Treatment Effect on Left Ventricular Mass Index (LVMI) and E/e′ (Difference vs Placebo)

		**Difference**	**Lower C.I**	**Upper C.I.**	**p-value**
**LVMi**	6 Months	0.247	-1.13	1.6	0.724
(g/m^2)	12 Months	1.3	-0.15	2.74	0.079
**E/e’**	6 Months	-0.035	-0.58	0.51	0.898
	12 Months	-0.147	-0.694	0.40	0.597

© 2023 Balligand JL et al. *JAMA Cardiology*.

eTable 5. Adverse Events of Special Interest

		Mirabegron		Placebo		All		
		N	%	N	%	N	%	p-value
**Systolic and diastolic blood pressure under treatment**								
Blood pressure (SBP or DBP)	Normal	127	85.8	129	87.2	256	86.5	
	Elevated	19	12.8	14	9.5	33	11.1	
	Highly Elevated	2	1.4	5	3.4	7	2.4	
	N valid	148	100	148	100	296	100	0.357
Systolic BP	Normal	135	91.2	135	91.2	270	91.2	
	Elevated	11	7.4	10	6.8	21	7.1	
	Highly Elevated	2	1.4	3	2.0	5	1.7	
	N valid	148	100	148	100	296	100	0.884
Diastolic BP	Normal	136	91.9	137	92.6	273	92.2	
	Elevated	12	8.1	8	5.4	20	6.8	
	Highly Elevated	0	0.0	3	2.0	3	1.0	
	N valid	148	100	148	100	296	100	0.149
**Liver Enzymes under treatment**								
ALT or AST >2 ULN	No	143	96.6	146	98.6	289	97.6	
	Yes	5	3.4	2	1.4	7	2.4	
	N valid	148	100	148	100	296	100	0.444
**Renal Impairment under treatment**								
eGFR < 30mL/min	No	148	100	147	99.3	295	99.7	
	Yes	0	0	1	0.7	1	0.3	
	N valid	148	100	148	100	296	100	0.661

eTable 6. Twenty-Four–Hour Ambulatory Blood Pressure Monitoring

		Baseline			12 Months		
		**Mirabegron**	**Placebo**	**P value**	**Mirabegron**	**Placebo**	**P value**
**Mean systolic BP (mmHg)**	**24 hours**	131±12	132±13	0.37	130±12	131±14	0.63
	**Day**	134±12	136±13	0.26	133±13	135±15	0.48
	**Night**	122±14	121±14	0.67	122±16	120±15	0.49
**Mean diastolic BP (mmHg)**	**24 hours**	77±8	78±10	0.41	77±9	76±10	0.46
	**Day**	80±9	81±10	0.29	80±9	78±10	0.42
	**Night**	70±8	69±11	0.53	70±10	67±10	0.08

© 2023 Balligand JL et al. *JAMA Cardiology*.

eFigure 1. Trial Flow Chart and Schedule of Assessments

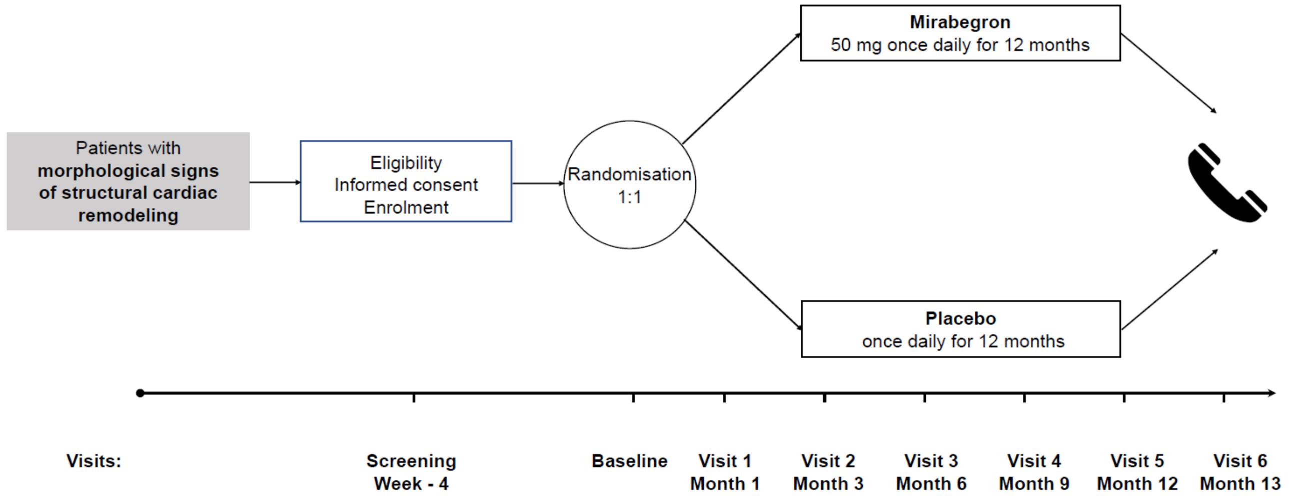

© 2023 Balligand JL et al. *JAMA Cardiology*.

eFigure 2. Change in Primary Outcomes Over Time in the Mirabegron and Placebo Groups: Per-Protocol Analysis

eFigure 2a. Left Ventricular Mass Index (LVMI)

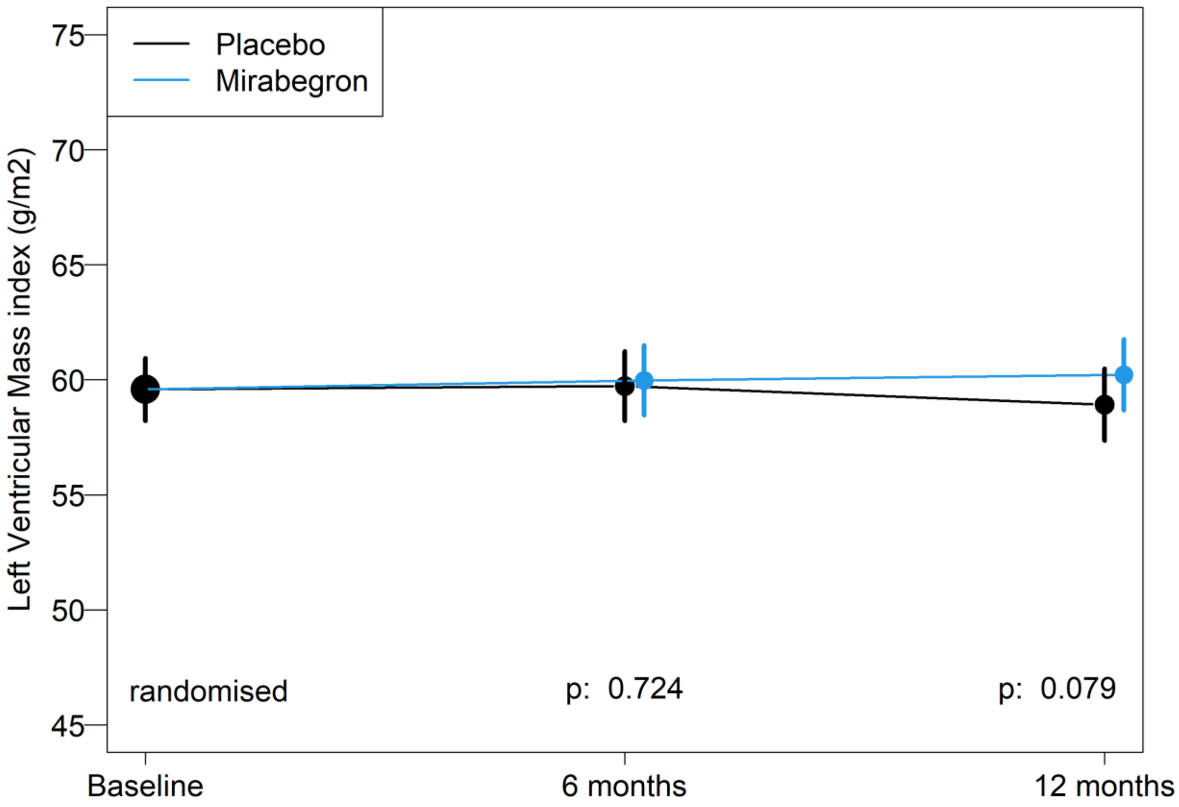

© 2023 Balligand JL et al. *JAMA Cardiology*.

eFigure 2b. E/e′

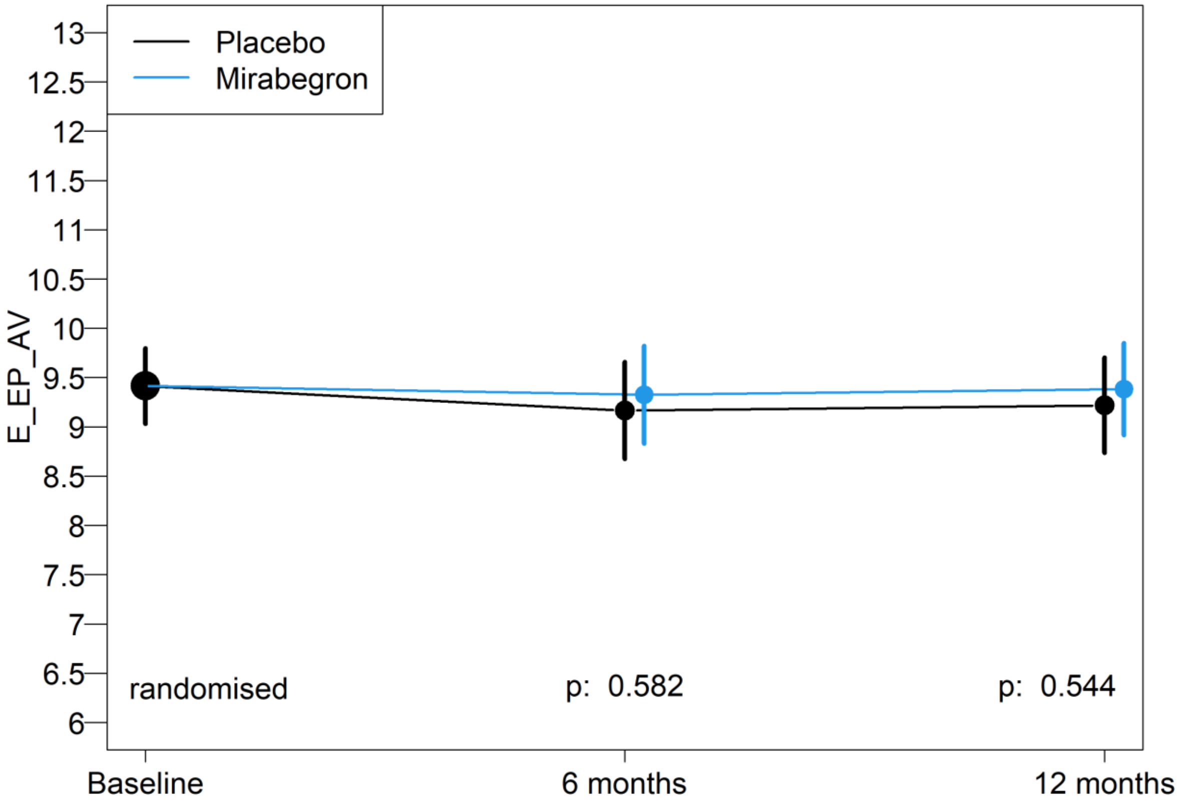

eFigure 2: Treatment-specific changes at post-baseline visits in LVMi (eFig 2a) and E/e' (eFig 2b) deduced from the basic linear mixed model (see Methods) in the Per-Protocol group. As the groups are randomized, the mean at baseline is not estimated separately by arm. *P* values refer to treatment differences tested against zero.

© 2023 Balligand JL et al. *JAMA Cardiology*.

eFigure 3. Comparative Effect of Mirabegron and Placebo on the Main Prespecified Secondary Outcomes

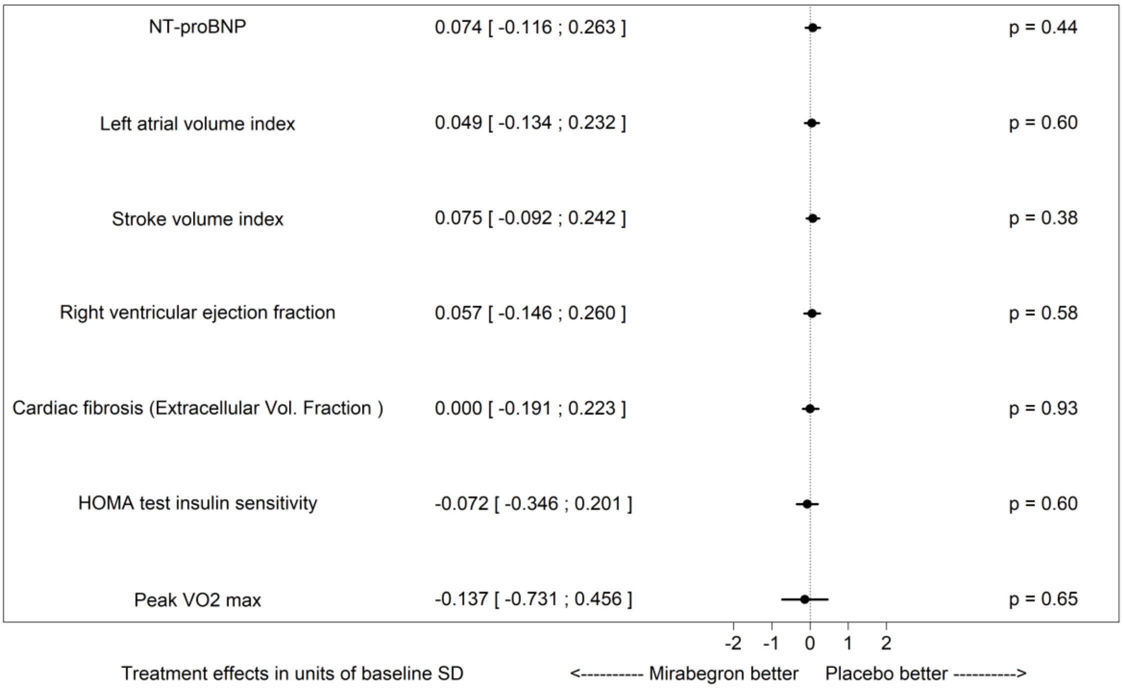

eFigure 3 shows the model estimated treatment effect at 12 months of mirabegron versus placebo for the seven pre-specified key secondary outcomes. Treatment effects with 95% confidence intervals are provided using the pooled baseline standard deviation as unit, allowing to show all endpoints on the same effect size scale.

eFigure 4. Comparative Effect of Mirabegron and Placebo on Other End Points

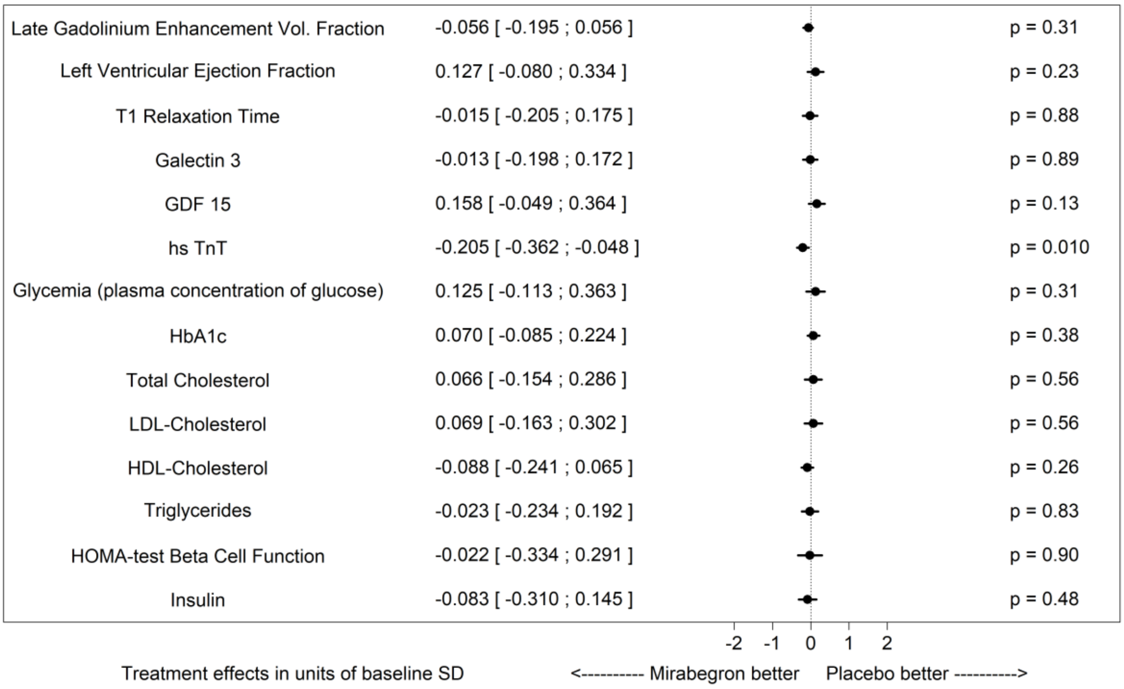

eFigure 4 shows the model estimated treatment effect at 12 months of mirabegron versus placebo for the 14 other secondary outcomes. Treatment effects with 95% confidence intervals are provided using the pooled baseline standard deviation as unit, allowing to show all endpoints on the same effect size scale.

© 2023 Balligand JL et al. *JAMA Cardiology*.

eFigure 5. Substudy on Endothelial Function

eFigure 5a. Log Reactive Hyperemia Index: Model Plot

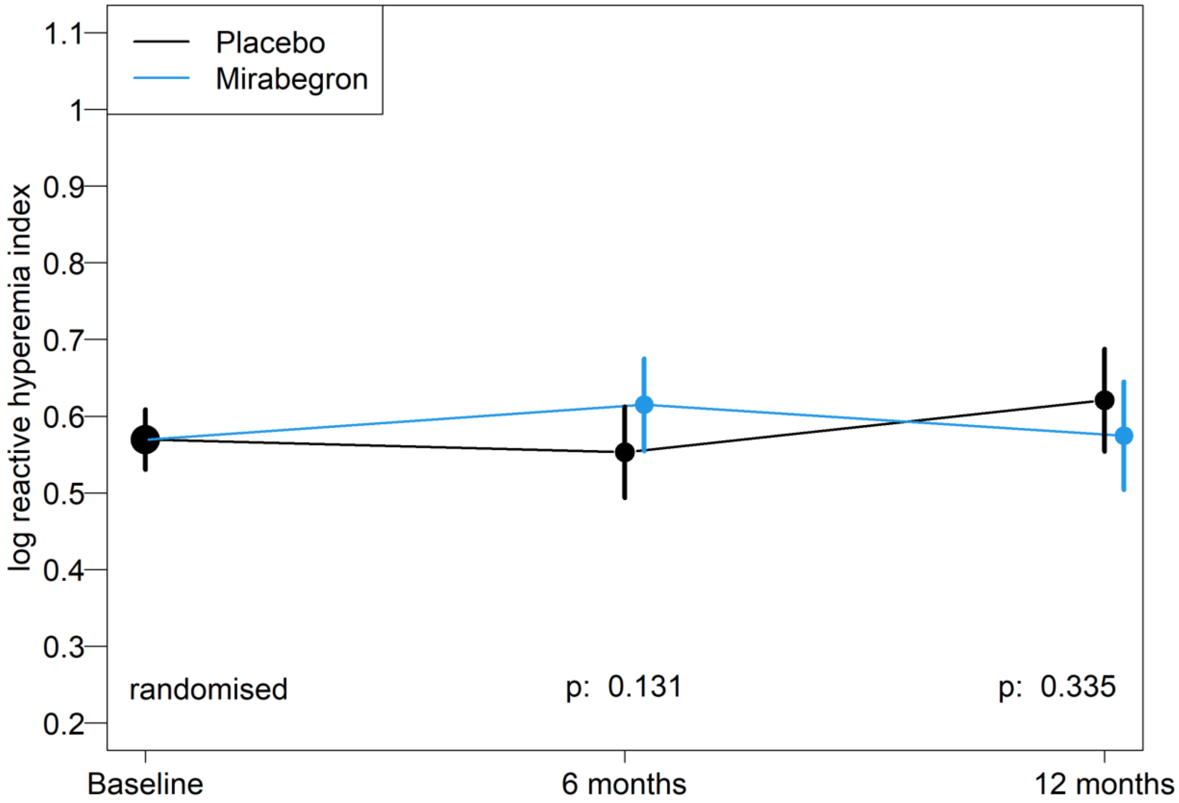

© 2023 Balligand JL et al. *JAMA Cardiology*.

eFigure 5b. Augmentation Index: Model Plot

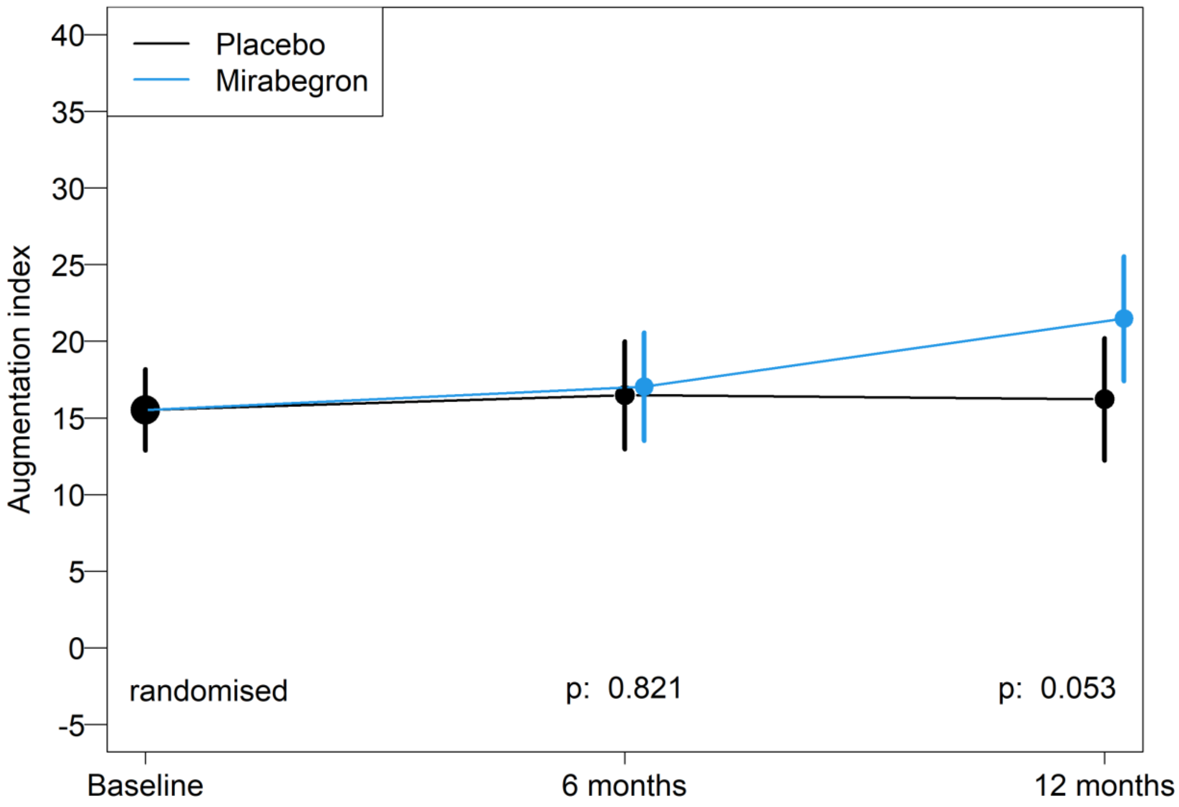

eFigure 5: Treatment-specific differences at post-baseline visits in Log Reactive Hyperemia Index (lnRHI) (eFig 5a) and Augmentation Index (eFig 5b) deduced from the basic linear mixed model (see Methods). As the groups are randomized, the mean at baseline is not estimated separately by arm. *P* values refer to treatment differences tested against zero.

© 2023 Balligand JL et al. *JAMA Cardiology*.
