# Supplementary material for: Repurposing the β3-Adrenergic Receptor Agonist Mirabegron in Patients With Structural Cardiac Disease: The Beta3-LVH Phase 2b Randomized Clinical Trial
Source: JAMA Cardiol. 2023 Sep 20;8(11):1031–40. doi: 10.1001/jamacardio.2023.3003 (PMC10512168; doi:10.1001/jamacardio.2023.3003)
Supplement: Supplement 3. — Data Sharing Statement [file jamacardiol-e233003-s003.pdf]

Data Sharing Statement

Balligand. Repurposing the β_3-Adrenergic Receptor Agonist Mirabegron in Patients With Structural Cardiac Disease. *JAMA Cardiol*. Published September 20, 2023. doi:10.1001/jamacardio.2023.3003

Data
Data available: Yes
Data types: Deidentified participant data
How to access data: Individual participant data required to reach aims in an approved proposal, after deidentification, will be made available to investigators whose proposed use of the data has been approved by the study's Executive Committee. Proposals may be submitted up to 36 months after article publication and should be directed to jean-luc.balligand@uclouvain.be.
When available: With publication

Supporting Documents
Document types: Statistical/analytic code
How to access documents: jean-luc.balligand@uclouvain.be
When available: With publication

Additional Information
Who can access the data: researchers whose proposed use of the data has been approved by the Steering committee of BETA3LVH
Types of analyses: for the purpose of verifying an hypothesis deemed valid by the Steering Committee of BETA3LVH
Mechanisms of data availability: After approval of a proposal
